# Mechanistic evaluation and transcriptional signature of a glutathione *S*-transferase omega 1 inhibitor

**DOI:** 10.1038/ncomms13084

**Published:** 2016-10-05

**Authors:** Kavya Ramkumar, Soma Samanta, Anahita Kyani, Suhui Yang, Shuzo Tamura, Elizabeth Ziemke, Jeanne A. Stuckey, Si Li, Krishnapriya Chinnaswamy, Hiroyuki Otake, Bikash Debnath, Vladimir Yarovenko, Judith S. Sebolt-Leopold, Mats Ljungman, Nouri Neamati

**Affiliations:** 1 Department of Pharmacology and Pharmaceutical Sciences, School of Pharmacy, University of Southern California, 1985 Zonal Avenue, Los Angeles, California 90033, USA; 2 Department of Medicinal Chemistry, College of Pharmacy, University of Michigan, 2800 Plymouth Road, Building 520, Room 1363, Ann Arbor, Michigan 48109, USA; 3 Translational Oncology Program, University of Michigan, 2800 Plymouth Road, Building 520, Room 1363, Ann Arbor, Michigan 48109, USA; 4 Department of Radiology, University of Michigan Medical School, Ann Arbor, Michigan 48109, USA; 5 Life Sciences Institute, University of Michigan, Ann Arbor, Michigan 48109, USA; 6 N.D. Zelinsky Institute of Organic Chemistry, Russian Academy of Sciences, 47 Leninsky prospect, 119991 Moscow, Russia; 7 Department of Pharmacology, University of Michigan Medical School, Ann Arbor, Michigan 48109, USA; 8 Department of Radiation Oncology, University of Michigan, Ann Arbor, Michigan 48109, USA

## Abstract

Glutathione *S*-transferase omega 1 (GSTO1) is an atypical GST isoform that is overexpressed in several cancers and has been implicated in drug resistance. Currently, no small-molecule drug targeting GSTO1 is under clinical development. Here we show that silencing of GSTO1 with siRNA significantly impairs cancer cell viability, validating GSTO1 as a potential new target in oncology. We report on the development and characterization of a series of chloroacetamide-containing potent GSTO1 inhibitors. Co-crystal structures of GSTO1 with our inhibitors demonstrate covalent binding to the active site cysteine. These potent GSTO1 inhibitors suppress cancer cell growth, enhance the cytotoxic effects of cisplatin and inhibit tumour growth in colon cancer models as single agent. Bru-seq-based transcription profiling unravelled novel roles for GSTO1 in cholesterol metabolism, oxidative and endoplasmic stress responses, cytoskeleton and cell migration. Our findings demonstrate the therapeutic utility of GSTO1 inhibitors as anticancer agents and identify the novel cellular pathways under GSTO1 regulation in colorectal cancer.

## Abstract (web summary)

Glutathione S-transferase omega 1 (GSTO1) is an atypical GST isoform overexpressed in several cancers that has been implicated in drug resistance. Here the authors identify a small molecule inhibitor of GSTO1 that effectively inhibits tumor growth in colon cancer models, and establish its mechanism of action.

## Graphical abstract

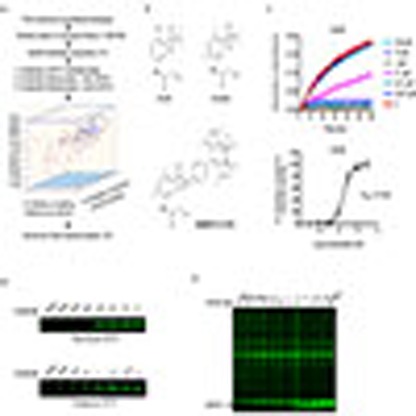

Glutathione *S*-transferases (GSTs) are a diverse family of cytosolic, mitochondrial and microsomal enzymes that are primarily involved in phase II metabolism. In addition to xenobiotic detoxification through glutathione conjugation, GSTs also play a key role in the synthesis and metabolism of endogenous compounds, redox homeostasis and cellular signalling^1^,^2^. GST overexpression and polymorphisms are seen in several cancers as well as other diseases, and have been implicated in resistance to chemotherapy^2^,^3^. Thus, they are attractive anticancer targets. Indeed, several small-molecule GST inhibitors have been reported as anticancer agents^4^.

The mammalian cytosolic GSTs are classified into seven different classes—alpha, mu, pi, theta, sigma, zeta and omega. Among them, the omega GSTs (GSTO) belong to an atypical cytosolic class. They share low sequence identity with other GST classes, but still exhibit the characteristic GST fold^5^,^6^. GSTOs have a cysteine residue at the catalytic site in place of the characteristic tyrosine or serine. Consequently, instead of glutathione conjugation, GSTOs catalyse thioltransferase reactions similar to glutaredoxins. In humans, two GSTO isozymes GSTO1 and GSTO2 have been identified. They share 64% sequence identity and differ in their catalytic activities. GSTO1 catalyses *S*-phenacyl glutathione reduction and monomethylarsonate (V) reduction, while GSTO2 has a prominent dehydroascorbate reductase activity^7^.

The cellular functions of GSTO1 have not been fully elucidated, although a role in cellular stress responses is emerging^8^,^9^,^10^. Recently, a significant role for GSTO1 in protein deglutathionylation has been described^11^, and β-actin was identified as one of the cellular substrates for GSTO1-catalysed deglutathionylation. Studies have also proposed a role in ryanodine receptor modulation, interleukin-1β (IL-1β) secretion and endoplasmic reticulum stress-mediated autophagy^7^,^12^,^13^,^14^,^15^. Interestingly, GSTO1 polymorphisms and decreased expression have been implicated in several pathological conditions such as Parkinson's and Alzheimer's disease^7^. In addition, GSTO1 overexpression has been documented in breast cancer cell lines^16^,^17^ and has a role in the development of drug resistance^7^,^18^,^19^. Thus, GSTO1 is an attractive pharmacological target, for which, there is a need for potent and selective inhibitors.

Small molecules and peptides bearing electrophilic moieties have previously been reported as GSTO1 inhibitors^20^,^21^,^22^,^23^,^24^. In particular, compounds containing α-chloroacetamide moieties have shown potent inhibitory activity. However, characterization of their effects in cancer cells is limited. Here we report on the development and characterization of small-molecule inhibitors of GSTO1 and the use of these inhibitors to study the cellular functions of GSTO1. We have identified a family of chloroacetamide-containing compounds with potent inhibitory activity against GSTO1 and efficacy against cancer cells in both *in vitro* and *in vivo* models. In addition, these compounds inhibited IL-1β secretion from monocytic cells. We further show using siRNA that GSTO1 is an important survival factor for cancer cells. Through transcriptional profiling using Bru-seq, we also uncover novel cellular pathways regulated by GSTO1. Taken together, our findings validate GSTO1 as an important drug target for cancer therapeutics. Importantly, we have solved the crystal structure of our potent inhibitors in complex with GSTO1, paving the way for rational design of optimized GSTO1 inhibitors.

## Results

### Identification of C1-27 as a potent GSTO1 inhibitor

The α-chloroacetamide is a privileged scaffold that has been commonly used to target cysteine residues on various proteins^20^,^21^,^25^. We synthesized a set of novel small molecules based on a 2-chloro-*N*-phenylacetamide scaffold, evaluated their effects on GSTO1 activity and explored structure–activity relationships (Supplementary Fig. 1). For activity screening, we adapted a GSTO1 substrate assay that relies on the quantification of the reduction of *S*-(4-nitrophenacyl)glutathione (4-NPG), a GSTO1-specific substrate^17^, using recombinant human GSTO1 in a high-throughput screening format. The *Z*′ factor value for the assay was 0.73 (Supplementary Fig. 2). **4a**, one of the initial hits, inhibited GSTO1 enzyme activity with a half-maximal inhibitory concentration (IC_50_) value of 3.1 μM. We observed that addition of electron-withdrawing substituents on the phenyl ring increased the electrophilicity of the chloroacetamide carbon and improved potency of inhibition (Supplementary Table 1).

**Figure 1:**
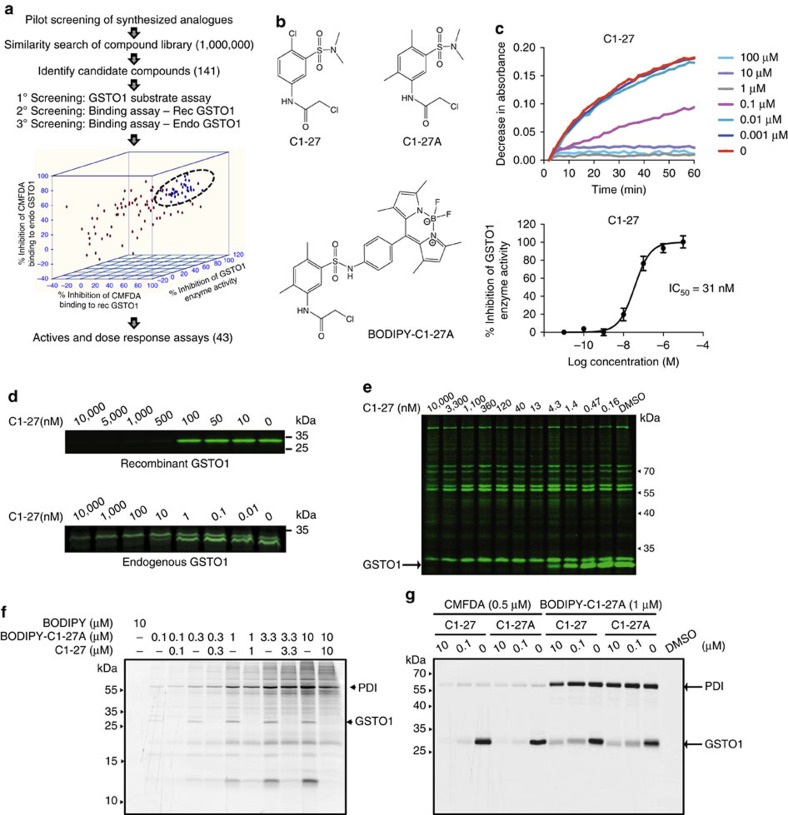
Identification and characterization of C1-27 as a potent GSTO1 inhibitor. (**a**) Schematic of small-molecule screening and inhibitor identification. (**b**) Chemical structures of lead inhibitor **C1-27**, related analogue **C1-27A** and BODIPY-conjugated **C1-27A**. (**c**) **C1-27** significantly inhibited GSTO1-catalysed 4-nitrophenacyl glutathione reduction in a dose-dependent manner. (Below) Plot shows % inhibition of GSTO1 activity by **C1-27**, calculated from absorbance values at *T*=30 min. Curves generated from mean values of five independent experiments (error bars, s.e.m.). (**d**) **C1-27** potently competes with CMFDA for binding to recombinant GSTO1 (top) and endogenous GSTO1 in HCT116 soluble proteome (bottom). Competitive inhibition of CMFDA binding was assessed by in-gel fluorescence scanning. One of three independent experiments is shown. (**e**) **C1-27** competes with CMFDA for binding to GSTO1 without affecting labelling of other proteins, demonstrated by in-gel fluorescence binding assay. One of three independent experiments is shown. (**f**) Fluorescence scan of proteins labelled by BODIPY-**C1-27A** in HCT116 cells in the presence and absence of pretreatment with **C1-27** at indicated concentrations. Representative image of two independent experiments is shown in greyscale for clarity. Arrows indicate bands corresponding to GSTO1 and PDI. (**g**) **C1-27** or **C1-27A** potently inhibited BODIPY-**C1-27A** and CMFDA binding to recombinant GSTO1 while modestly inhibiting PDI labelling. Representative image of two independent experiments is shown.

We next performed a structural similarity search across our in-house compound collection, as well as on commercial small-molecule libraries (∼1,000,000 compounds) and identified 141 additional hits, which were screened in the high-throughput activity assay at a single concentration of 10 μM. Their GSTO1 inhibitory activity was further confirmed by a competitive binding assay. Using 5-chloromethylfluorescein diacetate (CMFDA), which has been reported to be a potent and irreversible GSTO1 inhibitor^22^, we optimized a gel-based binding assay that measures competitive inhibition of CMFDA binding to recombinant GSTO1 or endogenous GSTO1 in a soluble proteome (Supplementary Fig. 3). The general approach for inhibitor identification is outlined in Fig. 1a. On the basis of their structures, the hits were grouped into five different clusters (Supplementary Figs 4–8). Among the different clusters, cluster-1, based on a *N*-phenyl-2°-chloroacetamide scaffold, yielded some of the most potent active compounds. Replacing the phenyl ring with a heterocyclic moiety was also well tolerated and resulted in potent hits (cluster-2). Cluster-3 consisting of *N-*linker-2°-chloroacetamides was mostly inactive, suggesting that inclusion of additional linker groups between the chloroacetamide moiety and the aromatic centre decreased its electrophilic nature and negatively affected potency. Similarly, replacing the 2°-chloroacetamide with a 3°-chloroacetamide derivative such as in cluster-4 also proved unfavourable and resulted in loss of potency. On the other hand, cyclization of the 3°-chloroacetamide restored activity (cluster-5). From this preliminary screening, we selected 43 compounds that showed at least 50% inhibition in all three assays for further dose–response studies (Supplementary Tables 2–6). Inhibitory profile of selected potent compounds is shown in Supplementary Table 7. Interestingly, some of the top hits from this large unbiased screen belonged to the *N*-(3-sulfamoyl)phenyl chloroacetamide class of compounds. Among them, **C1-27** was one of the most potent GSTO1 inhibitors (Fig. 1b). **C1-27** potently inhibited GSTO1 enzyme activity with an IC_50_ value of 31 nM (Fig. 1c). It also potently competed with CMFDA for binding to recombinant protein, as well as endogenous GSTO1 in the milieu of a soluble proteome (Fig. 1d). While the fluorescent dye CMFDA labelled several other cysteine-containing proteins in the proteome, **C1-27** showed a selective inhibition of the GSTO1 band up to 1 μM (Fig. 1e). To further examine **C1-27** selectivity and to detect other possible targets, we synthesized a fluorescent BODIPY conjugate of a **C1-27** analogue. Attempts at generating BODIPY-conjugated **C1-27** were unsuccessful as it proved to be unstable. Instead, we used **C1-27A**, a close structural analogue with a similar inhibitory profile (Fig. 1b and Supplementary Fig. 9). Analysis of the HCT116 soluble proteome showed that BODIPY-**C1-27A** bound GSTO1 even at sub-micromolar concentrations. In addition, BODIPY-**C1-27A** also strongly labelled a 57 kDa band corresponding to protein disulfide isomerase (PDI), a chaperone protein with cysteine at its active site (Fig. 1f and Supplementary Fig. 10a). Compounds containing α-chloroacetamide moieties have been shown to bind PDI^25^. Above 1 μM, BODIPY-**C1-27A** showed an increased nonspecific reactivity that was not inhibited by pretreatment with unconjugated **C1-27A** (Supplementary Fig. 10b). Similarly, pretreatment with **C1-27** selectively blocked BODIPY-**C1-27A** binding to GSTO1 up to 1 μM. Furthermore, **C1-27** bound GSTO1 more potently than PDI and showed a 100-fold more potent inhibition of its enzyme activity (Fig. 1f,g and Supplementary Table 7). In a cellular thermal-shift assay, we observed GSTO1 engagement by **C1-27** even at lower concentrations (1 μM), while a thermal shift for PDI was observed only at higher concentrations (Supplementary Fig. 11). Other potent GSTO1 inhibitors also showed a similar selectivity profile (Supplementary Fig. 10c and Supplementary Table 7). These findings suggest that despite bearing a reactive electrophile, **C1-27** did not bind to cellular proteins indiscriminately but appeared to exhibit certain degree of selectivity in target binding.

### Co-crystal structures of inhibitors in complex with GSTO1

**Figure 2:**
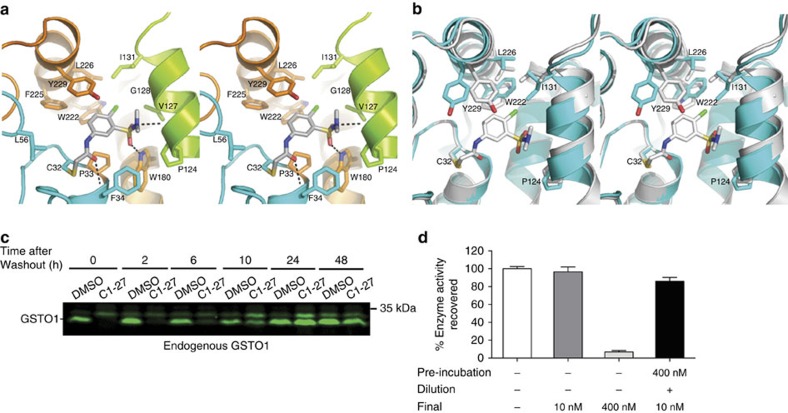
C1-27 is a covalent GSTO1 inhibitor. (**a**) Stereodiagram of **C1-27** bound to GSTO1 (PDB ID 4YQM). Ribbon diagram represents the backbone of GSTO1 with residues interacting with the inhibitor shown as ball-and-stick. Residues in cyan contribute to the G-site, while the H-site is made of the residues shown in orange and green (4b helix). The inhibitors are shown in ball-and-stick with carbon atoms in grey, sulfurs in yellow, nitrogens in blue, oxygens in red and chloride in green. The black dashed lines represent hydrogen bonds between the protein and the inhibitor. (**b**) The crystal structure of GSTO1-**C1-27** (grey) overlayed onto GSTO1-GSH (cyan) (PDB ID 1EEM) with the residues having the largest change shown in ball-and-stick. The overall root mean squared deviation between the GSTO1-**C1-27** and 1EEM structures is 0.614 Å. (**c**) CMFDA labelling of GSTO1 was inhibited by **C1-27** up to 6 h and recovered completely by 24 h. One of three independent experiments is shown. (**d**) **C1-27** acts as a slow-turnover substrate demonstrated by 86% recovery of enzyme activity after pre-incubation and a large dilution in the GSTO1 substrate assay. Experiments were performed in triplicate (error bars, s.e.m.).

We determined the structures of GSTO1 bound to three inhibitors (**C1-27**, **C1-31** and **C4-10**) by X-ray crystallography (Fig. 2a and Supplementary Fig. 12). In each case, difference electron density maps contoured at 3*σ* show each inhibitor covalently bound to the Sγ atom of C32 in GSTO1 (Supplementary Fig. 13). All three inhibitors bind primarily to the hydrophobic or electrophile binding site (H-site)^5^ of GSTO1, but do so in unique ways (Supplementary Fig. 14). **C1-31** and **C4-10** bind solely through hydrophobic interactions, whereas **C1-27** binding incorporates both hydrophobic and hydrophilic interactions. In biochemical assays, **C1-27** had the highest affinity for GSTO1, which is supported by the structure in that **C1-27** makes more interactions with the protein than the other two inhibitors.

The structure of the GSTO1–**C1-27** complex was solved to 2.4 Å resolution with three molecules per asymmetric unit. The bound **C1-27** interacts predominately with residues in the H-site with only three interactions with the glutathione-binding site (G-site) (Fig. 2a). The backbone amide nitrogen of F34 from the G-site stabilizes the acetamide oxygen position via a hydrogen bond and the phenyl ring of F34 packs edge on with the phenyl group of **C1-27**. The third interaction is between the side chain of L56 (Cδ1) and the carbon atom of the acetamide group. The rest of the atoms within **C1-27** interact with the H-site causing rearrangement of several side chains and a 1 Å shift in the 4b helix (residues 122–132) with respect to the GSTO1-GSH structure (1EEM) (Fig. 2b). The side chains of W222 and I131 rotate 180° and 90°, respectively, compared with the 1EEM structure due to the binding of the **C1-27** chloride atom. The chloride is located in a hydrophobic pocket interacting with I131 Cδ1, V127 Cγ1 and W222 Cζ. The side chain of Y229 also differs from the 1EEM structure, swinging >49° to form a partial *π*-stacking with the phenyl group of the inhibitor. The entire 4b helix shifts ∼1 Å in comparison to the 1EEM structure due to the sulfonamide group interacting with three residues (G128, V127 and P124) within the helix. These residues form hydrophobic interactions with the methyl groups, while the carbonyl O of P124 hydrogen bonds with the nitrogen atom, which conforms to a tetrahedral configuration suggesting that it is protonated. The O2 oxygen of the sulfonamide accepts a hydrogen bond from W180. The other H-site residues (L226, F225 and P33) form van der Waals interactions with the phenyl group of **C1-27**. Similar structural rearrangements in W222 and Y229 have been reported with the binding of GSSG in the H-site^26^.

The GSTO1–**C1-31** structure was determined to 1.94 Å resolution with two molecules in the asymmetric unit (Supplementary Fig. 12a). The position of the acetamide group of **C1-31** is stabilized by hydrophobic interactions with the side chains of G-site residues L56 and V72. The side chains of F34 and P33 interact with the bottom of the phenyl ring. The seven-membered ring is flanked on three sides by Y229, I131, L226 and F225. The only interaction with the sulfonamide group is V127. The side chains of all residues in the binding site are reminiscent of the 1EEM structure. The GSTO1–**C4-10** structure solved to 2.10 Å resolution has two residues (Y229 and W222) that differ from the 1EEM structure. Y229 is positioned in an equivalent manner as in the GSTO1–**C1-27** structure, but it does not interact with **C4-10**, therefore is not shown in Supplementary Fig. 12b. W222 is rotated and pushed aside to accommodate the phenyl group of **C4-10**, which is inserted deeply into the protein surrounded by W222, F225, L226, I131, M187, R183 and V127. The pyrazole group interacts with P33, while the side chains of residues M29 and L56 from the G-site interact with the acetamide group.

In line with the covalent binding mechanism, *in vitro* and cell-based thermal-shift assays showed that **C1-27** induced a negative shift in the melting temperature indicating protein destabilization on binding (Supplementary Figs 11 and 15). To further examine the nature of GSTO1–inhibitor complexes, we analysed the time course of GSTO1 labelling by CMFDA following pretreatment of HCT116 cells with **C1-27**. GSTO1 labelling was inhibited up to 6 h and then recovered completely by 24 h (Fig. 2c). **4a**, on the other hand, was more resistant to the washout and inhibited CMFDA labelling up to 24 h (Supplementary Fig. 16a). We further examined **C1-27** dissociation and GSTO1 reactivation using a pre-incubation and dilution assay. Recombinant GSTO1 was pre-incubated with **C1-27** at 400 nM, followed by a 40-fold dilution into assay buffer to a final concentration of 10 nM. Interestingly, 86% of GSTO1 activity was recovered (Fig. 2d), suggesting that **C1-27** acts as a slow-turnover substrate. Other close analogues of **C1-27** also showed a similar regeneration of GSTO1 activity on dilution (Supplementary Fig. 16d). The mechanism of enzyme regeneration is unclear, although we speculate that presence of a reducing agent such as dithiothreitol (DTT) in the assay buffer could facilitate the recovery. In contrast, enzyme activity following **4a** incubation was not recovered on dilution, indicating an irreversible covalent bond with the enzyme (Supplementary Fig. 16c).

### GSTO1 inhibition is cytotoxic to cancer cells

**Figure 3:**
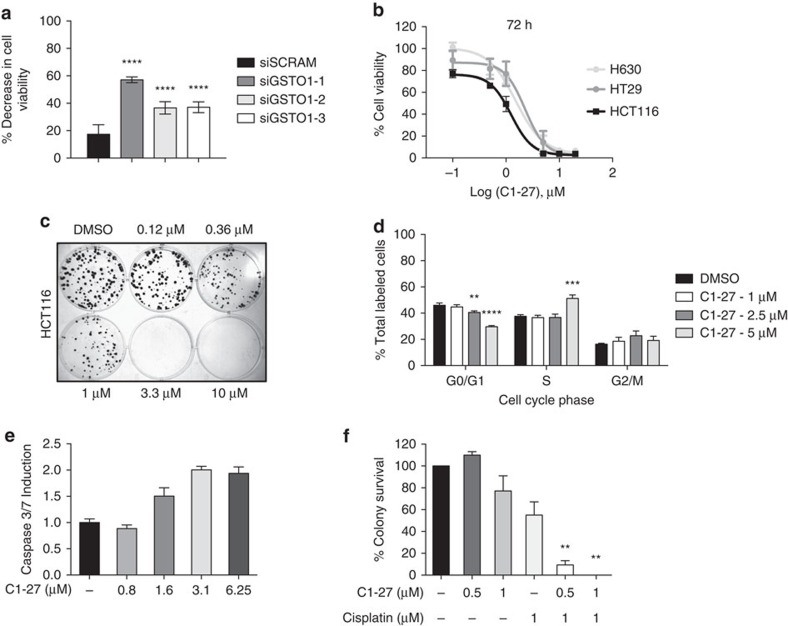
GSTO1 inhibition impedes cancer cell viability. (**a**) GSTO1 knockdown decreases HCT116 cell viability. In all, 4,000 cells per well were transfected with three different GSTO1-specific siRNAs (10 nM) and cell viability was assessed by MTT assay. Data are mean of 10 replicate wells (error bars, s.e.m.). (**b**) **C1-27** inhibited cancer cell viability, as measured by MTT assay. Data are mean±s.d. of two independent experiments with duplicate wells. (**c**) **C1-27** significantly inhibited clonogenicity of HCT116 cells. Representative colony plate of three independent experiments is shown. (**d**) Cell cycle progression in HCT116 cells, treated with 5 μM **C1-27** for 24 h, was analysed by flow cytometry. Mean per cent of propidium iodide-labelled cells in each cell cycle phase calculated from three independent experiments (error bars, s.d.). (**e**) Induction of apoptosis by 24 h **C1-27** treatment, assessed by ApoTox-Glo triplex assay. Data are mean of triplicate wells (error bars, s.d.). (**f**) **C1-27** enhances cisplatin-induced cytotoxicity. HCT116 cells were treated with indicated concentrations of **C1-27** and cisplatin for 24 h. Per cent colony survival from three independent colony formation assay experiments was assessed (error bars, s.d.). **P<0.01; ***P<0.001; ****P<0.0001, analysed by two-tailed, unpaired Student's *t*-test assuming unequal variance.

We evaluated the expression of GSTO1 in various tumour types using publicly available gene expression data sets from the Oncomine database. GSTO1 was significantly overexpressed in colorectal, head and neck, breast and oesophageal cancers, as well as in melanomas and lymphomas (Supplementary Fig. 17a and Supplementary Table 8). Similarly, we observed an upregulation of GSTO1 mRNA in different cancer cell lines (HCT116, MDA-MB-435 and MDA-MB-231) by analysing transcript data in BioGPS (Supplementary Fig. 17b). To determine the significance of such overexpression, we evaluated the effect of silencing GSTO1 on cancer cell viability. Treatment with GSTO1-specific siRNA significantly decreased the viability of HCT116 cancer cells (Fig. 3a). These results were further replicated in different cancer cell lines (Supplementary Fig. 18b). HCT116 cells treated with **C1-27** also showed a decrease in cell viability in a dose-dependent manner (Fig. 3b). Other top GSTO1 inhibitors too showed cytotoxicity in a panel of cancer cell lines (Supplementary Table 9). Of note, **C1-27** inhibited the clonogenic survival of HCT116 cells at sub-micromolar concentrations (Fig. 3c). Furthermore, at sub-cytotoxic concentrations, the size of the colonies was reduced, suggesting an effect on cell proliferation. To confirm this, we analysed the effect on cell cycle progression by flow cytometry. Treatment with 5 μM **C1-27** for 24 h resulted in an increase in the S-phase population, with a concomitant decrease in the G0/G1 population (Fig. 3d). An activation of caspases was also detected (Fig. 3e). Since GSTO1 overexpression has been implicated in drug resistance, particularly to platinum compounds^7^,^18^,^19^, we tested the effects of GSTO1 inhibitors in combination with cisplatin in HCT116 cells. **C1-27** was able to significantly enhance cisplatin-induced cytotoxicity in a clonogenic assay, at non-cytotoxic concentrations (Fig. 3f). Although siRNA studies show that GSTO1 is important for HCT116 cancer cell viability, knockdown of GSTO1 did not appear to affect **C1-27** cytotoxicity, suggesting that other targets of **C1-27** also may contribute to its cytotoxicity and have a compensatory effect (Supplementary Fig. 18c).

### Gene expression signatures of C1-27 and GSTO1 knockdown

**Figure 4:**
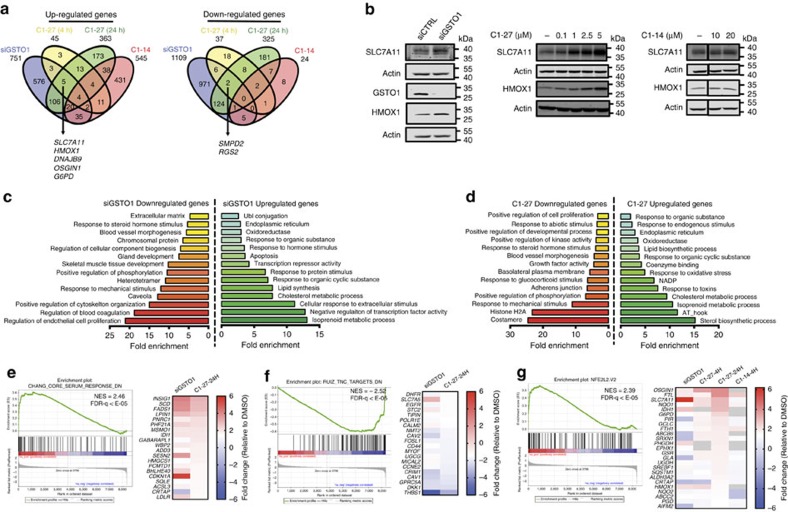
Analysis of gene expression following C1-27 treatment and GSTO1 knockdown. (**a**) Venn diagram of genes upregulated (left) and downregulated (right) over 1.4-fold or more after treatment with **C1-27** (1 μM), the inactive analogue **C1-14** (10 μM) or GSTO1 knockdown (siGSTO1), as measured by Bru-seq. (**b**) Western blotting of SLC7A11 and HMOX1 expression after 24 h treatment of HCT116 cells with siGSTO1, **C1-27** or **C1-14** at indicated concentrations. (**c**,**d**) Pathway analysis of differentially transcribed genes following GSTO1 knockdown (**c**) and **C1-27** (24 h) treatment (**d**). Functional terms represented by genes downregulated (left) and upregulated (right) by at least 1.4-fold and with a count of four or more genes, as analysed using DAVID (david.abcc.ncifcrf.gov) are shown. (**e**) GSEA plot show enrichment of genes upregulated by siGSTO1 and 24 h **C1-27** treatment in the ‘CHANG_CORE_SERUM_RESPONSE_DN'^30^ signature. (**f**) GSEA plot shows significant enrichment of genes downregulated by siGSTO1 and **C1-27** treatment in the tenascin-C-associated gene signature^41^. (**g**) GSEA plot shows enrichment of ‘NFE2L2.V2' (ref. 31) stress-response signature among genes upregulated by **C1-27** treatment. In each panel, nominal *P* values and false discovery rate-*q* values are indicated. Heatmaps depict the relative expression levels of top genes enriched in each gene set in siGSTO1-, **C1-27-** (4 or 24 h treatment) or **C1-14**-treated HCT116 cells as indicated. Grey cells denote genes with no transcription detected.

To identify pathways regulated by GSTO1 and to understand the mechanism of its antiproliferative effects, we used the nascent RNA Bru-seq method^27^ to profile gene expression changes that occur in response to GSTO1 knockdown and inhibition. In HCT116 cells treated with siGSTO1 for 24 h, 751 genes were upregulated and 1,108 genes were downregulated by over 1.4-fold, compared with control cells (Fig. 4a). *CHAC1* (cation transport regulator homologue 1) was one of the most significantly upregulated genes in response to GSTO1 knockdown (Supplementary Table 10). In addition, several stress- and metabolism-related genes such as oxidative stress-induced growth inhibitor 1 (*OSGIN1*), solute carrier family 7 member 11 (*SLC7A11*), haem oxygenase 1 (*HMOX1*) and glucose-6-phosphate dehydrogenase (*G6PD*) were also induced. Consistent with the Bru-seq results, GSTO1 knockdown markedly induced SLC7A11 and HMOX1 protein expression at 24 h (Fig. 4b). Among the genes downregulated in response to GSTO1 siRNA, coagulation factor III (*F3*), thrombospondin 1 (*THBS1*), cysteine-rich angiogenic inducer (*CYR61*) and bone morphogenetic protein 4 (*BMP4*), related to blood coagulation and blood vessel morphogenesis, were markedly decreased (Supplementary Table 11). Of note, transcription of cyclin-dependent kinase inhibitor, p21 (*CDNK1A*) was significantly upregulated in response to GSTO1 knockdown while that of the cell cycle regulatory proteins cyclin D1 (*CCND1*), cyclin E1 (*CCNE1*), as well as *Myc* were found downregulated (GEO accession No. GSE85899).

To explore the biological functions of these genes, we performed functional enrichment analysis using database for annotation, visualization and integrated discovery (DAVID)^28^. Genes that were upregulated by more than 1.4-fold in response to GSTO1 knockdown showed an enrichment of pathway terms related to lipid synthesis, sterol metabolism and transcriptional repression. In addition, pathways related to hormone stimulus, oxidative stress and endoplasmic reticulum were also enriched (Fig. 4c). Among the genes downregulated by more than 1.4-fold, a significant enrichment of functional pathways related to caveola, cytoskeleton organization, blood vessel and coagulation, as well as chromosomal proteins was observed. To further delineate pathways that are responsive to GSTO1 knockdown, we performed gene set enrichment analysis (GSEA) using the Bru-seq data (Supplementary Tables 12 and 13). Genes upregulated by GSTO1 siRNA showed significant enrichment of amino-acid deprivation response-related gene sets^29^. A wound-healing-related gene set, described by Chang *et al*.^30^, representing genes negatively regulated in the fibroblast core serum response, was also strongly enriched among the genes upregulated by GSTO1 knockdown (Fig. 4e). Genes downregulated by siGSTO1 showed an enrichment of gene sets relating to tenascin-C signalling and oestrogen response (Fig. 4f). We also examined gene expression changes induced by **C1-27** in HCT116 cells after 4 and 24 h treatment to observe the early and late response to GSTO1 inhibition, respectively (Supplementary Tables 14–17 and 20–23). By comparing these transcriptional changes with those induced by an inactive analogue, **C1-14** (Supplementary Fig. 19 and Supplementary Tables 18, 19 and 24), we further sought to discriminate between the on-target and off-target effects of **C1-27**. The early effects of GSTO1 inhibition by **C1-27** (4 h treatment) predominantly involved induction of several stress-response-associated genes (*OSGIN1*, *HMOX1*, *SLC7A11*, *DNAJB9* and so on) (Supplementary Table 14). GSEA analysis revealed a significant enrichment of a NRF2 stress-response signature^31^. Sustained GSTO1 inhibition for 24 h also resulted in a similar stress-response signature (Fig. 4g and Supplementary Table 16). Western blotting further showed that **C1-27** markedly increased SLC7A11 and HMOX1 expression, while the inactive control had no effect (Fig. 4b). More importantly, 24 h treatment of HCT116 cells with **C1-27** resulted in transcriptional changes that were more similar to those observed with GSTO1 knockdown (Fig. 4a), with <12% overlap with the inactive control, **C1-14**. A comparison of **C1-27**-treated (24 h) and GSTO1 siRNA-treated transcriptomes showed an overlap of 111 commonly upregulated genes and 126 commonly downregulated genes with more than 1.4-fold change in expression. GSEA of genes upregulated by 24 h treatment with **C1-27** likewise showed a significant enrichment of the wound-healing gene set (Fig. 4e and Supplementary Table 22). Genes downregulated after 24 h treatment with **C1-27** also showed significant enrichment of the tenascin-C signalling gene set (Fig. 4f and Supplementary Table 23). Pathway analysis of the upregulated and downregulated genes using DAVID showed an enrichment of functional terms related to cholesterol metabolism, NADP, response to oxidative stress and response to mechanical stimulus, adherens junction and blood vessel morphogenesis, respectively (Fig. 4d).

### C1-27 suppresses human colon cancer xenograft growth *in vivo*

**Figure 5:**
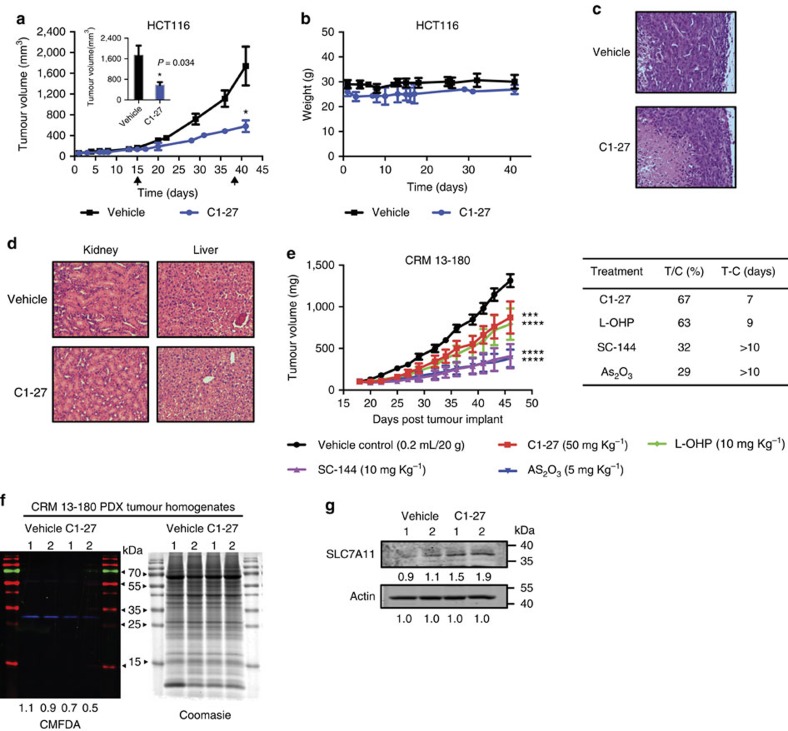
C1-27 inhibits colorectal cancer tumour growth *in vivo*. (**a**) Tumour growth curves of HCT116 tumour-bearing nude mice treated with vehicle (*n*=5) or **C1-27** (*n*=3). Data shown are mean tumour volumes (error bars, s.e.m.). Arrows indicate dose escalation (treatment schedule is described in the Methods section). Significant reduction in tumour volumes was observed on day 41 of **C1-27** treatment (*P<0.05, analysed by one-way analysis of variance (ANOVA)). (**b**) Mean body weights of **C1-27-** and vehicle-treated HCT116 tumour-bearing mice over the duration of treatment (error bars, s.e.m.). (**c**) **C1-27**-treated tumours showed extensive areas of necrosis, as seen by haematoxylin/eosin staining of tumour sections from **C1-27-** and vehicle-treated mice. Representative image of four independent sections is shown. (**d**) Haematoxylin/eosin staining of liver and kidney sections showed no gross signs of systemic toxicity. (**e**) Comparison of tumour growth curves in colorectal cancer PDX model CRM 13–180. NCR nude mice implanted with CRM 13–180 tumour fragments were treated with **C1-27**, SC-144, As_2_O_3_ or vehicle (*n*=5/group), on a 5 days on/2 days off schedule by intraperitoneal injection. L-OHP (*n*=5) was administered once a week. Data shown are mean tumour volumes (error bars, s.e.m.; ***P=0.0011; ****P<0.0001, analysed by two-way ANOVA). Table shows comparison of efficacies of treatments. *T*/*C* (%) represents the relative size of the median tumour burden of the treated (*T*) versus vehicle control (*C*) groups on the last day of treatment. *T*−C represents the difference in days for the treated (*T*) and vehicle control (*C*) tumour burden to reach a median size of 750 mg. (**f**) Fluorescence scan of BODIPY-**C1-27A** binding in CRM 13–180 tumour homogenates from vehicle and **C1-27**-treated mice. Normalized relative GSTO1 band intensities shown below. (right) Coomasie staining of the gel. (**g**) Western blotting of SLC7A11 expression in CRM 13–180 tumour homogenates from vehicle and **C1-27**-treated mice. Normalized relative densities computed using ImageJ are shown below.

To test whether **C1-27** had *in vivo* efficacy, we initially evaluated its effects in a human colon cancer cell line xenograft model. **C1-27** (20–45 mg kg^−1^) was administered as a single agent to nude mice bearing HCT116 xenografts. After 5 weeks of treatment, tumour growth was significantly inhibited in **C1-27**-treated mice compared with the vehicle-treated group (*P*<0.05) (Fig. 5a). **C1-27** treatment was generally well tolerated by mice up to 45 mg kg^−1^, with no overt signs of toxicity and no significant variations in average body weights throughout the duration of the study (Fig. 5b). Haematoxylin and eosin staining of the tumour sections from **C1-27**-treated mice showed extensive regions of necrosis compared with control (Fig. 5c). To further assess any treatment-related toxicity, kidney and liver tissue sections from control and **C1-27**-treated mice were examined by histology. Haematoxylin and eosin staining showed no gross morphological differences (Fig. 5d). We further evaluated the antitumour efficacy of **C1-27** in a more clinically relevant colorectal cancer patient-derived xenograft (PDX) model. The KRAS-mutant CRM-13-180 PDX model, which showed a high GSTO1 expression, was chosen for the study (Supplementary Fig. 20a). **C1-27** showed potent and selective inhibition of GSTO1 in these cells (Supplementary Fig. 20b). *In vivo*, **C1-27** resulted in a relatively modest but statistically significant inhibition of tumour growth (per cent treated/control (%*T*/*C*)=67), which was comparable to the tumour growth inhibition shown by oxaliplatin (L-OHP; %*T*/*C*=63) (Fig. 5e). On the other hand, arsenic trioxide (As_2_O_3_) and SC-144 showed significant tumour growth reduction (%*T*/*C*=29 and 32, respectively) and tumour growth delay (*T*−*C*>10 days). The maximum **C1-27** treatment-related weight loss during the course of the study did not exceed 12% (Supplementary Fig. 20c). We further examined *in vivo* binding of **C1-27** to GSTO1 using the PDX tumour tissue homogenates. BODIPY-**C1-27A** labelling of GSTO1 was decreased in **C1-27**-treated tumours compared with the control group (Fig. 5f). Furthermore, expression of SLC7A11, a GSTO1 and **C1-27** target gene, was increased in **C1-27**-treated tumours indicating target engagement (Fig. 5g). Taken together, these results demonstrate that **C1-27** targets GSTO1 in tumours and shows promising antitumour activity in both cell line xenograft and PDX models of colorectal cancer, without gross systemic toxicities.

## Discussion

Through extensive small-molecule screening, biochemical assays and X-ray crystallography, we have identified several potent GSTO1 inhibitors. Co-crystal structures of GSTO1 bound to inhibitors showed a covalent association with the active site cysteine (C32) with additional hydrophilic and hydrophobic interactions in the H-site. Although reactive electrophiles such as chloroacetamides are generally considered to be promiscuous in target binding, we demonstrated here using a BODIPY-labelled analogue that the chloroacetamide-based GSTO1 inhibitor **C1-27** exhibited a low degree of nonspecific reactivity. In addition to GSTO1, **C1-27** showed binding also to PDI. However, binding and enzyme inhibition studies revealed a 100-fold more potent affinity for GSTO1 than PDI. A previous study also reported a similar low random reactivity of selected reactive electrophiles^32^. Although not characterized in this study, we expect these inhibitors to also bind GSTO2, since it shares 64% sequence identity with GSTO1. *In vitro* mechanistic studies also showed that the lead compound **C1-27** acts as a slow-turnover substrate, though the significance of this mechanism is currently unclear.

GSTO1 overexpression has been previously reported in several cancers, as well as in drug-resistant cell lines^7^,^16^,^17^,^18^,^19^. We show here in a panel of cancer cell lines that GSTO1 knockdown reduced cancer cell viability. RNA expression profiling also showed a significant increase in *CDKN1A* (p21), a p53 target gene coding for a cell cycle inhibitor, together with a decrease in the transcription of *CCND1*, *CCNE1* and *Myc*. In accordance with this, pathway analysis of the RNA transcriptome identified oestrogen-mediated S-phase entry, p53 signalling and G1/S checkpoint regulation as canonical pathways significantly modulated in response to GSTO1 knockdown (Supplementary Fig. 21). Interestingly, the pro-survival function of GSTO1 in cancer cells appeared to be cancer- or cell type-specific. We observed that KRAS mutant cancer cell lines (HCT116 and H460) and MDA-MB-435, with a high GSTO1 expression, were sensitive to GSTO1 knockdown, but not MCF7 cells (Supplementary Fig. 18). Similarly, T47D cells have also previously been reported to be devoid of mature GSTO1 (ref. 33), with no effect on cell viability. We further observed that the lead GSTO1 inhibitor **C1-27** blocked cancer cell proliferation, caused cytotoxicity, inhibited cell cycle progression and increased the sensitivity of cancer cells to cisplatin. KRAS mutant cancer cell lines (HCT116 and Panc-1) were more sensitive to GSTO1 inhibition (Supplementary Table 25). Furthermore, the cell death induced by **C1-27** appeared to be Ras-signalling-dependent (Supplementary Fig. 22). It is of interest to note that a class of tertiary α-chloroacetamide-containing compounds such as RSL3 has previously been shown to induce a unique iron-dependent, oxidative, non-apoptotic cell death, selectively in KRAS-mutant cells, through covalently inhibiting glutathione peroxidase 4 (refs 34, 35, 36). Finally, **C1-27** showed *in vivo* efficacy in both HCT116 cell line xenograft and PDX models of colorectal cancer. We also noted that GSTO1 expression was higher in several colorectal cancer patient tumours as compared with normal colon tissue, consistent with our bioinformatics analysis (Supplementary Fig. 20a).

GSTO1 has been shown to play a significant role in the protein glutathionylation cycle exhibiting both glutathionylation and deglutathionylation activities^11^,^15^. Mitochondrial F_0_/F_1_ ATPase β subunit has been shown to be glutathionylated by GSTO1 (ref. 9), while β-actin has been reported to be specifically deglutathionylated by it^11^. Similarly, silencing GSTO1 or inhibition of its catalytic activity increased cellular levels of protein glutathionylation^14^. On the other hand, GSTO1 also catalysed rapid glutathionylation of cellular proteins during oxidative stress^11^,^15^. Interestingly, GSTO1 was identified as one of the potential cellular targets of piperlongumine, an electrophilic compound exerting selective cytotoxicity against cancer cells through an oxidative cell death mechanism (Supplementary Table 25)^37^. Piperlongumine and its analogues have also been shown to increase the levels of protein glutathionylation^38^. Together, these findings suggest a role for GSTO1 in regulating cell signalling and redox homeostasis through affecting the *S*-glutathionylation status on its target proteins.

While a role in cellular stress response is emerging, the cellular targets of GSTO1 and its functions have not been fully understood. To address this, we performed transcription profiling using Bru-seq and identified genes involved in cellular stress response, steroid metabolism, transcription and cytoskeleton organization as significantly altered by both GSTO1 siRNA and **C1-27**. Our data suggest that GSTO1 depletion results in cellular stress with a significant increase in the expression of various NRF2 target genes such as *OSGIN1*, *SLC7A11* and *HMOX1*. We further confirmed their expression by western blotting, thus validating the nascent RNA-profiling results. A previous study in macrophage cells also similarly showed that GSTO1 knockdown as well as GSTO1 inhibitor M175 (ref. 21) induced antioxidant genes after stimulation with lipopolysachcharide^14^. In particular, our nascent transcriptome analyses showed a significant increase in *SLC7A11* gene transcription in both **C1-27**- and GSTO1 siRNA-treated cells. *SLC7A11* encodes xCT, a cationic amino-acid transporter that is part of the cysteine- and glutamate-specific cationic antiporter, system X_c_^−^. Its transcription is regulated by NRF2 and system X_c_^−^ inhibition. We also observed a 23-fold increase in *CHAC1* transcription with GSTO1 knockdown but not with **C1-27** treatment. CHAC1 functions as a γ-glutamylcyclotransferase catalysing glutathione degradation^39^. It is also induced during endoplasmic reticulum stress, regulated by the ATF4-CHOP branch of the unfolded protein response, and elicits an apoptotic response^40^. Since GSTO1 knockdown has been known to result in increased protein glutathionylation^14^, glutathione depletion by CHAC1 could possibly contribute in restoring the glutathionylation balance. Pathway analysis further identified cholesterol metabolism (*INSIG1*, *FDFT1*, *SREBF1*, *LDLR* and so on) as being modulated by both **C1-27** and GSTO1 knockdown. Interestingly, many of these genes involved in cholesterol homeostasis were also implicated in a gene signature related to the fibroblast core serum response^30^.

GSTO1 knockdown as well as the small-molecule GSTO1 inhibitor **C1-27** also caused downregulation of the Wnt inhibitor Dickkopf 1 (*DKK1*), thombospondin 1 (*THBS1*), the cysteine-rich angiogenic inducer *CYR61* and caveolae scaffolding proteins *CAV1* and *CAV2*, among others. GSEA analysis further revealed significant enrichment of a tenascin-C-induced gene signature. Tenascin-C is an extracellular matrix glycoprotein that modulates cell adhesion and migration^41^. Since GSTO1 has been known to cause de-glutathionylation of β-actin impeding its polymerization into filamentous actin^11^, such a downregulation of molecules involved in cytoskeleton organization and adhesion is particularly interesting. Of note, GSTO1 was identified as one of the cellular targets of locostatin, an oxazolidinone-based cell migration inhibitor, although its role in cell migration has not been well characterized^42^. While the mechanism by which GSTO1 interacts with these genes and pathways remains to be fully characterized, GSTO1 catalytic activity appears to be important for this regulation.

In addition to these cellular functions, GSTO1 has been shown to play a significant role in the regulation of IL-1β processing and secretion^12^,^13^. While the exact mechanism is not fully understood, GSTO1 appears to be associated with the early responses in the pro-inflammatory pathway, influencing oxidative and metabolic changes^43^. Although beyond the scope of discussion in this paper, we found that **C1-27** and several of the potent GSTO1 inhibitors inhibited the secretion of mature IL-1β from activated monocytic cells (Supplementary Fig. 23). We further observed that pretreatment with **C1-27** inhibited the transcriptional induction of pro-IL-1β in response to inflammatory stimulus such as the bacterial endotoxin, lipopolysachcharide. These findings further extend the utility of GSTO1 inhibitors as anti-inflammatory agents. Inhibition of IL-1β secretion by GSTO1 inhibitors also has exciting implications on tumour progression and tumour-immune cell crosstalk in the microenvironment.

In conclusion, we have shown that GSTO1 is a novel and exciting target for developing anticancer and anti-inflammatory agents. Our nascent RNA expression studies suggest, for the first time, novel roles for GSTO1 in cell signalling. GSTO1 inhibitors show efficacy against cancer cells in both *in vitro* and *in vivo* models and enhance the cytotoxic effects of cisplatin. Moreover, our potent inhibitors serve as valuable tools to investigate the role of GSTO1 in cancer and other pathologies, as well as to uncover additional functions in the cell. Further studies are in progress to design clinically viable candidates through structure-based drug design approaches using the co-crystal structures of our lead inhibitors.

## Methods

### Compounds for high-throughput screening

For the similarity search and expanded screening, a diverse library of small-molecule compounds (∼1,000,000) from Asinex, Enamine and in-house collection was used. Compounds from commercial sources were purchased with a minimum purity of 90% and were stored as 50 mM stock in dimethylsulfoxide (DMSO) at −80 °C. The lead compound **C1-27** was procured from Enamine (97% purity) and further characterized using mass spectrometry (electrospray ionisation mass spectrometry (*m*/*z*): [M]^+^ calcd. for C_10_H_12_Cl_2_N_2_O_3_S, 309.99; found, 309.16). Substrate for GSTO1 enzymatic assay, 4-NPG was synthesized as previously described^17^. Product purity was confirmed by mass spectrometry (electrospray ionisation mass spectrometry (*m*/*z*): [M]^+^ calcd. for C_18_H_23_N_4_O_9_S, 471.12; found, 471.04) and Ellman's reagent (unreacted residual glutathione <1%). Synthesis and characterization of compounds used in pilot screening and target validation are detailed in Supplementary Methods.

### Cell lines

Colon cancer cell line HCT116 was generously provided by Dr Bert Vogelstein, Johns Hopkins Medical Institutions, Baltimore, MD. Pancreatic cancer cell line Panc-1 was obtained from Dr Alan Epstein, University of Southern California. All other cell lines were purchased from the American Type Culture Collection (Manassas, VA). MDA-MB-435 cell line was used in the GSTO1 knockdown experiment as it had high GSTO1 expression (Supplementary Fig. 17). We also tested active compounds in NCI-ADR/Res cell line since GSTO1 is implicated in drug resistance. Cell lines were cultured in RPMI 1640 or Dulbecco's minimal essential media supplemented with 10% fetal bovine serum at 37 °C in a humidified atmosphere of 5% CO_2_. All cell lines were maintained in culture under 30 passages and tested regularly for *Mycoplasma* contamination using PlasmoTest (InvivoGen, San Diego, CA, USA).

### Reagents

The following reagents were purchased from Cell Signaling Technology: MEK inhibitor (PD98059); PI3K inhibitor (LY294002); and JNK inhibitor (SP600125). Cisplatin was purchased from Sigma. CellTracker Green (CMFDA) was purchased from Invitrogen (Thermo Fisher Scientific). Erastin and Piperlongumine were purchased from Sigma and LKT labs, respectively. L-OHP and As_2_O_3_ were purchased from BioTang Inc. SC-144 is a GP130 inhibitor previously identified by our lab^44^. Catalogue numbers and dilutions of antibodies used in this study are summarized in Supplementary Table 26.

### Drugs and formulations for *in vivo* experiments

**C1-27** and SC-144 were dissolved in DMSO to make a stock solution. Aliquots of the stock solutions were stored at −20 °C. On each day of treatment, an aliquot of each compound was thawed and diluted using propylene glycol (60% total volume) and 0.9% normal saline (30% total volume). In the HCT116 xenograft study, **C1-27** was diluted using peanut oil. Arsenic trioxide (As_2_O_3_) was dissolved in 1 N NaOH to make a 50 mg ml^−1^ stock solution and stored at 4 °C. On each day of treatment, the As_2_O_3_ stock solution was diluted in saline and the pH adjusted using 1 N HCl. L-OHP was stored at −20 °C as a powder and was dissolved in 5% glucose on each day of treatment.

### Bioinformatics analysis

GSTO1 gene expression in normal and cancer tissues was analysed using Oncomine cancer microarray database (www.oncomine.org) across various cancer subtypes using a cancer versus normal differential analysis. Within the same cancer subtype, meta-analysis was done to compare different studies and assess overall significance of GSTO1 expression. Data sets used in the study are summarized in Supplementary Table 8. Cancer subtypes with more than one study, *P*<0.05 and fold change >1.5 were used as inclusion criteria for further analysis. For correlation analysis, normalized median-centred values for GSTO1 and IL-1β expression in each microarray study was plotted and Pearson's correlation coefficient was computed using Prism 6.0. GSTO1 mRNA expression in different cancer cell lines was assessed using BioGPS (www.biogps.org).

### siRNA experiments

GSTO1 siRNA (Trilencer-27) and scrambled control siRNA were purchased from Origene (Rockville, MD). Cells were transfected with GSTO1 siRNA (siGSTO1-1, siGSTO1-2 and siGSTO1-3) or scrambled control siRNA (siSCRAM) using Lipofectamine RNAiMAX (Invitrogen, Thermo Fisher Scientific) following the manufacturer's instructions. GSTO1 knockdown in HCT116 cells was confirmed by in-gel fluorescence using CMFDA (500 nM) 48 and 72 h post transfection. Effect on cell viability was assessed 72 h after transfection with GSTO1 siRNA or siSCRAM by 3-(4,5-dimethylthiazol-2-yl)-2,5-diphenyltetrazolium bromide (MTT) assay and normalized to mock-transfected control (transfection reagent).

### Expression and purification of GSTO1 for screening

Human GSTO1-1 plasmid was a kind gift from Dr Philip Board, John Curtin School of Medical Research, Australian National University. Recombinant GSTO1-1 was expressed in *Escherichia coli* M15 (Rep4) cells (Qiagen) and purified as described previously^5^,^45^. Briefly, GSTO1-1 pQE30 plasmid was transformed into M15 cells, which were grown in Luria-Bertani broth supplemented with 100 μg ml^−1^ ampicillin and 25 μg ml^−1^ kanamycin at 37 °C to an OD_600_ of ∼0.8 and induced with 0.1 mM isopropyl thio-*β*-D-galactoside for 4 h. Bacterial cells were collected by centrifugation at 4,000*g* for 20 min at 4 °C and the cell pellet was resuspended in buffer (50 mM NaH_2_PO_4_ and 300 mM NaCl, pH 6.0). Cells were lysed by sonication and the cleared supernatant was purified using Ni-NTA resin. The purified enzyme was dialysed against 20 mM Tris-HCl and 60 mM NaCl, pH 8.0. Protein purity was assessed by SDS–PAGE.

### Expression and purification of GSTO1 for crystallization

For crystallization, GSTO1 residues 1–241 were cloned into an expression construct containing an N-terminal 6xHis tag followed by a tobacco etch virus (TEV) protease cleavage site and expressed in *E. coli* Rosetta 2(DE3) cells. The cells were lysed in 25 mM Tris-HCl (pH 7.5), 200 mM NaCl, 0.1% β-mercaptoethanol, 10 mM MgCl_2_, 2.1 mM leupeptin, 1.53 mM aprotinin and 50 U of benzonase (Novagen). Cleared lysate was incubated with Ni-NTA resin (Qiagen) at 4 °C for 1 h. The column was washed with 25 mM Tris (pH 7.5), 200 mM NaCl and 10 mM imidazole, and eluted with 25 mM Tris (pH 7.5), 200 mM NaCl and 500 mM imidazole. Eluate incubated with TEV protease was dialysed against 25 mM Tris (pH 7.5), 150 mM NaCl and 1 mM DTT overnight at 4 °C, then incubated with Ni-NTA resin for 1 h to remove the His-tagged protease. The Ni-NTA flow-through was concentrated and applied to a Superdex 200 column equilibrated with 25 mM Tris 7.5, 60 mM NaCl and 5 mM DTT. The purified protein was concentrated to 2 mg ml^−1^ and stored at −80 °C.

### Crystallization and structure determination

Before crystallization, GSTO1 was dialysed against 25 mM Tris 7.5, 60 mM NaCl and 1 mM DTT. The GSTO1–**C1-27** complex was created by incubating GSTO1 (27 mg ml^−1^) in a 1:1.5 molar ratio with **C1-27** at 4 °C for 48 h. The complex crystallized under sitting drop vapour diffusion against 24% PEG 3350 and 100 mM MES (pH 6.5) with a drop configuration of 2 μl of complex, 1.8 μl well and 0.2 μl 40% tert-butanol. The GSTO1–**C1-31** complex was formed by incubating GSTO1 (2.66 mg ml^−1^) in a 1:1.5 molar ratio with **C1-31** at 4 °C for 24 h, then concentrated to 23.9 mg ml^−1^ before crystallization. Crystals formed from drops containing equal volumes of complex and well solution (22.5% PEG 3350, 90 mM MES (pH 6.5) and 4% tert-butanol). For the GSTO1–**C4-10** complex, GSTO1 (2.66 mg ml^−1^) was incubated in a 1:1.5 molar ratio with **C4-10** for 24 h at 4 °C then concentrated to 26.2 mg ml^−1^. Crystals of the complex grew from sitting drops containing equal volumes of protein complex and well solution (22.5% PEG 3350, 90 mM MES (pH 6.5) and 10 mM BaCl_2_). All crystals grew at 20 °C. The GSTO1–**C1-27** and GSTO1–**C1-31** crystals were cryoprotected with 30% PEG and 100 mM MES (pH 6.5) before data collection; GSTO1–**C4-10** crystals had no additional cryoprotection.

Diffraction data for GSTO1–**C1-27**, GSTO1–**C1-31** and GSTO1–**C4-10** were collected on LS-CAT lines (21-ID-D and 21-ID-G) at the Advanced Photon Source at Argonne National Laboratory and processed with HKL2000 (ref. 46). The structures were solved by molecular replacement (MOLREP^47^) using GSTO1 (PDB ID: 1EEM) as a search model. Three monomers of GSTO1 were present in the asymmetric unit of the GSTO1–**C1-27** and GSTO1–**C4-10** crystals, while a dimer was present in the GSTO1–**C1-31** crystal. Iterative rounds of electron density fitting and refinement were completed using Coot^48^ and Buster^49^, respectively. Difference electron density maps contoured to 3*σ* showed the presence of inhibitor molecules covalently bound to C32 in all protein chains. The coordinates and geometric restraints for each inhibitor were created from smiles using Grade^49^ with the qm+mogul option. Statistics for the refined structures are given in Supplementary Table 27. Residues 1–4 were disordered in all structures along with loop residues 133–135 in **C1-31**. The structures were validated using Whatcheck^50^, Molprobity^51^ and Pavarti^52^.

### GSTO1 enzyme activity assay

Enzyme activity was measured by monitoring the reduction of 4-NPG to 4-nitroacetophenone by GSTO1. Briefly, in a 200 μl reaction volume, 5 μg ml^−1^ recombinant GSTO1 in reaction buffer (100 mM Tris (pH 8.0), 1.5 mM EDTA and 1 mM DTT) was incubated with DMSO or different concentrations of inhibitors for 30 min at 37 °C. A volume of 4-NPG (final concentration of 1 mM) was added to the reaction and decrease in absorbance at 305 nm was recorded on an Envision multilabel plate reader (Perkin Elmer).

### In-gel fluorescence binding assay

Primary screening of GSTO inhibitors was based on competitive inhibition of CMFDA binding to endogenous GSTO. Briefly, HCT116^p53+/+^ cells (4 × 10^4^ per well) were coated in a 12-well plate. After overnight attachment, cells were treated with test compounds at 10 μM for 2 h at 37 °C followed by addition of 500 nM CMFDA for 1 h. Cells were then washed with PBS and lysed using Cell Lytic M buffer (Sigma). A unit of 15 μg of whole-cell protein extracted was boiled with Laemmli sample buffer and resolved on a 15% polyacrylamide gel. Gels were immediately scanned on a Typhoon variable mode imager (Amersham Bioscience). Quantification of fluorescent band intensity was done using Image Quant 5.2 software. Compounds that showed at least 50% inhibition of CMFDA binding to GSTO were selected for dose–response determinations. Similarly, *in vitro* binding assay with recombinant GSTO1 was performed using 1 μM GSTO1 in reaction buffer (100 mM Tris (pH 8.0), 1.5 mM EDTA and 1 μM DTT) incubated with compounds for 30 min at 37 °C and with CMFDA (500 nM) for an additional 30 min. For selectivity studies, recombinant GSTO1 (1 μM), PDI (1 μM), BSA (1 μM) or GRP78 (1 μM) in reaction buffer were incubated with **C1-27** or DMSO for 30 min at 37 °C, followed by incubation with CMFDA (500 nM), BODIPY-**C1-27A** (1 μM) or DMSO for 30 min. Similarly, CRM 13–180 PDX tumour tissue from mice treated with vehicle or **C1-27** was homogenized in Cell lytic M buffer and 20 μg of protein was incubated with CMFDA (500 nM) for 1 h. The reactions were quenched by boiling with Laemmli sample buffer for 5 min. The samples were resolved on a 10–15% polyacrylamide gel and scanned on a Typhoon variable mode imager or on a FluorChem M System (ProteinSimple, San Jose, CA, USA). Uncropped images of key gels are presented in Supplementary Fig. 24.

### Pre-incubation and dilution assay

GSTO1 (2 μg) was incubated with inhibitor at a concentration of 10 × IC_50_ in a 10 μl reaction volume for 30 min at 37 °C. An aliquot of 5 μl of this reaction was diluted to 180 μl with reaction buffer (100 mM Tris (pH 8.0), 1.5 mM EDTA, 1 mM DTT) to attain final concentrations of 5 μg ml^−1^ GSTO1 and 0.25 × IC_50_ of inhibitor. 4-NPG (20 μl, 0.5 mM) was added and decrease in absorbance was monitored. % Recovery of enzyme activity was determined using a DMSO control.

### Duration of inhibition

Receptor occupancy and duration of inhibition experiment was performed based on a previously reported method^53^. HCT116 cells were treated with **C1-27** (100 nM) for 2 h, followed by drug washout. At indicated times after washout, CMFDA (500 nM) was added for 1 h and the cells were lysed and processed for in-gel fluorescence binding assay, to assess recovery of CMFDA binding to GSTO1.

### PDI activity assay

Recombinant PDI was purified and PDI-catalysed reduction of insulin was measured as described previously^54^. Briefly, recombinant PDI protein (0.4 μM) was incubated with indicated compounds at 37 °C for 1 h in sodium phosphate buffer (100 mM sodium phosphate, 2 mM EDTA and 8 μM DTT, pH 7.0). Then the reaction mixture consisting of DTT (500 μM) and bovine insulin (130 μM) was added to the incubated PDI protein. The reduction reaction was catalysed by PDI at room temperature, and the resulting aggregation of reduced insulin B chains was measured at 620 nm. % PDI activity was calculated from absorbance values at *T*=0 and 80 min.

### Thermal-shift assay

Thermal-shift assay was performed as described previously^55^. Briefly, recombinant GSTO1 was diluted into 50 mM Tris-HCl (pH 8.0) buffer containing the fluorophore 1,8 ANS (0.3 mM) to a final concentration of 0.3 mg ml^−1^. A volume of 5 μl of the protein-dye mixture was dispensed into a 384-well microplate (Thermo Scientific, AB1384K), followed by addition of 5 μl of **C1-27** and 3 μl of silicone oil (to avoid evaporation). Similarly, recombinant PDI was diluted to 0.3 mg ml^−1^ in 100 mM sodium phosphate buffer (pH 7.0) containing 0.3 mM 1,8 ANS and thermal-shift assay was performed as described above. A volume of 5 μl of 1.5% v/v DMSO and 2% v/v DMSO in respective buffers for GSTO1 and PDI were used as DMSO controls. Fluorescence intensity as a function of temperature was measured on a Thermofluor analyser (Johnson & Johnson, New Brunswick, NJ). The reaction plate was heated from 25 to 90 °C with a 1 °C min^−1^ increment. Triplicate measurements were made and the s.d. was within limit.

### Cellular thermal-shift assay

Cellular thermal-shift assay was performed as described previously^56^. Briefly, HCT116^p53+/+^ cells were collected, washed with PBS and diluted with cell lysis buffer (25 mM Tris-HCl (pH 7.5) and 10 mM MgCl_2_) supplemented with complete protease inhibitor cocktail. The cell suspension was frozen and thawed three times in liquid nitrogen. The soluble fraction was separated from debris by spinning down at 20,000*g* for 20 min. Cell lysate was then diluted with lysate buffer and divided into two aliquots—one treated with **C1-27** (100 μM) and the other treated with 1% DMSO. After 30 min incubation at room temperature, the treated lysates were further divided into smaller aliquots (50 μl), which were then heated at different temperatures for 3 min on a Veriti thermal cycler (Applied Biosystems) followed by cooling for 3 min to room temperature. The heated lysates were centrifuged at 20,000*g* for 20 min at 4°C to separate the soluble fractions from precipitates. Supernatants were quantified and analysed by western blotting.

### Cell viability assays

Cell proliferation was assessed by a MTT assay. Cancer cells were seeded in 96-well microtitre plates and, after overnight attachment, treated with GSTO1 inhibitors. After 72 h, MTT solution (3 mg ml^−1^; 20 μl) was added to each well and cells were incubated for 3 h at 37 °C. After incubation, media from each well was removed and the dark blue formazan crystals formed by live cells were dissolved in DMSO (150 μl per well). The absorbance intensity was measured at 570 nm on a microplate reader (Molecular Devices, Sunnyvale, CA, USA). Cell viability after 24 h treatment was assessed using ApoTox-Glo triplex assay (Promega) according to the manufacturer's protocol. At least three independent dose–response experiments with each concentration tested in triplicate were performed for each cell line.

### Clonogenic survival assay

HCT116 cells were seeded at a density of 200 cells per well in a 6-well microtitre plate. After overnight attachment, cells were treated with different concentrations of GSTO1 inhibitors for 24 h. Colonies were allowed to grow in drug-free medium for 7 days and stained with 0.5% crystal violet. For drug combination studies, HCT116 cells (200 per well) were plated in a 12-well plate. **C1-27**, cisplatin (1 μM, dissolved in 0.9% NaCl), PD98059 (10 μM), LY294002 (10 μM), SP600125 (1 μM) or desferoxamine mesylate (10 μM) were added alone or in combination and colonies were allowed to grow for 7–10 days.

### Cell cycle analysis

Cells were seeded at a density of 0.5 × 10^6^ per well in a six-well plate. After overnight attachment, cells were treated with **C1-27** (5 μM) for 24 h and collected using trypsin. Cells were then washed twice with PBS containing 10% serum and fixed with 70% ethanol overnight at −20 °C. Fixed cells were washed twice in PBS containing 10% serum and stained with 50 μg ml^−1^ propidium iodide solution containing 100 μg ml^−1^ RNase A for 1 h at 37 °C. Cell cycle was analysed on a BD LSR II flow cytometer (BD Biosciences) and data were analysed using ModFit software.

### Caspase 3/7 assay

HCT116 cells were treated with indicated concentrations of **C1-27** for 24 h. Caspase activation was measured using ApoTox-Glo triplex assay reagent according to the manufacturer's recommendation.

### Western blotting

HCT116 cells were seeded in a six-well plate and treated with GSTO1 inhibitors for 24 h. Cells were washed with ice-cold PBS and lysed with a triple detergent lysis buffer supplemented with protease and phosphatase inhibitor cocktail. Lysate was sonicated and centrifuged at 12,000 r.p.m. for 10 min at 4 °C to remove cell debris. Total protein concentration in the supernatant was measured using BCA protein assay kit (Pierce Biotechnology). For IL-1β experiments, THP-1 cells (1 × 10^6^ per well) were seeded in a 12-well plate and differentiated overnight with 10 nM phorbol myristate acetate. Attached THP-1 cells were stimulated with 100 ng ml^−1^ lipopolysaccharide (Sigma) for 3 h and treated with DMSO, GSTO1 inhibitors or Glyburide (Enzo Life sciences) in serum-free media for 1.5 h followed by addition of 5 mM ATP (Sigma) for 30 min. Supernatant media was collected and precipitated using trichloroacetic acid. Cell fraction was collected and processed as described above. A unit of 40 μg of protein lysate was incubated with SDS sample buffer for 5 min at 90 °C, resolved on a 10% polyacrylamide gel and electroblotted on a polyvinylidene difluoride membrane. Membranes were blocked in 5% non-fat milk in Tris-buffered saline with 0.1% Tween-20 (TBST) for 1 h at room temperature and incubated overnight with primary antibodies at dilutions specified in Supplementary Table 26. Subsequently, the membranes were washed with TBST and incubated with appropriate horseradish peroxidase-linked secondary antibodies for 2 h at room temperature followed by further washing with TBST. The immunoblots were visualized using ECL Western blotting substrate (Pierce Biotechnology) on a Chemidoc XRS imager (Bio Rad) or using Dylight 800-conjugated secondary antibodies (Thermo Fisher Scientific, Rockford, IL) on an Odyssey Imaging Systems (LI-COR Biosciences, Lincoln, NE). Band intensities were quantified using Image J software. Uncropped images of key western blots are presented in Supplementary Figs 25 and 26.

### Bru-seq nascent RNA profiling

HCT116 cells (4 × 10^6^) were treated with siGSTO1 (10 nM), DMSO or **C1-14** (10 μM) for 4 h or with **C1-27** (1 μM) for 4 and 24 h. Nascent RNA was labelled, isolated and processed according to previously published protocol^27^,^57^.

### Pathway analysis

GSTO1 Bru-Seq data set was filtered using the cutoff value of gene size >300 bp and mean reads per kilobase per million mapped reads (RPKM)>0.5, and a total of 8,072 genes in **C1-27** 4 h treatment, 8,324 genes in **C1-27** 24 h treatment, 8,172 genes in **C1-14** 4 h treatment and 8,962 genes in GSTO1 siRNA treatment groups were ranked based on fold change values versus control (DMSO) (Supplementary Data Set 1). Shown in Supplementary Tables 10 and 11 and Supplementary Tables 14–19 are top 20 up- and downregulated genes in response to siRNA and drug treatments. The data set was analysed using the DAVID, ingenuity pathway analysis (IPA) and GSEA for pathway identification. DAVID (http://david.abcc.ncifcrf.gov/home.jsp) functional annotation analysis^28^,^58^ was performed on the list of up- and downregulated genes with a fold change ≥1.4. Only those terms that reported a *P* value of ≤0.05 and count number ≥4 genes were selected for analysis. Pathway analysis of siGSTO1 Bru-Seq data was also performed using IPA (Ingenuity Systems, Inc., Redwood City, CA) with genes showing fold change ≥2. Data sets with nearly 250 genes containing the gene identifiers and fold changes were uploaded to the IPA web-based application and each gene identifier was mapped to its corresponding gene object in the ingenuity pathways knowledge base. Finally, the biological function genes were ordered by *P* value of significance and maximum number of genes in the pathway. Bru-Seq data were further analysed using GSEA for exploring the mechanism of cellular action of GSTO1 and its inhibitor. A pre-ranked gene list of the Bru-Seq data set was submitted to GSEA, and enrichment analysis was done based on the Kolmogorov–Smirnov statistic^59^,^60^. GSEA is a knowledge-based approach for interpreting genomic profiles based on the gene sets. For each gene set, the ES (enrichment score) was normalized to account for differences in gene set size and the false discovery rate relative to the normalized enrichment score values was calculated. Supplementary Tables 12 and 13 and Supplementary Tables 20–24 show the top GSEA gene sets for the up- and downregulated genes in siGSTO1 and drug treatment Bru-Seq data sets.

### *In vivo* efficacy studies

In the pilot study, HCT116 cells (1 × 10^6^) in exponential phase were injected subcutaneouslyinto the left flank of 8- to 10-week-old female nude mice (25–30 g; Simonsen laboratories, Gilroy, CA). The perpendicular diameters of the tumours were measured three times weekly using standard calipers and tumour volumes were calculated using the formula: 0.5 × *D* × *d*^2^, where *D* and *d* were the longest and shortest perpendicular diameters, respectively. Tumours were allowed to grow to a volume of 50 mm^3^ and mice were randomized into control (*n*=5) and **C1-27** (*n*=3) treatment groups. **C1-27** dissolved in peanut oil was administered intraperitoneally (20 mg kg^−1^ per day) for the first 2 weeks on a 5 days on/2 days off schedule. The dose was then increased to 25 mg kg^−1^ per day for the next 23 days and further escalated by 5 mg kg^−1^ per day to a final dose of 45 mg kg^−1^ for the remaining duration of treatment. Tumour volumes and body weights were measured three times weekly to monitor tumour burden and weight loss during treatment. At the end of the experiment, animals were killed and tumour, kidney and liver were collected, fixed and paraffin-embedded for histology. The animal experiments were done in accordance with protocols approved by the University of Southern California Institutional Animal Care and Use Committee.

For the colorectal cancer PDX study, female NCR nude mice (CrTac:NCr-*Foxn1*^*nu*^ from Taconic) were implanted subcutaneously with low-passage KRAS-mutant CRM 13–180 tumour fragments (∼30 mg) into the region of the right axilla. The CRM 13–180 PDX model was provided by the laboratory of Dr Judith Leopold (University of Michigan)^61^. Mice were randomized into treatment groups (*n*=5) and treatments initiated when tumours reached ∼100 mg. **C1-27**, SC-144 and As_2_O_3_ were administered intraperitoneally in a fixed volume of 100 μl, on a 5 days on/2 days off schedule. L-OHP was administered intraperitoneally in a fixed volume of 100 μl once a week. Subcutaneous tumour volume and body weights were measured three times a week. Tumour volumes were calculated by measuring two perpendicular diameters with calipers and using the formula: tumour volume=(length × width^2^)/2. Mice were treated as indicated until the mean tumour burden in the vehicle control group reached ∼1,000 mg. Mice received a total of four cycles of treatment. %*T*/*C* was calculated by dividing the median treated tumour weight by the median control tumour weight and multiplying by 100 on the last day of treatment. Tumour growth delay (*T*−*C*) was calculated by subtracting the median time to reach evaluation size (750 mg) of the treated group by the median time to evaluation size of the control group. All procedures related to the handling, care and treatment of animals were conducted in accordance with University of Michigan's Committee on the Use and Care of Animals guidelines. The investigators were not blinded in either study.

### Statistical analysis

Data are presented as mean of at least three independent experiments and error bars denote s.e.m. or s.d. as indicated. Statistical analysis was done using Prism 7.0 (Graphpad software). Single comparisons were performed by two-sided Student's *t*-test assuming unequal variance. Multiple comparisons were analysed using one-way analysis of variance or two-way analysis of variance followed by Dunnett's *post hoc* test. *P* values at *α*=0.05 are reported (*P<0.05, **P<0.01, ***P<0.001 and ****P<0.0001). For the PDX efficacy study, a sample size of five animals per treatment group was estimated to provide more than 80% power using two-sided *P*<0.05 criterion.

### Data availability

The coordinates for GSTO1 inhibitor complexes have been deposited with the PDB: **C1-27** (4YQM); **C1-31** (4YQU) and **C4-10** (4YQV). The Bru-Seq data discussed in this publication have been deposited in the NCBI Gene Expression Omnibus and are accessible through the GEO series accession number GSE85899. All relevant data that support the findings of this study are available within the article and its Supplementary Information, or from the corresponding author on request.

## Additional information

**How to cite this article:** Ramkumar, K. *et al*. Mechanistic evaluation and transcriptional signature of a glutathione *S*-transferase omega 1 inhibitor. *Nat. Commun.* **7,** 13084 doi: 10.1038/ncomms13084 (2016).

## Supplementary Material

Supplementary Information Supplementary Figures 1 - 26, Supplementary Tables 1 - 27, Supplementary Methods 1 - 2 and Supplementary References

## References

[b1] Hayes J. D., Flanagan J. U. & Jowsey I. R. Glutathione transferases. Annu. Rev. Pharmacol. Toxicol. 45, 51–88 (2005). 15822171 10.1146/annurev.pharmtox.45.120403.095857

[b2] Board P. G. & Menon D. Glutathione transferases, regulators of cellular metabolism and physiology. Biochim. Biophys. Acta 1830, 3267–3288 (2013). 23201197 10.1016/j.bbagen.2012.11.019

[b3] McIlwain C. C., Townsend D. M. & Tew K. D. Glutathione S-transferase polymorphisms: cancer incidence and therapy. Oncogene 25, 1639–1648 (2006). 16550164 10.1038/sj.onc.1209373 PMC6361140

[b4] Laborde E. Glutathione transferases as mediators of signaling pathways involved in cell proliferation and cell death. Cell Death Differ. 17, 1373–1380 (2010). 20596078 10.1038/cdd.2010.80

[b5] Board P. G. . Identification, characterization, and crystal structure of the Omega class glutathione transferases. J. Biol. Chem. 275, 24798–24806 (2000). 10783391 10.1074/jbc.M001706200

[b6] Zhou H., Brock J., Liu D., Board P. G. & Oakley A. J. Structural insights into the dehydroascorbate reductase activity of human omega-class glutathione transferases. J. Mol. Biol. 420, 190–203 (2012). 22522127 10.1016/j.jmb.2012.04.014

[b7] Board P. G. The omega-class glutathione transferases: structure, function, and genetics. Drug Metab. Rev. 43, 226–235 (2011). 21495794 10.3109/03602532.2011.561353

[b8] Burmeister C. . Oxidative stress in *Caenorhabditis elegans*: protective effects of the Omega class glutathione transferase (GSTO-1). FASEB J. 22, 343–354 (2008). 17901115 10.1096/fj.06-7426com

[b9] Kim K., Kim S. H., Kim J., Kim H. & Yim J. Glutathione S-transferase omega 1 activity is sufficient to suppress neurodegeneration in a *Drosophila* model of Parkinson's disease. J. Biol. Chem. 287, 6628–6641 (2012). 22219196 10.1074/jbc.M111.291179 PMC3307323

[b10] Kodym R., Calkins P. & Story M. The cloning and characterization of a new stress response protein. A mammalian member of a family of theta class glutathione S-transferase-like proteins. J. Biol. Chem. 274, 5131–5137 (1999). 9988762 10.1074/jbc.274.8.5131

[b11] Menon D. & Board P. G. A role for glutathione transferase Omega 1 (GSTO1-1) in the glutathionylation cycle. J. Biol. Chem. 288, 25769–25779 (2013). 23888047 10.1074/jbc.M113.487785 PMC3764784

[b12] Laliberte R. E. . Glutathione s-transferase omega 1-1 is a target of cytokine release inhibitory drugs and may be responsible for their effect on interleukin-1beta posttranslational processing. J. Biol. Chem. 278, 16567–16578 (2003). 12624100 10.1074/jbc.M211596200

[b13] Coll R. C. & O'Neill L. A. The cytokine release inhibitory drug CRID3 targets ASC oligomerisation in the NLRP3 and AIM2 inflammasomes. PLoS ONE 6, e29539 (2011). 22216309 10.1371/journal.pone.0029539 PMC3245271

[b14] Menon D., Coll R., O'Neill L. A. & Board P. G. Glutathione transferase omega 1 is required for the lipopolysaccharide-stimulated induction of NADPH oxidase 1 and the production of reactive oxygen species in macrophages. Free Radic. Biol. Med. 73, 318–327 (2014). 24873723 10.1016/j.freeradbiomed.2014.05.020

[b15] Paul S., Jakhar R., Bhardwaj M. & Kang S. C. Glutathione-S-transferase omega 1 (GSTO1-1) acts as mediator of signaling pathways involved in aflatoxin B1-induced apoptosis-autophagy crosstalk in macrophages. Free Radic. Biol. Med. 89, 1218–1230 (2015). 26561775 10.1016/j.freeradbiomed.2015.11.006

[b16] Adam G. C., Sorensen E. J. & Cravatt B. F. Proteomic profiling of mechanistically distinct enzyme classes using a common chemotype. Nat. Biotechnol. 20, 805–809 (2002). 12091914 10.1038/nbt714

[b17] Board P. G. . S-(4-Nitrophenacyl)glutathione is a specific substrate for glutathione transferase omega 1-1. Anal. Biochem. 374, 25–30 (2008). 18028863 10.1016/j.ab.2007.09.029

[b18] Yan X. D., Pan L. Y., Yuan Y., Lang J. H. & Mao N. Identification of platinum-resistance associated proteins through proteomic analysis of human ovarian cancer cells and their platinum-resistant sublines. J. Proteome Res. 6, 772–780 (2007). 17269733 10.1021/pr060402r

[b19] Piaggi S. . Glutathione transferase omega 1-1 (GSTO1-1) plays an anti-apoptotic role in cell resistance to cisplatin toxicity. Carcinogenesis 31, 804–811 (2010). 20106899 10.1093/carcin/bgq031

[b20] Bachovchin D. A., Brown S. J., Rosen H. & Cravatt B. F. Identification of selective inhibitors of uncharacterized enzymes by high-throughput screening with fluorescent activity-based probes. Nat. Biotechnol. 27, 387–394 (2009). 19329999 10.1038/nbt.1531 PMC2709489

[b21] Tsuboi K. . Potent and selective inhibitors of glutathione S-transferase omega 1 that impair cancer drug resistance. J. Am. Chem. Soc. 133, 16605–16616 (2011). 21899313 10.1021/ja2066972 PMC3226709

[b22] Son J., Lee J. J., Lee J. S., Schuller A. & Chang Y. T. Isozyme-specific fluorescent inhibitor of glutathione S-transferase omega 1. ACS Chem. Biol. 5, 449–453 (2010). 20205472 10.1021/cb100007s

[b23] Pace N. J., Pimental D. R. & Weerapana E. An inhibitor of glutathione S-transferase omega 1 that selectively targets apoptotic cells. Angew. Chem. Int. Ed. 51, 8365–8368 (2012). 10.1002/anie.201203730 22777685

[b24] Sampayo-Reyes A. & Zakharyan R. A. Inhibition of human glutathione S-transferase omega by tocopherol succinate. Biomed. Pharmacother. 60, 238–244 (2006). 16781109 10.1016/j.biopha.2006.04.005

[b25] Hoffstrom B. G. . Inhibitors of protein disulfide isomerase suppress apoptosis induced by misfolded proteins. Nat. Chem. Biol. 6, 900–906 (2010). 21079601 10.1038/nchembio.467 PMC3018711

[b26] Brock J., Board P. G. & Oakley A. J. Structural insights into omega-class glutathione transferases: a snapshot of enzyme reduction and identification of a non-catalytic ligandin site. PLoS ONE 8, e60324 (2013). 23593192 10.1371/journal.pone.0060324 PMC3621891

[b27] Paulsen M. T. . Coordinated regulation of synthesis and stability of RNA during the acute TNF-induced proinflammatory response. Proc. Natl Acad. Sci. USA 110, 2240–2245 (2013). 23345452 10.1073/pnas.1219192110 PMC3568384

[b28] Dennis G. Jr . DAVID: database for annotation, visualization, and integrated discovery. Genome Biol. 4, P3 (2003). 12734009 PMC193660

[b29] Krige D. . CHR-2797: an antiproliferative aminopeptidase inhibitor that leads to amino acid deprivation in human leukemic cells. Cancer Res. 68, 6669–6679 (2008). 18701491 10.1158/0008-5472.CAN-07-6627

[b30] Chang H. Y. . Gene expression signature of fibroblast serum response predicts human cancer progression: similarities between tumors and wounds. PLoS Biol. 2, E7 (2004). 14737219 10.1371/journal.pbio.0020007 PMC314300

[b31] Malhotra D. . Global mapping of binding sites for Nrf2 identifies novel targets in cell survival response through ChIP-Seq profiling and network analysis. Nucleic Acids Res. 38, 5718–5734 (2010). 20460467 10.1093/nar/gkq212 PMC2943601

[b32] Jost C., Nitsche C., Scholz T., Roux L. & Klein C. D. Promiscuity and selectivity in covalent enzyme inhibition: a systematic study of electrophilic fragments. J. Med. Chem. 57, 7590–7599 (2014). 25148591 10.1021/jm5006918

[b33] Zhou H., Brock J., Casarotto M. G., Oakley A. J. & Board P. G. Novel folding and stability defects cause a deficiency of human glutathione transferase omega 1. J. Biol. Chem. 286, 4271–4279 (2011). 21106529 10.1074/jbc.M110.197822 PMC3039386

[b34] Weiwer M. . Development of small-molecule probes that selectively kill cells induced to express mutant RAS. Bioorg. Med. Chem. Lett. 22, 1822–1826 (2012). 22297109 10.1016/j.bmcl.2011.09.047 PMC3528973

[b35] Yang W. S. & Stockwell B. R. Synthetic lethal screening identifies compounds activating iron-dependent, nonapoptotic cell death in oncogenic-RAS-harboring cancer cells. Chem. Biol. 15, 234–245 (2008). 18355723 10.1016/j.chembiol.2008.02.010 PMC2683762

[b36] Yang W. S. . Regulation of ferroptotic cancer cell death by GPX4. Cell 156, 317–331 (2014). 24439385 10.1016/j.cell.2013.12.010 PMC4076414

[b37] Raj L. . Selective killing of cancer cells by a small molecule targeting the stress response to ROS. Nature 475, 231–234 (2011). 21753854 10.1038/nature10167 PMC3316487

[b38] Adams D. J. . Synthesis, cellular evaluation, and mechanism of action of piperlongumine analogs. Proc. Natl Acad. Sci. USA 109, 15115–15120 (2012). 22949699 10.1073/pnas.1212802109 PMC3458345

[b39] Kumar A. . Mammalian proapoptotic factor ChaC1 and its homologues function as gamma-glutamyl cyclotransferases acting specifically on glutathione. EMBO Rep. 13, 1095–1101 (2012). 23070364 10.1038/embor.2012.156 PMC3512401

[b40] Mungrue I. N., Pagnon J., Kohannim O., Gargalovic P. S. & Lusis A. J. CHAC1/MGC4504 is a novel proapoptotic component of the unfolded protein response, downstream of the ATF4-ATF3-CHOP cascade. J. Immunol. 182, 466–476 (2009). 19109178 10.4049/jimmunol.182.1.466 PMC2846782

[b41] Ruiz C. . Growth promoting signaling by tenascin-C [corrected]. Cancer Res. 64, 7377–7385 (2004). 15492259 10.1158/0008-5472.CAN-04-1234

[b42] Zhu S., Mc Henry K. T., Lane W. S. & Fenteany G. A chemical inhibitor reveals the role of Raf kinase inhibitor protein in cell migration. Chem. Biol. 12, 981–991 (2005). 16183022 10.1016/j.chembiol.2005.07.007

[b43] Menon D., Coll R., O'Neill L. A. & Board P. G. GSTO1-1 modulates metabolism in macrophages activated through the LPS and TLR4 pathway. J. Cell Sci. 128, 1982–1990 (2015). 25908843 10.1242/jcs.167858

[b44] Xu S., Grande F., Garofalo A. & Neamati N. Discovery of a novel orally active small-molecule gp130 inhibitor for the treatment of ovarian cancer. Mol. Cancer Ther. 12, 937–949 (2013). 23536726 10.1158/1535-7163.MCT-12-1082

[b45] Whitbread A. K., Tetlow N., Eyre H. J., Sutherland G. R. & Board P. G. Characterization of the human Omega class glutathione transferase genes and associated polymorphisms. Pharmacogenetics 13, 131–144 (2003). 12618591 10.1097/00008571-200303000-00003

[b46] Otwinowski Z. & Minor W. Processing of X-ray diffraction data. Methods Enzymol. 276, 307–326 (1997). 10.1016/S0076-6879(97)76066-X 27754618

[b47] Vagin A. & Teplyakov A. MOLREP: an automated program for molecular replacement. J. Appl. Crystallogr. 30, 1022–1025 (1997).

[b48] Emsley P. & Cowtan K. Coot: model-building tools for molecular graphics. Acta Crystallogr. D Biol. Crystallogr. 60, 2126–2132 (2004). 15572765 10.1107/S0907444904019158

[b49] Bricogne G. . BUSTER version 2.10.2 Cambridge: Global Phasing Ltd, UK (2016).

[b50] Hooft R. W., Vriend G., Sander C. & Abola E. E. Errors in protein structures. Nature 381, 272 (1996). 8692262 10.1038/381272a0

[b51] Davis I. W. . MolProbity: all-atom contacts and structure validation for proteins and nucleic acids. Nucleic Acids Res. 35, W375–W383 (2007). 17452350 10.1093/nar/gkm216 PMC1933162

[b52] Zucker F., Champ P. C. & Merritt E. A. Validation of crystallographic models containing TLS or other descriptions of anisotropy. Acta Crystallogr. D Biol. Crystallogr. 66, 889–900 (2010). 20693688 10.1107/S0907444910020421 PMC2917275

[b53] Serafimova I. M. . Reversible targeting of noncatalytic cysteines with chemically tuned electrophiles. Nat. Chem. Biol. 8, 471–476 (2012). 22466421 10.1038/nchembio.925 PMC3657615

[b54] Xu S. . Discovery of an orally active small-molecule irreversible inhibitor of protein disulfide isomerase for ovarian cancer treatment. Proc. Natl Acad. Sci. USA 109, 16348–16353 (2012). 22988091 10.1073/pnas.1205226109 PMC3479552

[b55] Pantoliano M. W. . High-density miniaturized thermal shift assays as a general strategy for drug discovery. J. Biomol. Screen. 6, 429–440 (2001). 11788061 10.1177/108705710100600609

[b56] Martinez Molina D. . Monitoring drug target engagement in cells and tissues using the cellular thermal shift assay. Science 341, 84–87 (2013). 23828940 10.1126/science.1233606

[b57] Veloso A. . Genome-wide transcriptional effects of the anti-cancer agent camptothecin. PloS ONE 8, e78190 (2013). 24194914 10.1371/journal.pone.0078190 PMC3806802

[b58] Huang, da W., Sherman B. T. & Lempicki R. A. Systematic and integrative analysis of large gene lists using DAVID bioinformatics resources. Nat. Protoc. 4, 44–57 (2009). 19131956 10.1038/nprot.2008.211

[b59] Mootha V. K. . PGC-1alpha-responsive genes involved in oxidative phosphorylation are coordinately downregulated in human diabetes. Nat. Genet. 34, 267–273 (2003). 12808457 10.1038/ng1180

[b60] Subramanian A. . Gene set enrichment analysis: a knowledge-based approach for interpreting genome-wide expression profiles. Proc. Natl Acad. Sci. USA 102, 15545–15550 (2005). 16199517 10.1073/pnas.0506580102 PMC1239896

[b61] Ziemke E. K. . Sensitivity of KRAS mutant colorectal cancers to combination therapy that co-targets MEK and CDK4/6. Clin. Cancer Res. 22, 405–414 (2015). 26369631 10.1158/1078-0432.CCR-15-0829 PMC4715945

