# Supplementary material for: Mechanistic evaluation and transcriptional signature of a glutathione S-transferase omega 1 inhibitor
Source: Nat Commun. 2016 Oct 5;7:13084. doi: 10.1038/ncomms13084 (PMC5059489; doi:10.1038/ncomms13084)
Supplement: Supplementary Information — Supplementary Figures 1 - 26, Supplementary Tables 1 - 27, Supplementary Methods 1 - 2 and Supplementary References [file ncomms13084-s1.pdf]

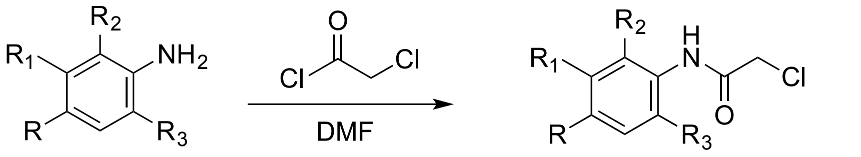

1a-c **2a-c**

a: R, R_2 = F; R_1,R_3 = H
b: R, R_2 = F; R_1 = H; R_3 = NO_2
c: R, R_1 = F; R_2 = H; R_3 = NO_2

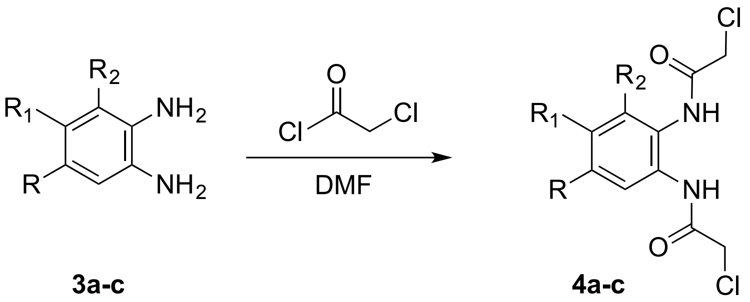

3a-c **4a-c**

4a: R, R_1 = F; R_2 = H
4b: R, R_2 = F; R_1 = H
c: R, R_1, R_2 = H

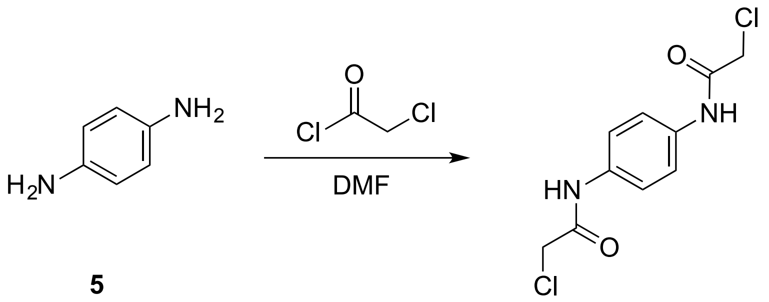

5 **6**

Supplementary Fig. 1. Synthetic scheme of novel small molecules for pilot screening.

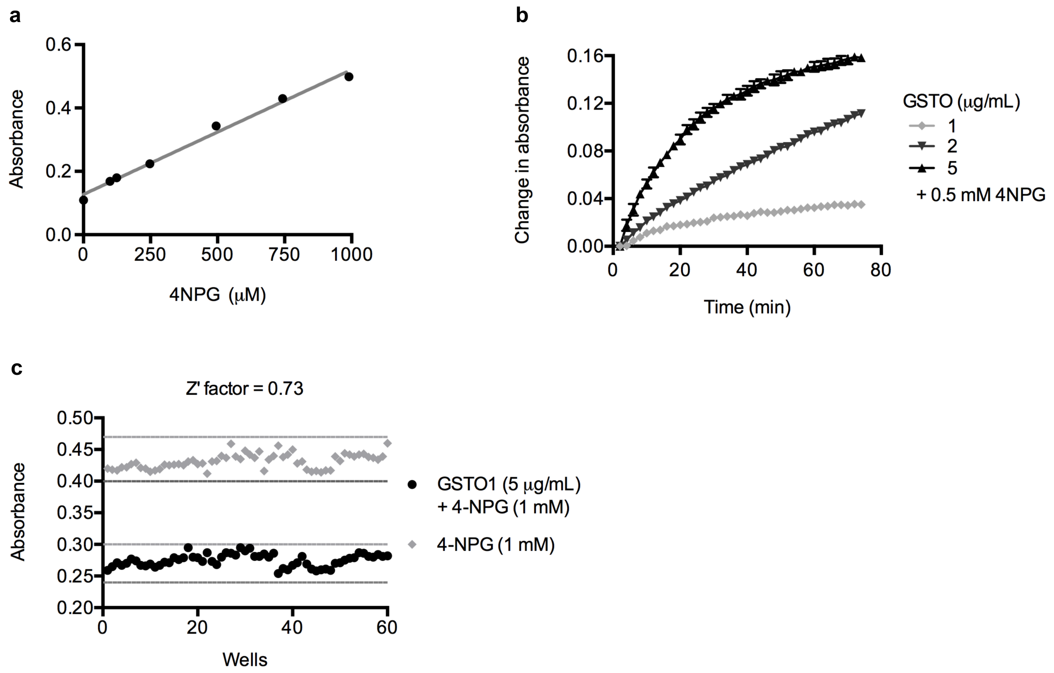

Supplementary Fig. 2. GSTO1 substrate assay optimization. (**a**) Linearity of 4-nitrophenacyl glutathione (4NPG) signal ($R^2 = 0.99$). Data are mean ± SEM of triplicate wells. (**b**) Kinetics of GSTO1-catalyzed reduction of 4-NPG. Data are mean ± SEM of triplicate wells. (**c**) Assay performance characteristics in high throughput format (average Z' factor = 0.73, average S/N ratio = 16.5).

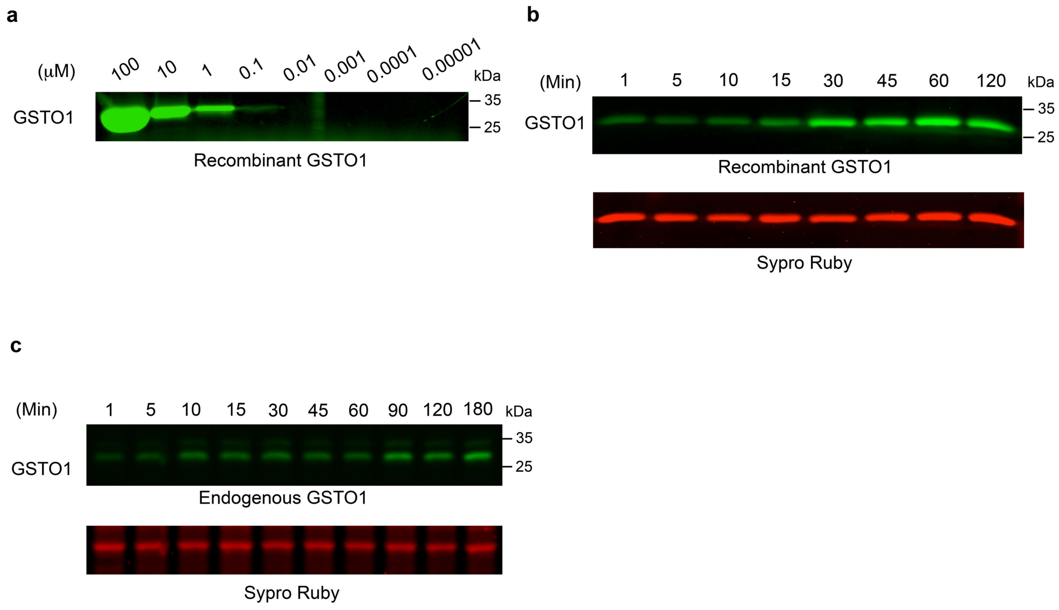

Supplementary Fig. 3. GSTO1 competitive binding assay optimization. (**a**) CMFDA (500 nM) binding to increasing concentrations of recombinant GSTO1. (**b**) Time course for saturation of CMFDA (500 nM) binding to recombinant GSTO1 (1 µM). (**c**) Time course for saturation of CMFDA (500 nM) binding to endogenous GSTO1 (12 µg of soluble proteome).

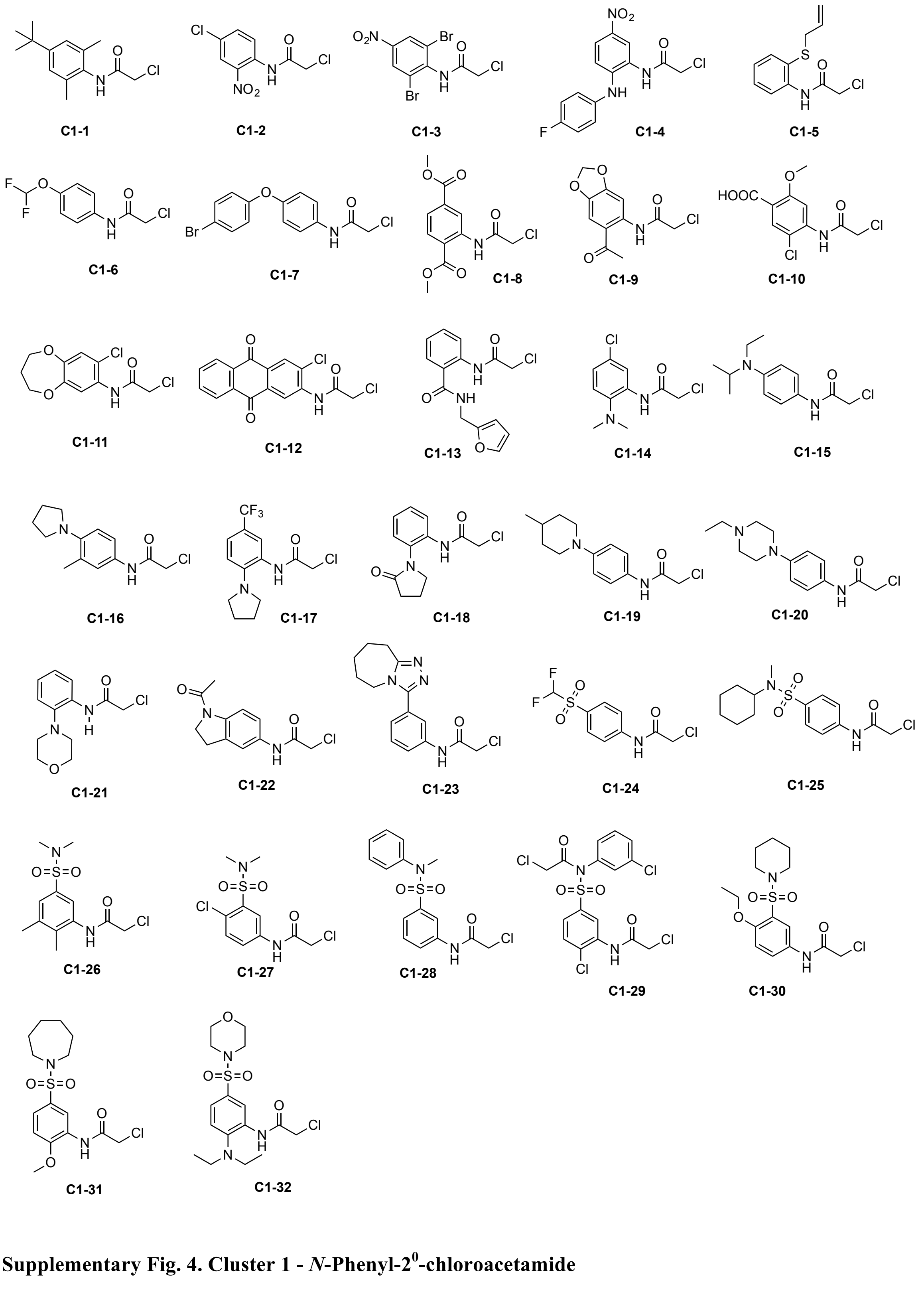

Supplementary Fig. 4. Cluster 1 - *N*-Phenyl-2^{0}-chloroacetamide

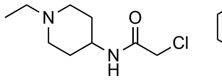

C2-1

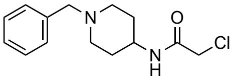

C2-2

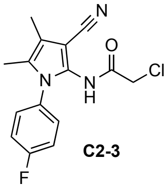

C2-3

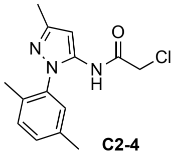

C2-4

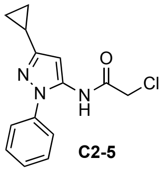

C2-5

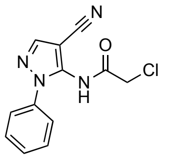

C2-6

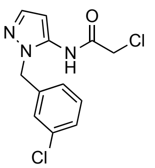

C2-7

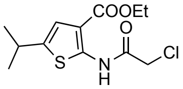

C2-8

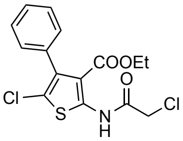

C2-9

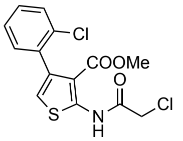

C2-10

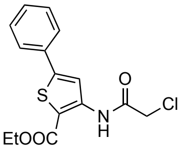

C2-11

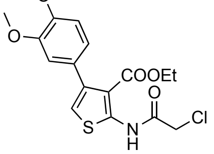

C2-12

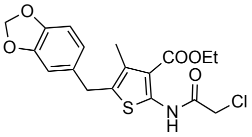

C2-13

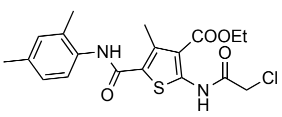

C2-14

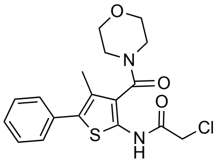

C2-15

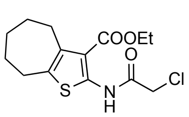

C2-16

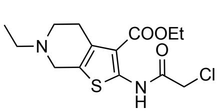

C2-17

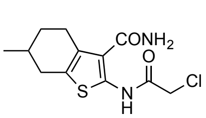

C2-18

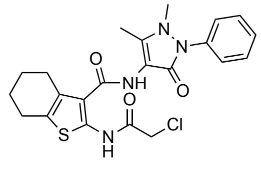

C2-19

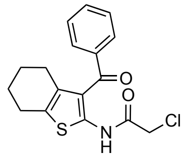

C2-20

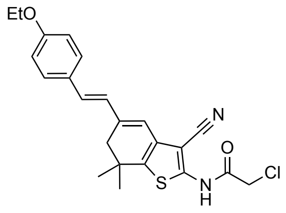

C2-21

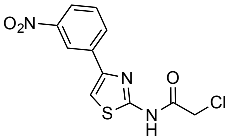

C2-22

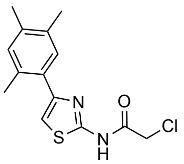

C2-23

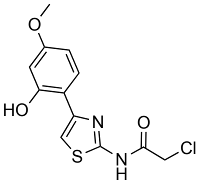

C2-24

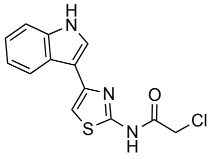

C2-25

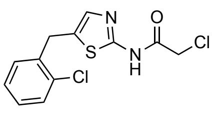

C2-26

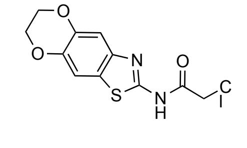

C2-27

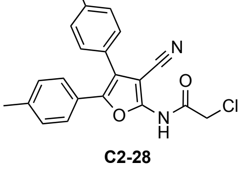

C2-28

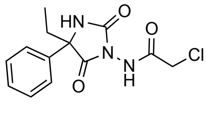

C2-29

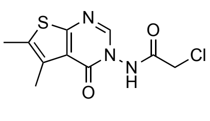

C2-30

Supplementary Fig. 5. Cluster 2 - *N*-Heterocycle-2^0-chloroacetamide

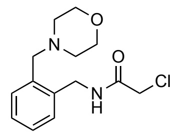

C3-1

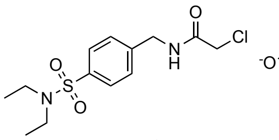

C3-2

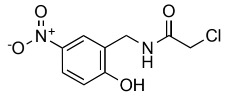

C3-3

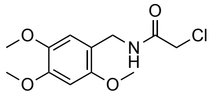

C3-4

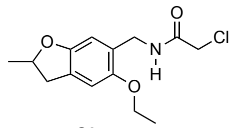

C3-5

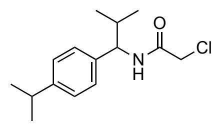

C3-6

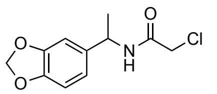

C3-7

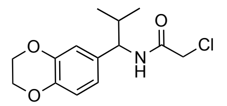

C3-8

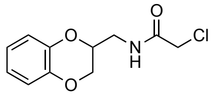

C3-9

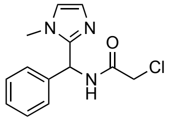

C3-10

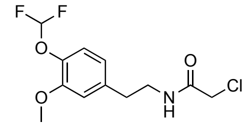

C3-11

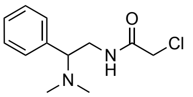

C3-12

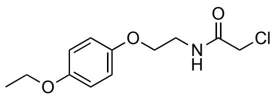

C3-13

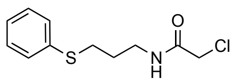

C3-14

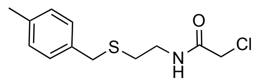

C3-15

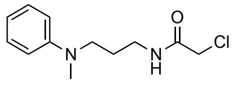

C3-16

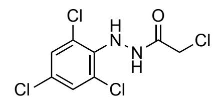

C3-17

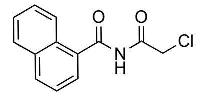

C3-18

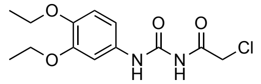

C3-19

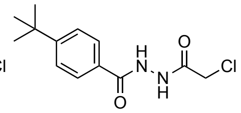

C3-20

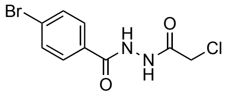

C3-21

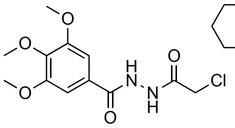

C3-22

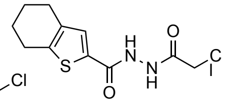

C3-23

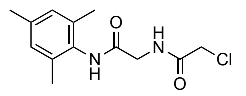

C3-24

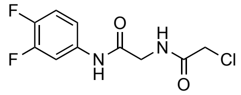

C3-25

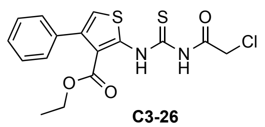

C3-26

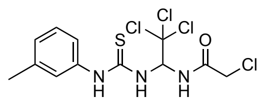

C3-27

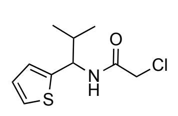

C3-28

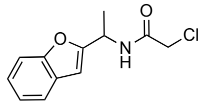

C3-29

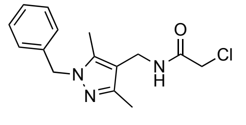

C3-30

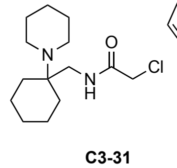

C3-31

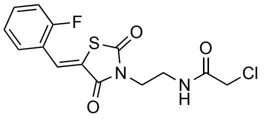

C3-32

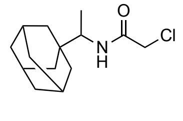

C3-33

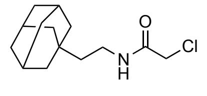

C3-34

Supplementary Fig. 6. Cluster 3 - *N*-Linker-2^0-chloroacetamide

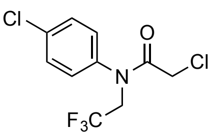

C4-1

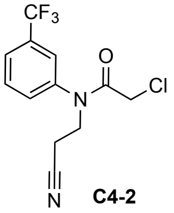

C4-2

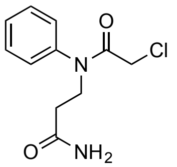

C4-3

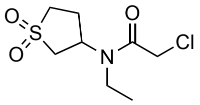

C4-4

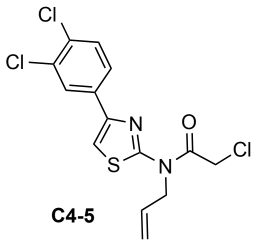

C4-5

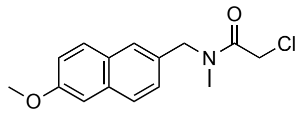

C4-6

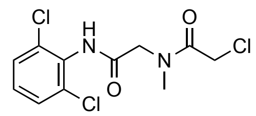

C4-7

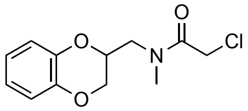

C4-8

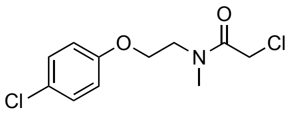

C4-9

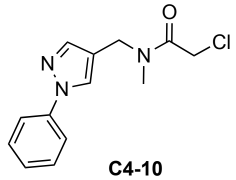

C4-10

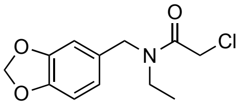

C4-11

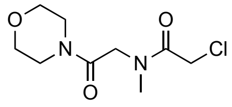

C4-12

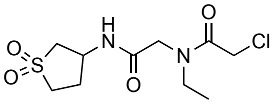

C4-13

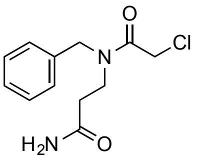

C4-14

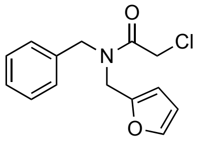

C4-15

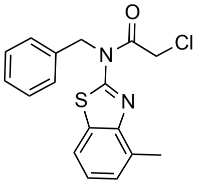

C4-16

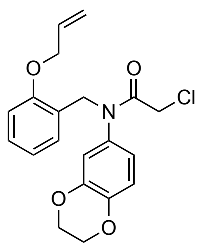

C4-17

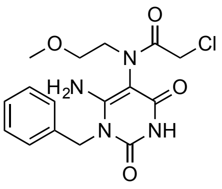

C4-18

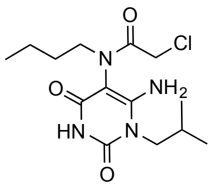

C4-19

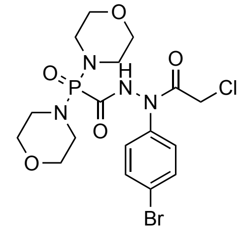

C4-20

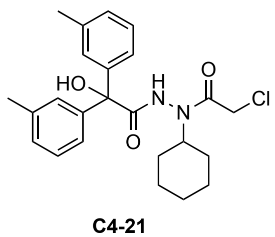

C4-21

Supplementary Fig. 7. Cluster 4 - Non-cyclized 3^0-chloroacetamide

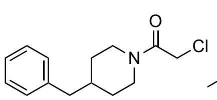
C5-1
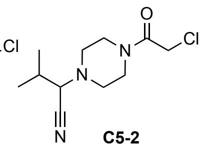
C5-2
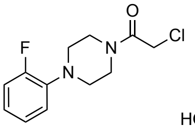
C5-3
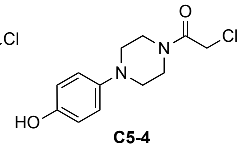
C5-4
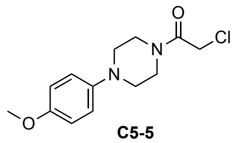
C5-5
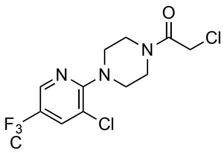
C5-6
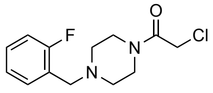
C5-7
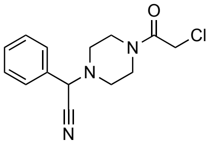
C5-8
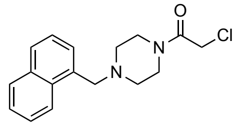
C5-9
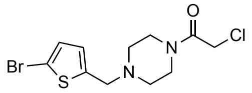
C5-10
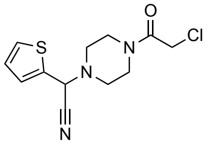
C5-11
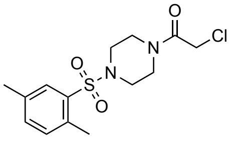
C5-12
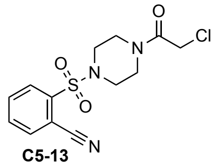
C5-13
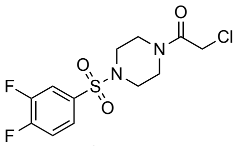
C5-14
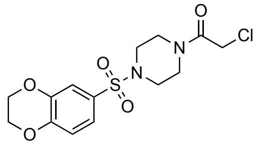
C5-15
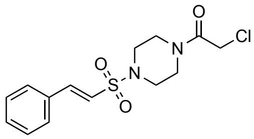
C5-16
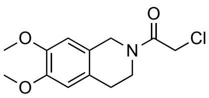
C5-17
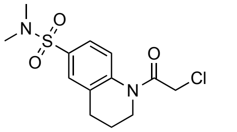
C5-18
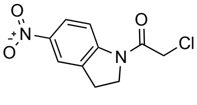
C5-19
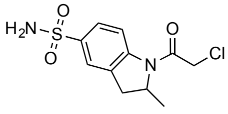
C5-20
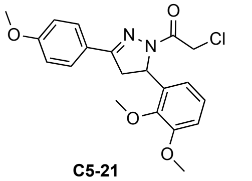
C5-21
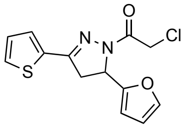
C5-22
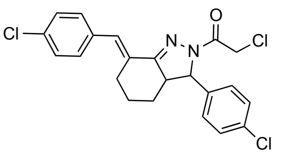
C5-23
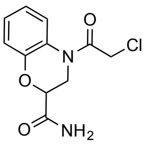
C5-24

Supplementary Fig. 8. Cluster 5 - Cyclized 3^{0}-chloroacetamide

Scheme A. Synthesis of amino-BODIPY dye (**2**)

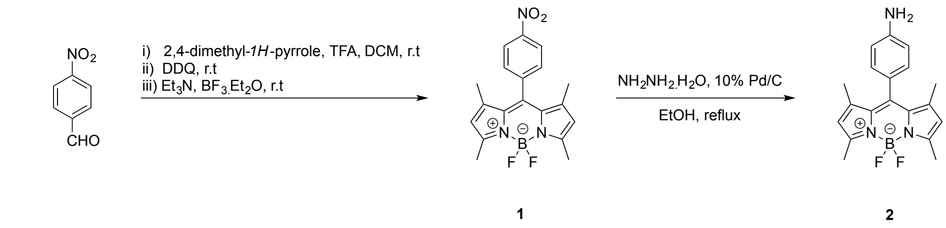

Scheme B. Synthesis of **C1-27A** (**7a**) and BODIPY-conjugated **C1-27A** (**7b**)

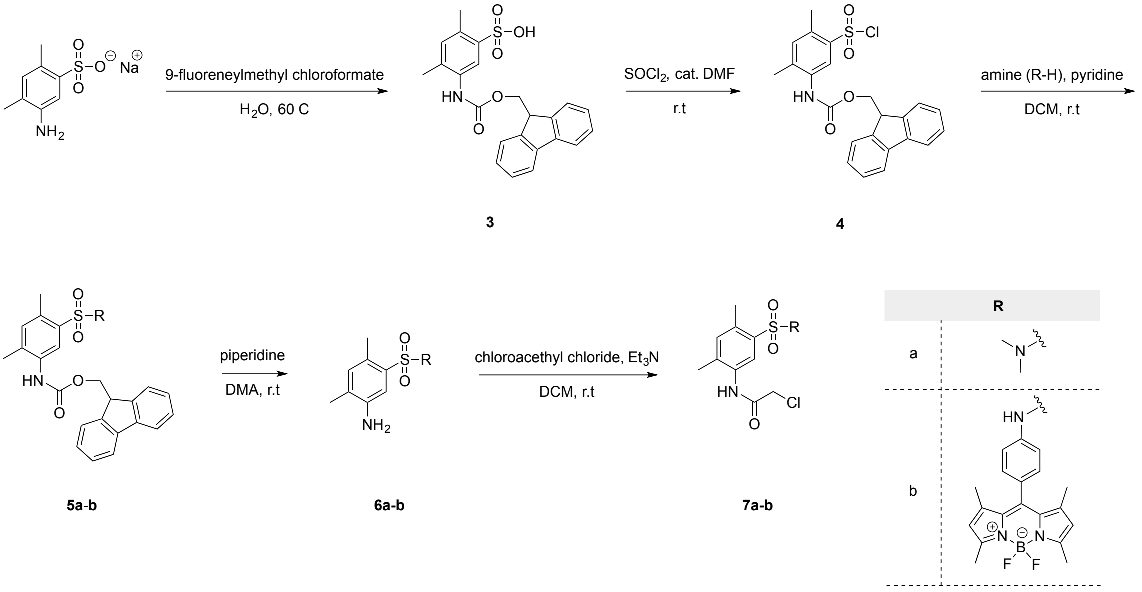

Supplementary Fig. 9. Synthetic scheme of C1-27A and BODIPY-conjugated C1-27A.

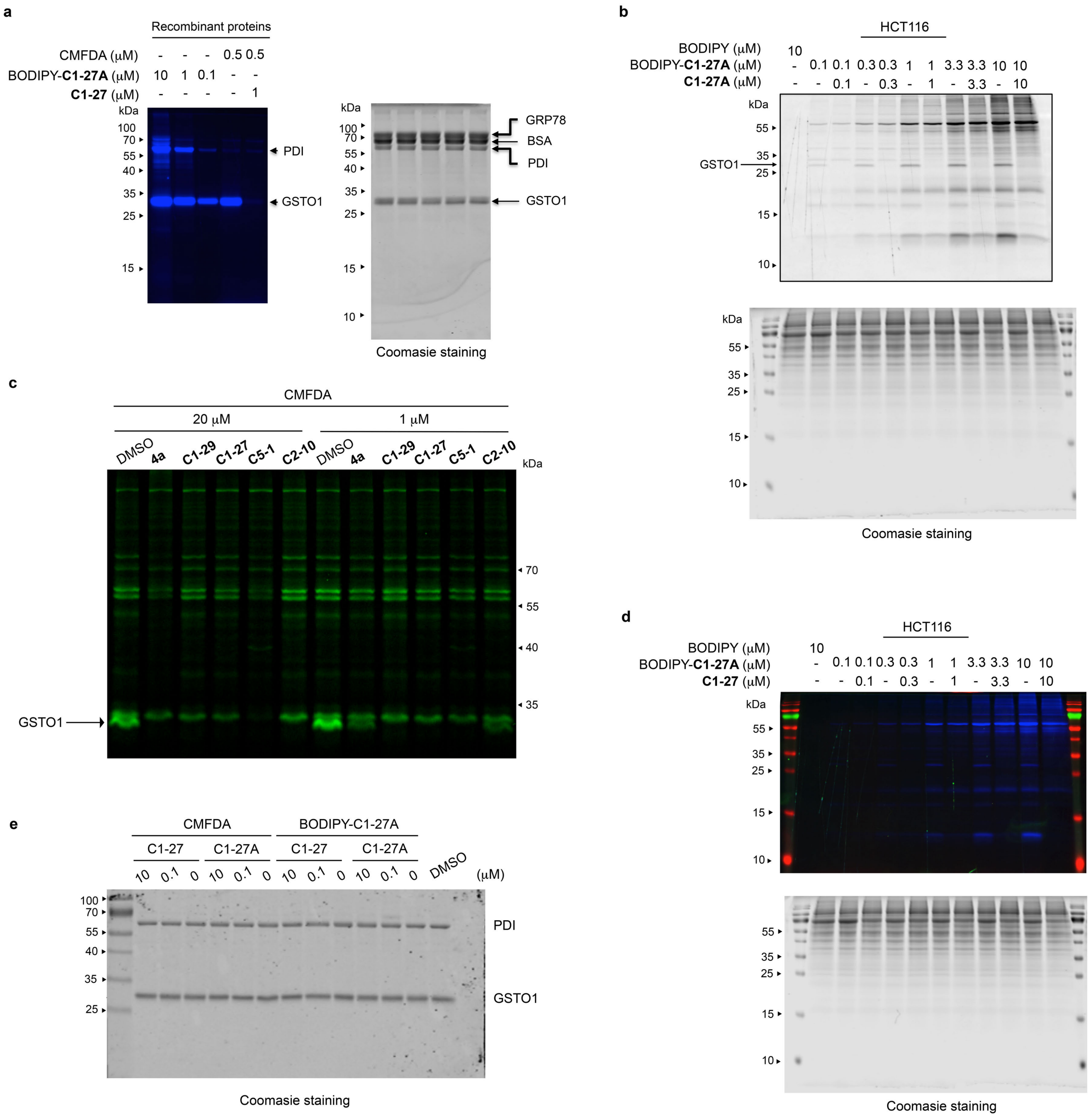

Supplementary Fig. 10. Selectivity of GSTO1 inhibition. (**a**) A cocktail of recombinant proteins was incubated with BODIPY-C1-27A (1 μM), CMFDA (500 nM) or C1-27 (1 μM). Fluorescent gel image (*left*) and coomasie staining (*right*) are shown. (**b**) Protein labeling by BODIPY-**C1-27A** in HCT116 cells in the presence or absence of pretreatment with unconjugated **C1-27A** at indicated concentrations. Representative image of fluorescence scan (*above*) and coomasie staining (*below*) of two independent experiments are shown. (**c**) HCT116 soluble proteome was incubated with indicated GSTO1 inhibitors at 20 μM and at 1 μM for 2 h, followed by addition of 500 nM CMFDA for 1 h. Selectivity of GSTO1 inhibition versus inhibition of CMFDA binding to various other cysteine-bearing proteins in the proteome was analyzed. (**d**) Fluoroscent scan (RGB image) and coomasie staining of gel for experiment in Fig. 1f are shown. (**e**) Coomasie staining of gel for experiment in Fig. 1g.

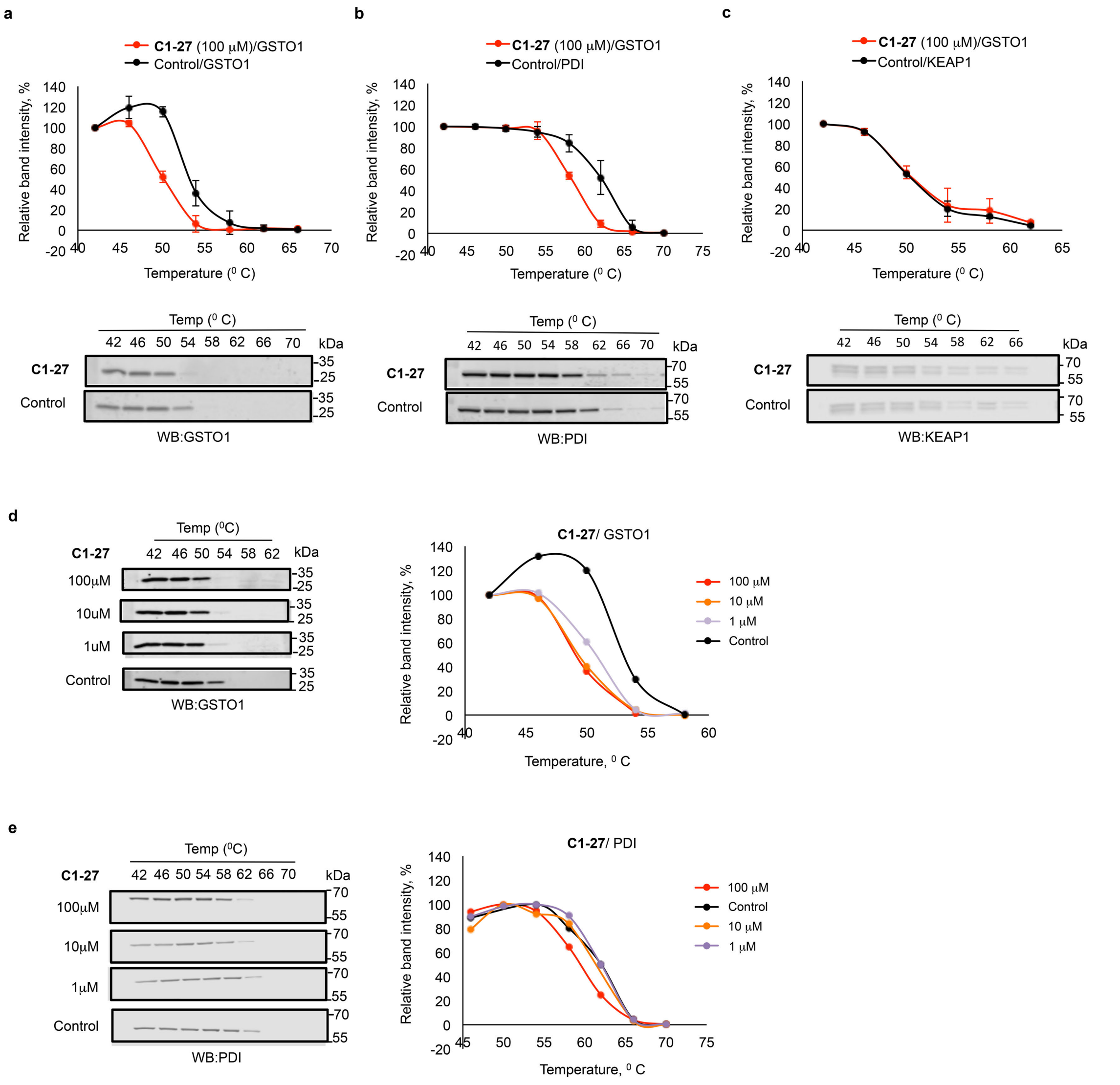

Supplementary Fig. 11. Target engagement by C1-27. (**a-c**) CETSA melt curves for GSTO1 (**a**), PDI (**b**) and Keap1 (**c**) in HCT116$^{p53+/+}$ cell lysate treated with **C1-27** (100 μM). 1% DMSO was used as control. Data presented are mean ± SD of 3 independent experiments. A negative shift in the melting curves was detected for GSTO1 and PDI while no shift was observed for Keap1. (*Below*) Representative western blots showing soluble fractions of the target proteins following drug treatment and heating. (**d-e**) Western blots (*left*) and CETSA melt curves (*right*) for GSTO1 (**d**) and PDI (**e**) in HCT116$^{p53+/+}$ cell lysate treated with different concentrations of **C1-27**. At a higher concentration (100 μM), **C1-27** showed a thermal shift with PDI, but at lower concentrations (10 μM and 1 μM), no shift was observed. On the other hand, for the target GSTO1, **C1-27** showed significant shifts at all three concentrations. These findings suggest that even at lower concentrations, **C1-27** shows significant target engagement with GSTO1.

a

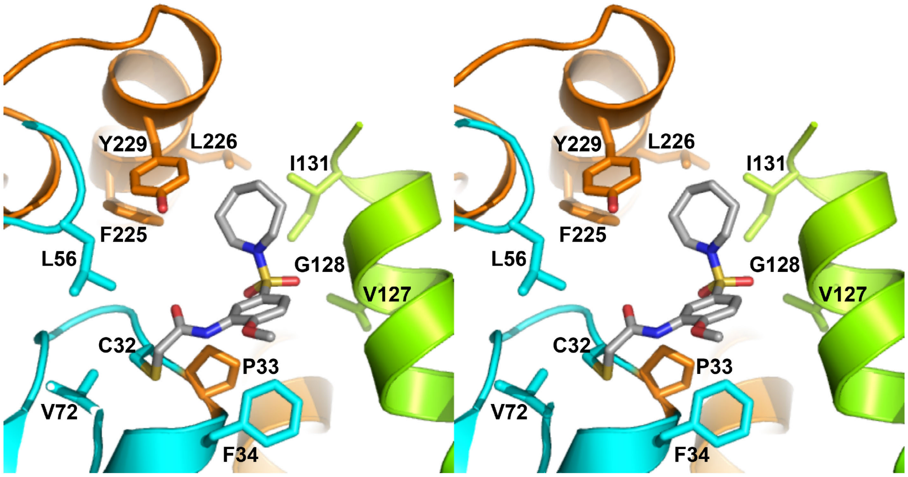

b

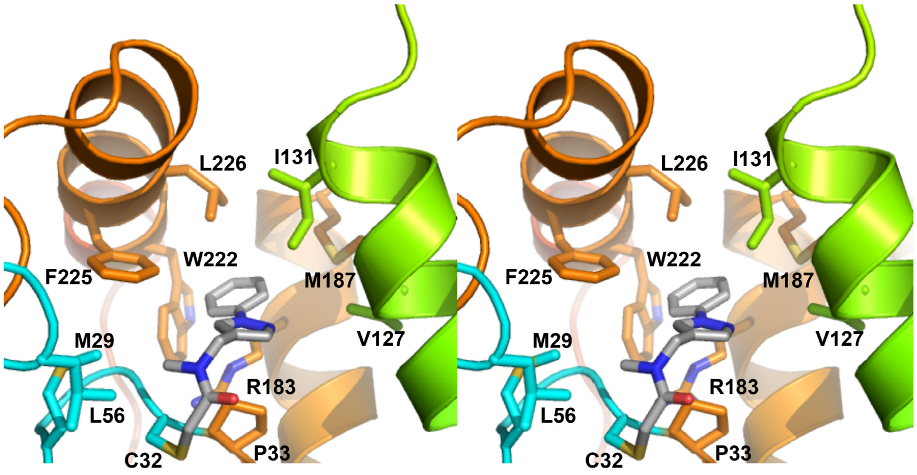

Supplementary Fig. 12. GSTO1 inhibitor binding site. Stereodiagrams of (**a**) **C1-31** (PDB ID 4YQU) and (**b**) **C4-10** (PDB ID 4YQV) bound to GSTO1 in the cocrystal. A ribbon diagram represents the backbone of GSTO1 with residues interacting with the inhibitor shown as ball-and-stick. Residues in cyan contribute to the G-site, while the H-site is made of the residues shown in orange and green (4b helix). The inhibitors are shown in ball-and-stick with carbon atoms in grey; sulfurs in yellow; nitrogens in blue; oxygens in red; and chloride in green. The RMSD between **C1-27** and **C1-31**, and **C4-10** is 0.535 Å and 0.573 Å, respectively.

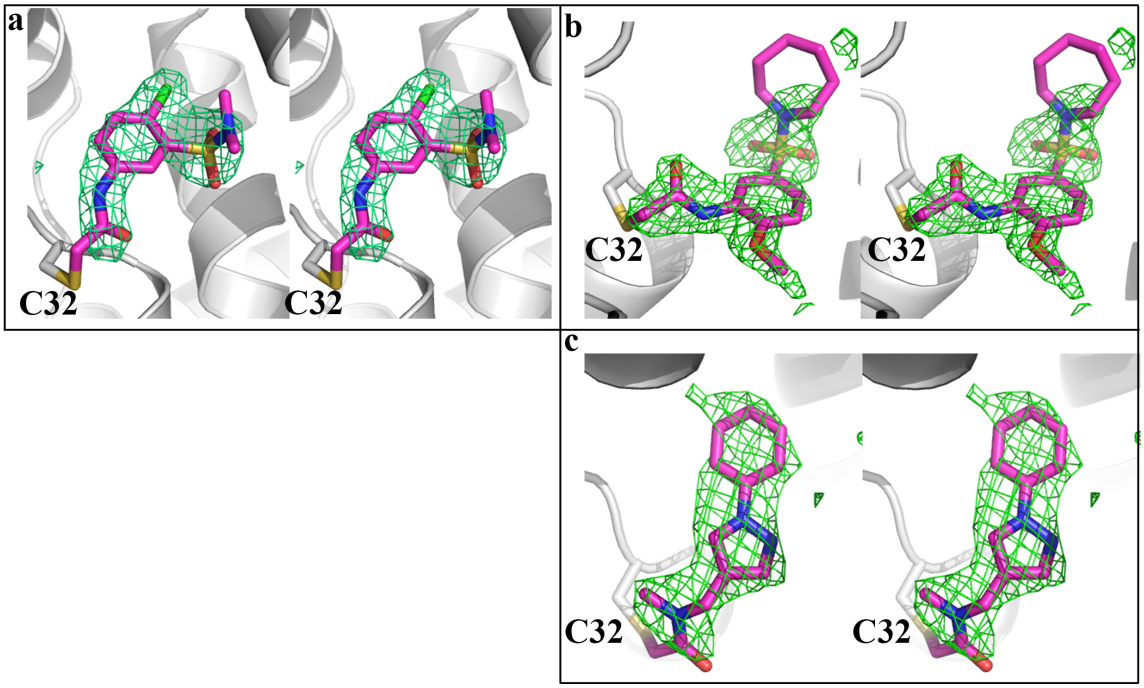

Supplementary Fig. 13. Omit electron density maps for GSTO1 inhibitors. Omit (Fo-Fc) electron density maps shown as stereodiagrams for (**a**) GSTO1-**C1-27**, (**b**) GSTO1-**C1-31**, and (**c**) GSTO1-**C4-10** were calculated in Buster[1], contoured at 3 σ and displayed as green grids using PyMol. Each inhibitor is shown as magenta ball-and-stick along with the covalently bound C32; the GSTO1 backbone is shown in grey.

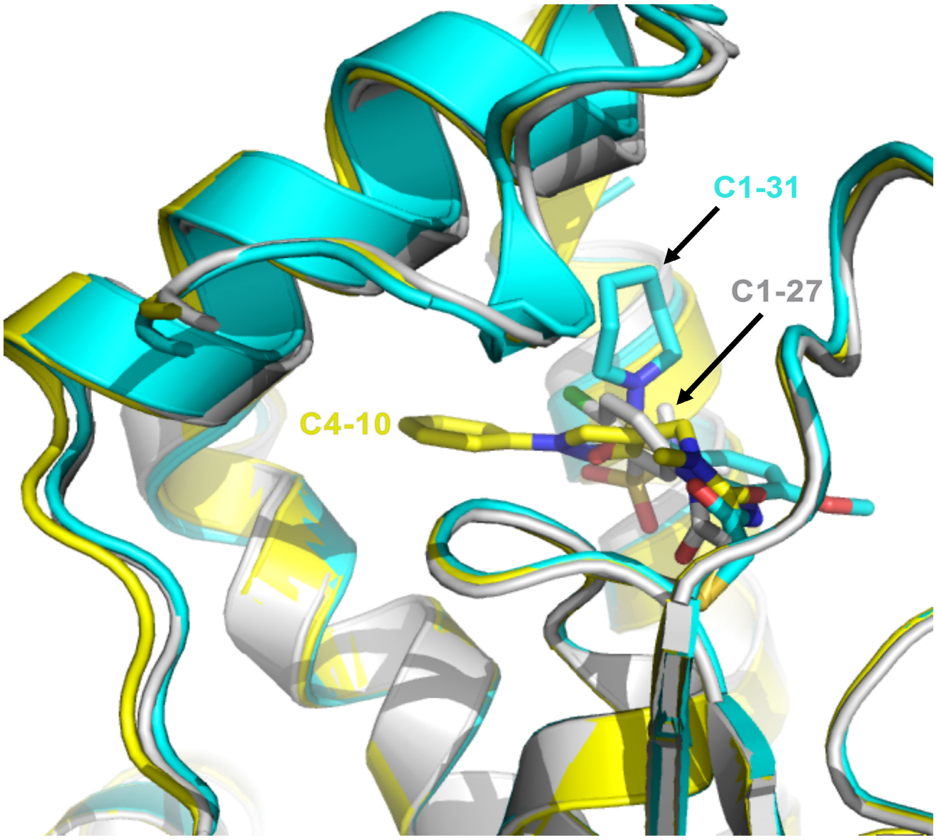

Supplementary Fig. 14. Overlay of GSTO1 bound to inhibitors. The protein backbones are displayed as a ribbon diagrams **C1-27** (light gray), **C1-31** (cyan) and **C4-10** (yellow) with the various inhibitors in ball-and-stick (carbon atoms are shown in the protein color, sulfur atoms (yellow), nitrogens (blue), chloride (green) and oxygens (red).

a

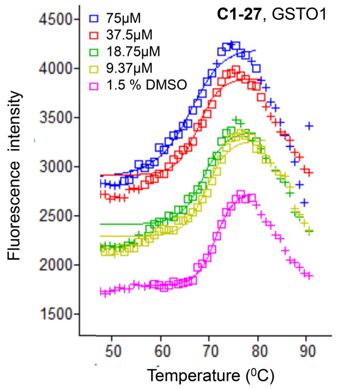

b

C1-27(μM)	Tm (Av)	ΔTm (⁰C)
75.0	65.09 ± 0.11	-5.67
37.5	67.32 ± 0.16	-3.44
18.7	68.24 ± 0.11	-2.52
9.4	68.93 ± 0.09	-1.83
DMSO	70.76 ± 0.13	

c

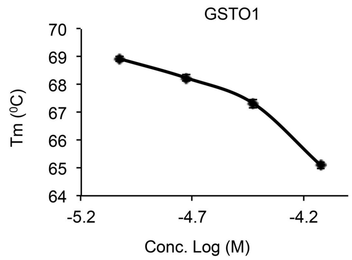

d

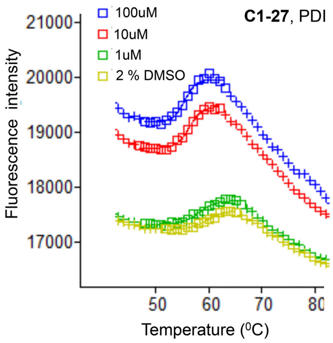

e

C1-27(μM)	Tm (Av)	ΔTm (⁰C)
100	54.49 ± 0.08	-3.85
10	55.09 ± 0.19	-3.25
1	57.26 ± 0.11	-1.08
DMSO	58.34 ± 0.15	

f

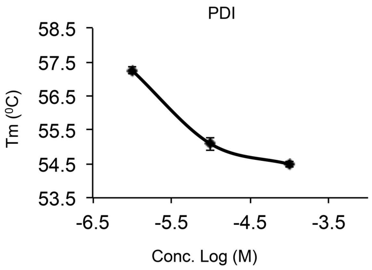

Supplementary Fig. 15. Thermal stability assay performed in the presence of C1-27. (**a**, **d**) Thermal shift assays were performed at various concentrations of **C1-27** with GSTO1 (0.3mg/ml) (**a**) and PDI (0.3mg/ml) (**d**) using Thermofluor. Fluorescence intensity as a function of temperature at different concentrations is plotted. (**b**, **e**) Table shows the melting temperature Tm, midpoint of the unfolding transition extracted from the melting curves, for GSTO1 (**b**) or PDI (**e**) in the presence of different concentrations of **C1-27** or DMSO. Data are mean ± SD from three independent experiments. (**c**, **f**) Plot of **C1-27** concentration against melting temperature, Tm, suggests a decrease in protein stability upon **C1-27** binding to GSTO1 (**c**) and PDI (**f**). Data are mean ± SD.

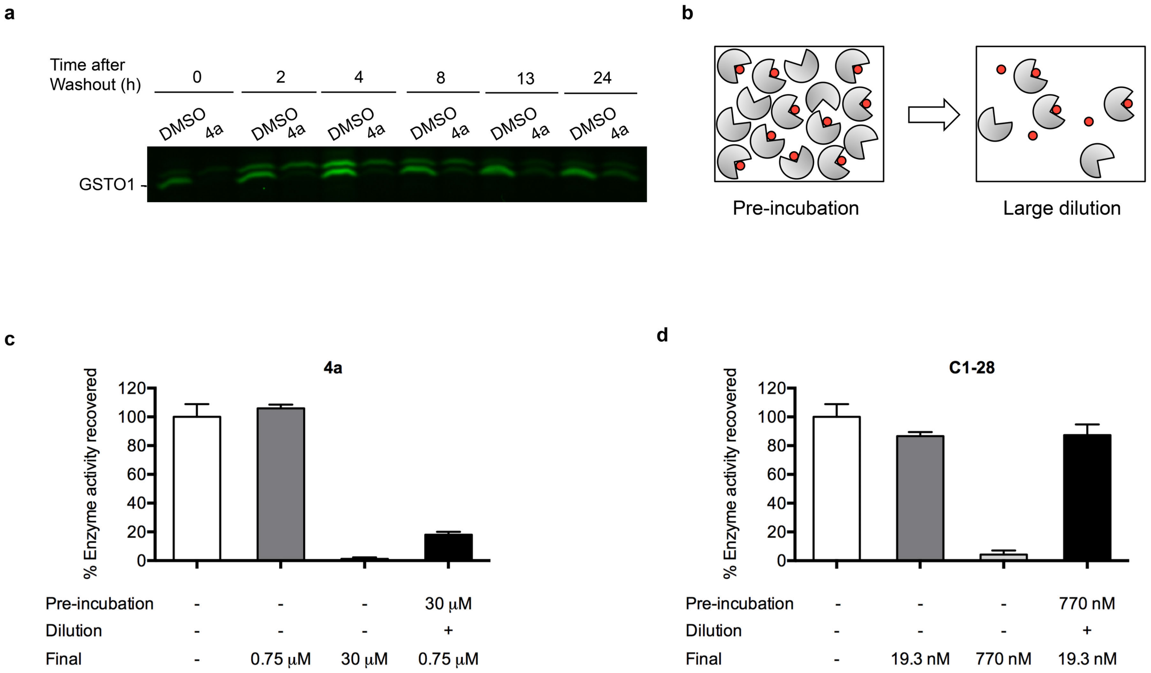

Supplementary Fig. 16. Mechanistic studies. (**a**) Compound **4a** shows sustained GSTO1 inhibition up to 24 h in HCT116 cells incubated with 5 μM **4a** for 2 h, followed by washout. (**b**) Schematic of the experiment to analyze reversibility of inhibition. (**c**) Compound **4a** shows an irreversible mode of GSTO1 enzyme inhibition. On the other hand, compound **C1-28** (**d**) appears to act like a slow-turnover substrate. Data are mean ± SEM, performed in triplicate.

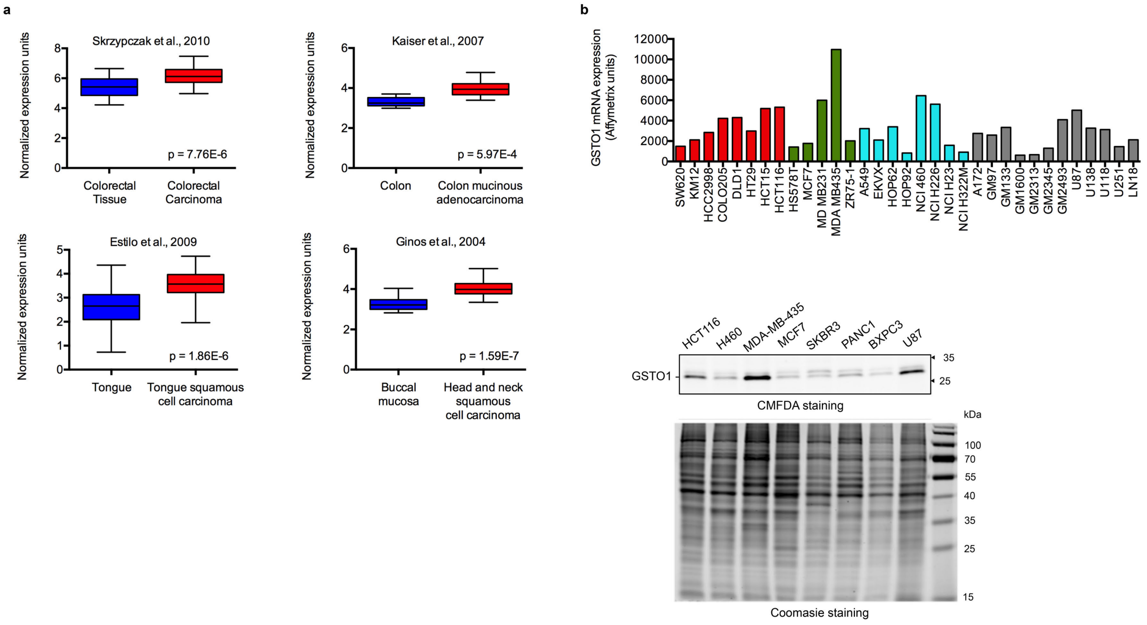

Supplementary Fig. 17. GSTO1 is overexpressed in several cancers. (**a**) GSTO1 overexpression in select cancers analyzed using the Oncomine database. Box and whiskers plots show representative studies with normalized GSTO1 expression in cancer versus normal tissue. (**b**) GSTO1 overexpression in various cancer cell lines analyzed using BioGPS database. (*Below*) GSTO1 expression in different cancer cell lines, assessed by fluorescence scan of CMFDA binding to GSTO1 (Grayscale image shown for clarity).

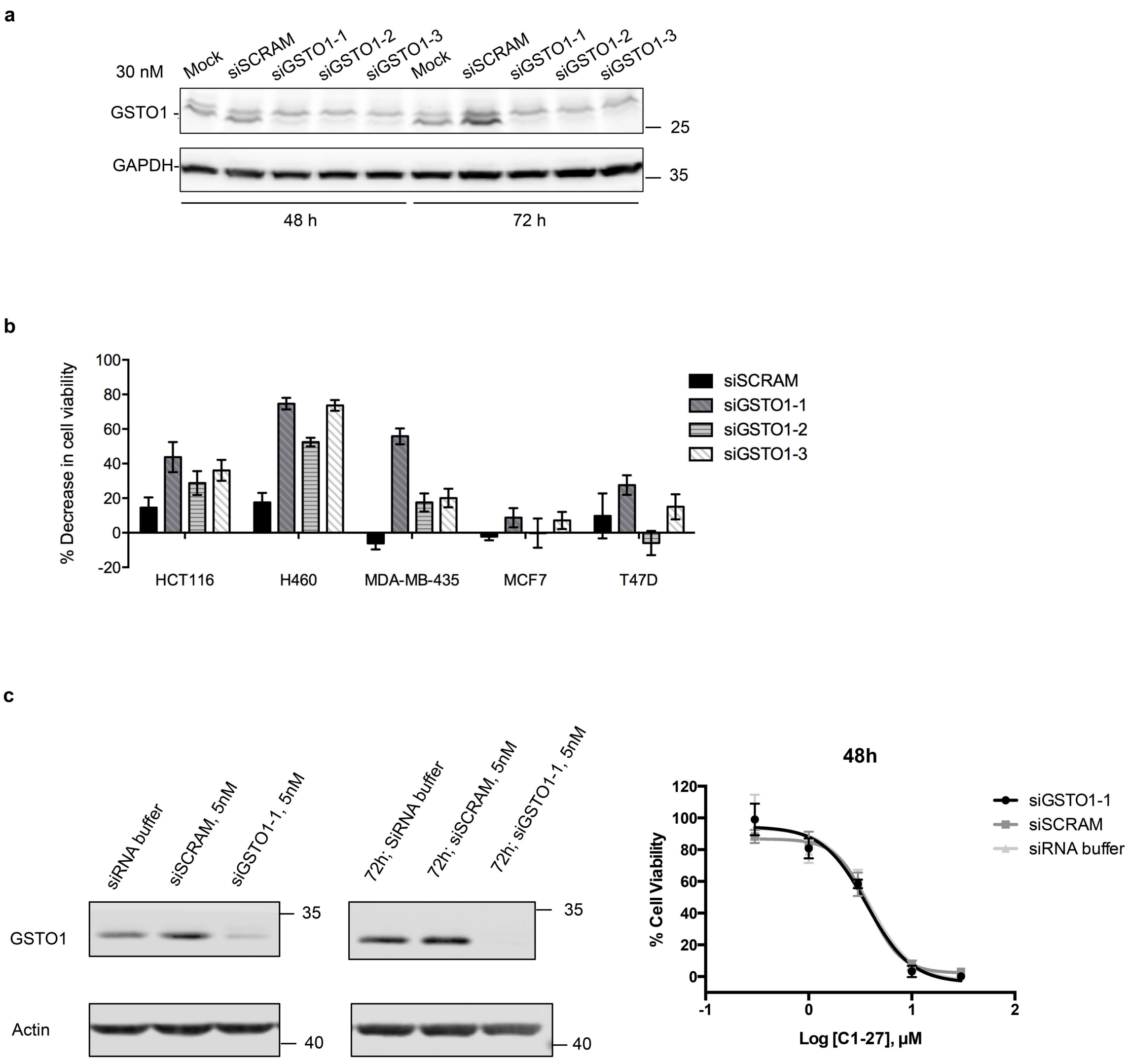

Supplementary Fig. 18. GSTO1 is important for cancer cell viability. (**a**) GSTO1 expression at 48 h and 72 h post-transfection with 30 nM GSTO1-specific siRNA or scrambled control, assessed by in-gel fluorescence scanning using CMFDA. GAPDH as loading control. (**b**) GSTO1 knockdown decreases cancer cell viability. Cell lines (HCT116, H460, MDA-MB- 435, MCF7, T47D, 10,000 cells/well) were transfected with 3 different GSTO1-specific siRNAs or siSCRAM (10 nM). Cell viability was measured 72 h post transfection using MTT assay and compared against siSCRAM-transfected cells. Percent decrease in cell viability is shown. Data are mean ± SEM of replicate wells. GSTO1 siRNA had minimal non-specific cytotoxicity in T47D cells, which are devoid of mature GSTO1 protein. (**c**) HCT116 cells were transfected with GSTO1-specific siNAs (siGSTO1-1, 5 nM) or scrambled control siRNA (siSCRAM, 5 nM) for 48 h. GSTO1 knockdown at 48 h and 72 h was confirmed by western blotting using anti-GSTO1 antibody (*left*). At 48 h post-transfection, siRNA-treated HCT116 cells were treated with **C1-27** at indicated concentrations for an additional 48 h. Cell viability was measured by MTT assay (*right*). Data are mean ± SEM of triplicate wells.

a

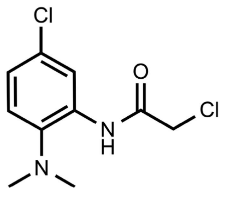

C1-14

b

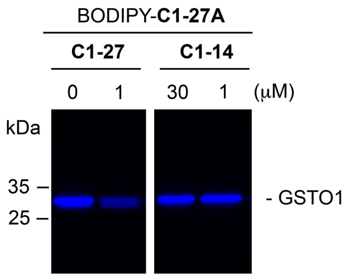

c

	GI_{50}	% Inhibition
MTT assay	>30 μM	
PDI-catalyzed insulin reductase assay		9.6% at 40 μM

d

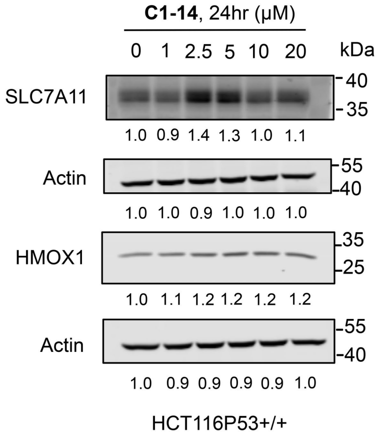

Supplementary Fig. 19. Characterization of the inactive control, C1-14. (**a**) **C1-14** structure (**b**) Competitive binding assay using BODIPY-**C1-27A** shows lack of **C1-14** binding to recombinant GSTO1 at indicated concentrations. (**c**) **C1-14** cytotoxicity and activity against PDI are shown. (**d**) Western blotting of SLC7A11 and HMOX1 expression levels after 24 h treatment with **C1-14** at indicated concentrations. Normalized relative densities computed using Image J are indicated.

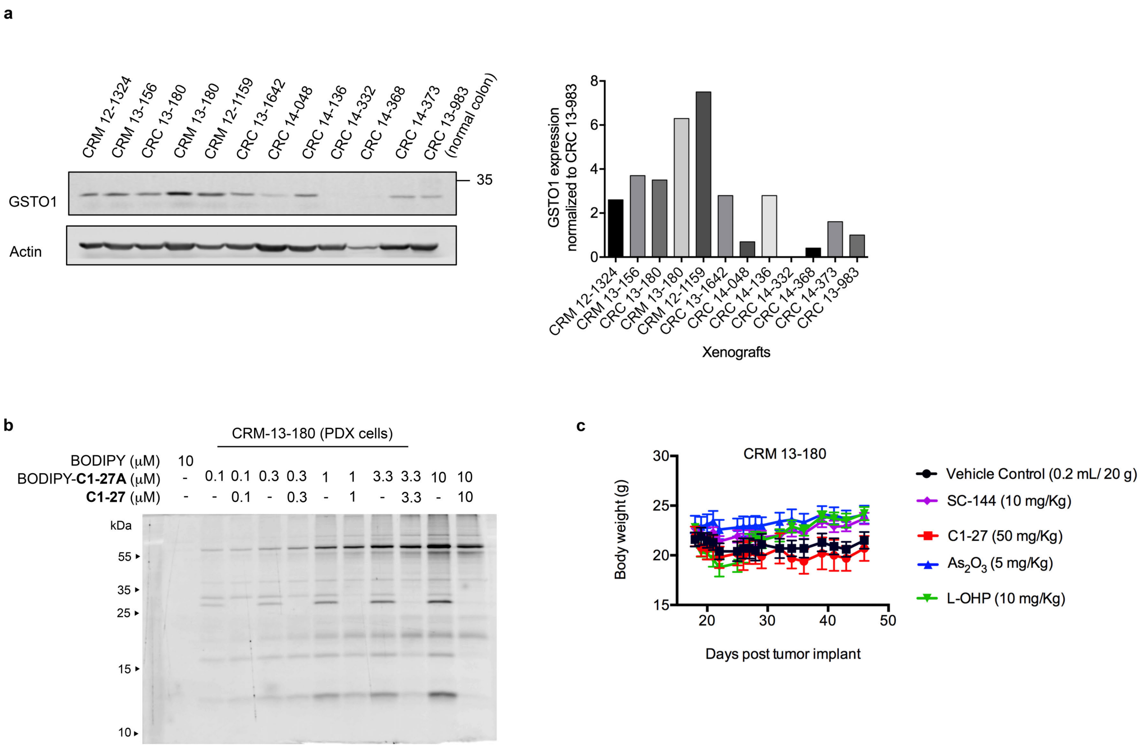

Supplementary Fig. 20. (**a**) GSTO1 expression in 11 colorectal cancer PDX models, assessed by western blotting. CRM 13-180 shows high GSTO1 expression. (**b**) Protein labeling by BODIPY-**C1-27A** in CRM 13-180 (PDX) cells in the presence or absence of pretreatment with **C1-27** at indicated concentrations. Fluorescence scan shown as grayscale image for clarity. (**c**) Body weights of CRM 13-180 tumor-bearing mice treated with **C1-27**, L-OHP, SC-144, As_2O_3 or vehicle over the duration of treatment. Data are mean ± SEM.

Cannonical pathway	P-value
Estrogen-mediated S-phase Entry	0.00020
p53 Signaling	0.00022
Aryl Hydrocarbon Receptor Signaling	0.00029
Cell Cycle: G1/S Checkpoint Regulation	0.00110
Serine Biosynthesis	0.00151
GADD45 Signaling	0.00158
Small Cell Lung Cancer Signaling	0.00191
Glioblastoma Multiforme Signaling	0.00224
HER-2 Signaling in Breast Cancer	0.00257
Superpathway of Serine and Glycine Biosynthesis I	0.00309
Superpathway of Cholesterol Biosynthesis	0.00447
Molecular Mechanisms of Cancer	0.00537
Chronic Myeloid Leukemia Signaling	0.00575
Retinoate Biosynthesis I	0.00603
Oleate Biosynthesis II (Animals)	0.00646
Telomerase Signaling	0.00692
ILK Signaling	0.00759
Regulation of the Epithelial-Mesenchymal Transition Pathway	0.00776
Cell Cycle Regulation by BTG Family Proteins	0.00933
HGF Signaling	0.00955

Supplementary Fig. 21. Top 20 canonical pathways identified by Ingenuity pathway analysis (IPA) associated with GSTO1 knockdown.

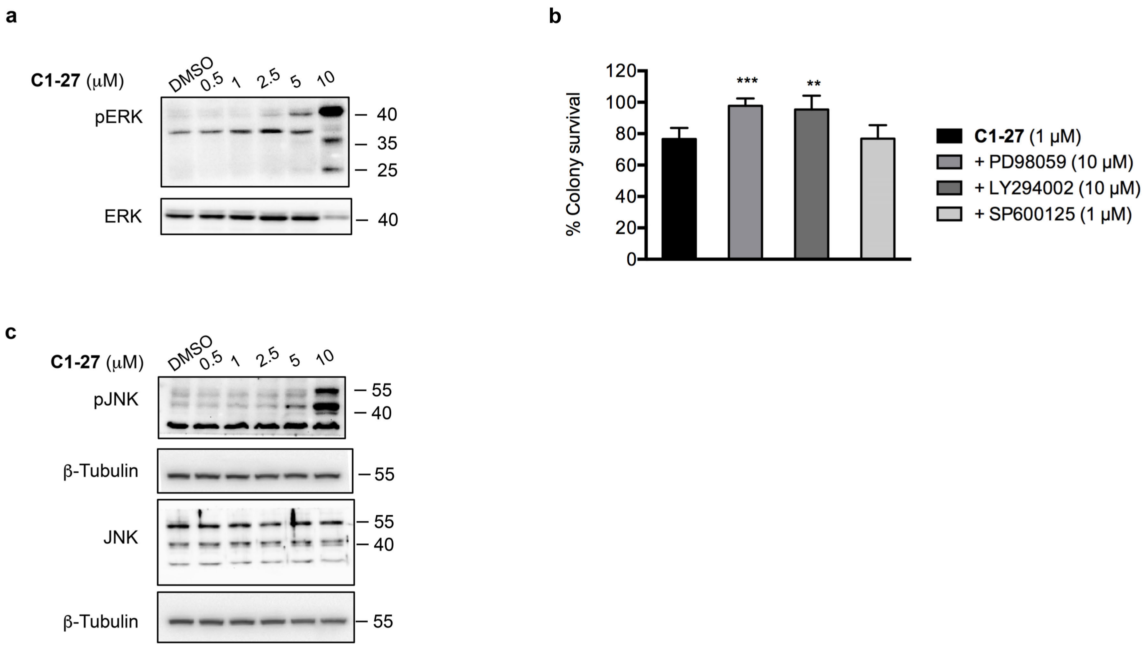

Supplementary Fig. 22. Cell death induced by C1-27 is Ras-signaling dependent. Western blotting of HCT116 cells shows induction of (**a**) pERK and (**b**) pJNK in response to **C1-27** treatment at indicated concentrations for 24 h. (**c**) Cell death mediated by **C1-27** is MEK and PI3K-dependent and JNK-independent. HCT116 cells were treated with **C1-27** (1 μM) alone or in combination with MEK inhibitor (PD98059, 10 μM), PI3K inhibitor (LY294002, 10 μM) or JNK inhibitor (SP600125, 1 μM) and colonies were grown for 7-10 days. Percent colony survival depicted are mean ± SD of 3 independent experiments.

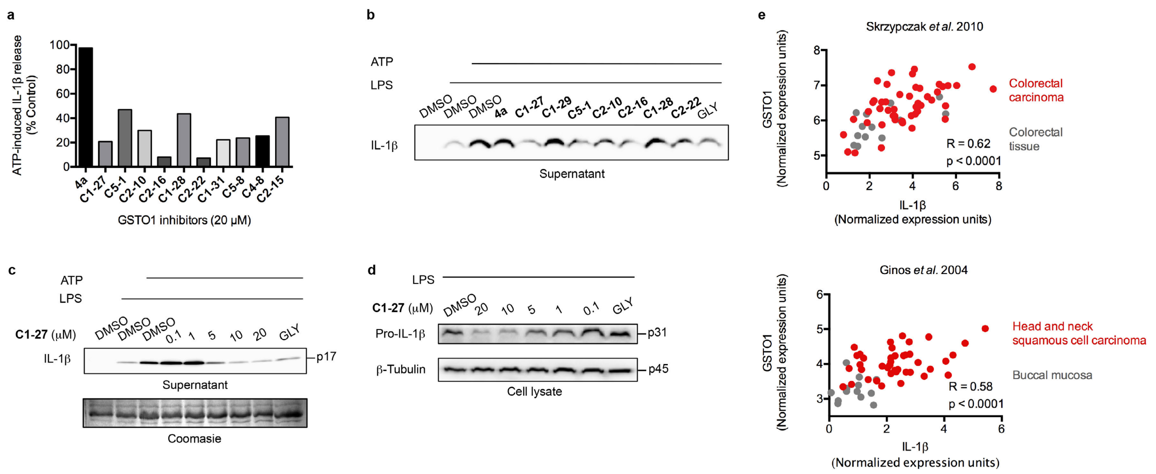

Supplementary Fig. 23. GSTO1 inhibitors suppress IL-1β secretion. (**a**) ATP-induced IL-1β secretion from LPS-stimulated THP-1 cells was analyzed by ELISA. THP-1 cells were stimulated with LPS (200 ng/mL) for 4 h and treated with 20 μM of indicated GSTO1 inhibitors in serum-free media for 1 h followed by addition of 5 mM ATP for 3 h. Cytokine secretion level is expressed as a percentage of that released from control cells treated with DMSO. Data are mean readings of duplicate wells. There was less than 15% decrease in cell viability during the 4 h treatment with most of the compounds. (**b**) Inhibition of IL-1β secretion by GSTO1 inhibitors was further confirmed by Western blotting. Supernatant media from LPS-primed THP-1 cells treated with GSTO1 inhibitors (20 μM) or Glyburide (GLY, 50 μM) for 1.5 h and ATP (5 mM) for 30 min was concentrated and precipitated using TCA and immunoblotted for mature IL-1β (17 kDa). (**c**) Dose-dependent inhibition of IL-1β release by **C1-27**. LPS-primed THP-1 cells were treated with indicated concentrations of **C1-27** or GLY (50 μM) for 1.5 h, followed by stimulation with ATP (5 mM) for 30 min. Concentrated supernatant was analyzed by western blotting using anti IL-1β antibody. Representative blot from one of three independent experiments is shown. (**d**) **C1-27** inhibits pro-IL-1β production at higher concentrations. THP-1 cells were treated with indicated concentrations of **C1-27** or GLY (50 μM) for 30 min, followed by LPS stimulation for 3 h. Expression of pro-IL-1β in response to LPS was analyzed by western blotting. Representative blot from one of two independent experiments is shown. (**e**) IL-1β expression strongly correlates with GSTO1 expression. Representative plots of several gene expression datasets analyzed in Oncomine are shown. Each point represents the expression of GSTO1 (x-axis) and IL-1β (y-axis) in the cells of the same patient. Pearson's correlation coefficient R is indicated.

Fig. 1d

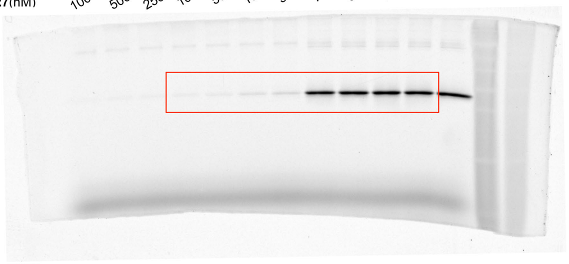

Recombinant GSTO1

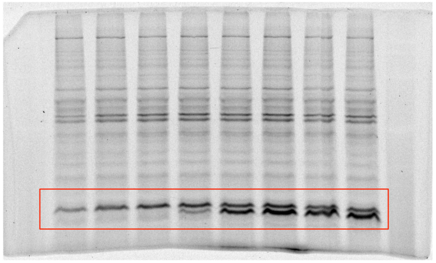

Endogenous GSTO1

Fig. 2c

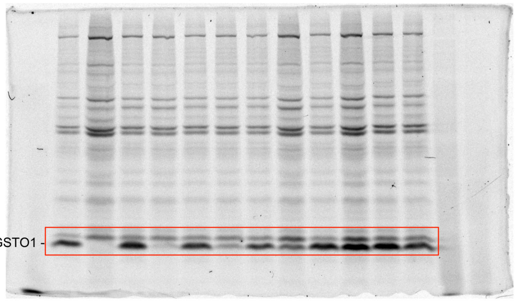

Supplementary Fig. 24. Uncropped gels for Figure 1d and 2c.

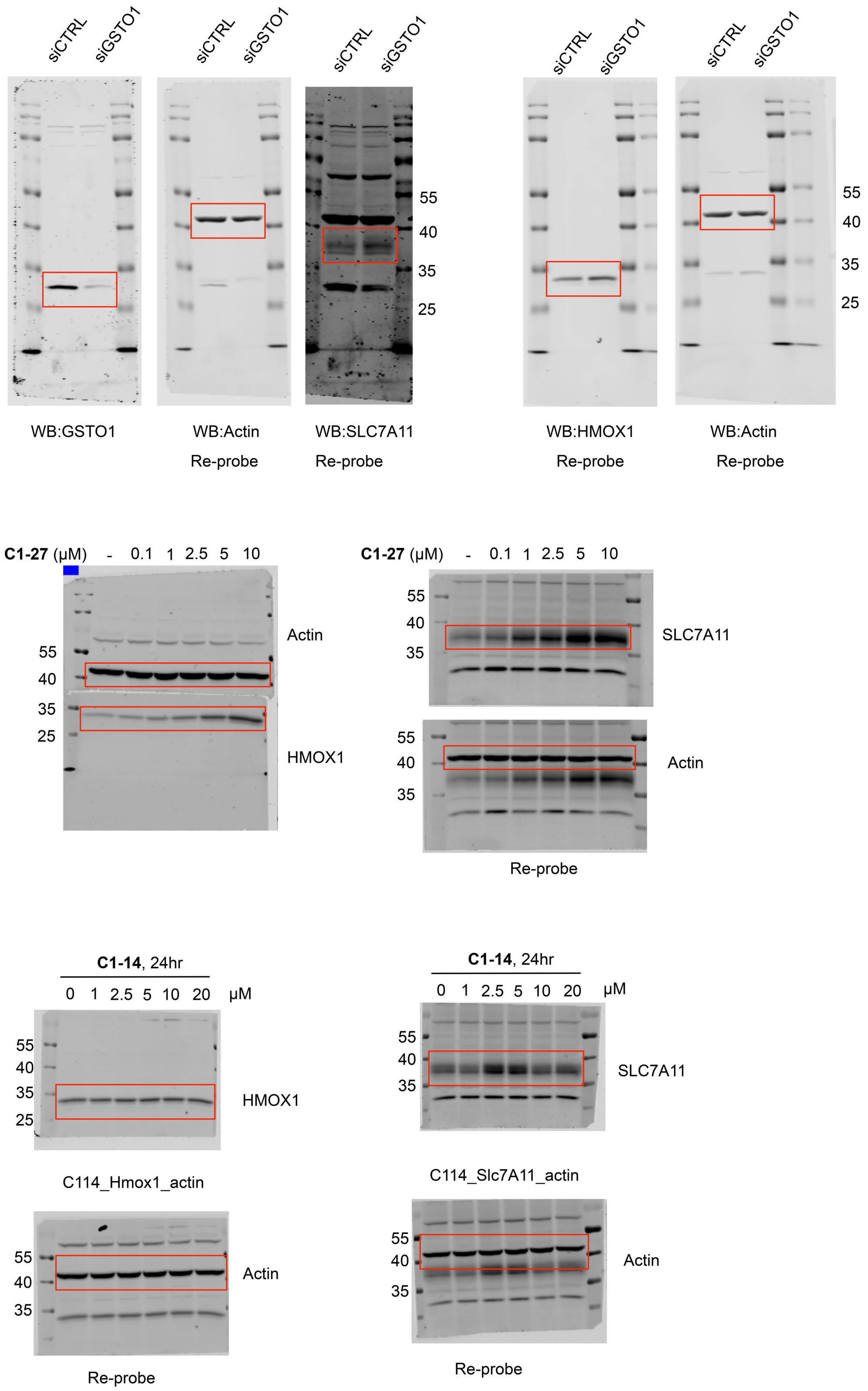

Supplementary Fig. 25. Uncropped membranes for Figure 4b.

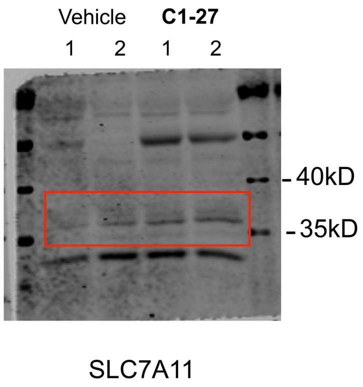

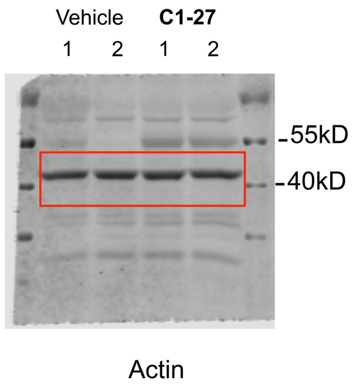

Supplementary Fig. 26. Uncropped membranes for Figure 5g.

Supplementary Table 1. Results of pilot screening

Cpd	Structure	Substrate assay
		IC_{50} (μM)
**2a**	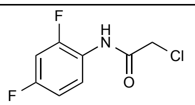	3.5
**2b**	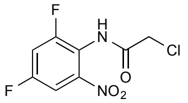	1.7
**2c**	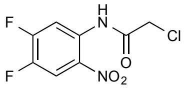	0.71
**4a**	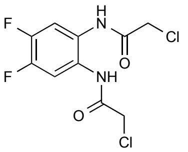	3.1
**4b**	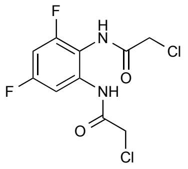	3.9
**4c**	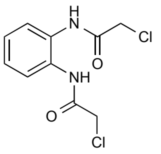	80.8
**6**	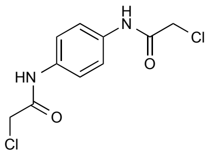	>100

Supplementary Table 2. Screening data for Cluster-1 - *N*-Phenyl-2^0-chloroacetamide

	GSTO binding assay (% Inhibition at 10 μM)		GSTO substrate assay		% Inhibition of HCT116p53+/+ cell proliferation at 10 μM	MW	HBA	HBD	LogP
	Recombinant	Endogenous	% Inhibition at 10 μM	IC_{50} (μM)					
**C1-1**	17.7	16.2			14, 21.3	253.77	2	1	3.89
**C1-2**	54.1	66.2	100.9	1.22	7.4	249.05	4	1	2.42
**C1-3**	0.9	3.8	-4.9			372.41	4	1	2.77
**C1-4**	81.5	31.8	92.5		80.6	323.71	5	2	3.97
**C1-5**	52.9	22.9	96.3		-3.8, -2.6	241.74	2	1	2.85
**C1-6**	45.6		87.6		22.7	235.62	3	1	1.95
**C1-7**	75.6	56.6	77.7	0.162	29.6	340.61	3	1	4.12
**C1-8**	61.2	56.5	100.9	5.68		285.69	6	1	2.13
**C1-9**	56.5	39.3	12.5		-1.2	255.66	5	1	1.15
**C1-10**	49.1	49.7	81.7		19.7	278.09	5	2	2.42
**C1-11**	55.2	58.6	77.7	1.16	63.4, 65.6	276.12	4	1	2.13
**C1-12**	26.5	40.1	10.4	85		334.16	4	1	2.98
**C1-13**	35.2	61.1	62.2		14.3, 6.6	292.72	5	2	1.68
**C1-14**	-15.6	6.7	16.5			247.13	3	1	2.65
**C1-15**	43.8		78.6		91.3	254.76	3	1	2.89
**C1-16**	49.2	46.7	87.8		41.9	252.75	3	1	2.81
**C1-17**	-4.1	-9.5	88.5		94.8, 91	306.72	3	1	3.55
**C1-18**	6.4	54.4	3.7		41.5, 41.7	252.70	4	1	1.46
**C1-19**	38.3	65.7	61.5		79.2, 85.4	266.77	3	1	3.34
**C1-20**	-8.5	-15.1	66.9		43.7, 39	281.79	4	1	2.13
**C1-21**	69.9	58.2	-8.4			254.72	4	1	1.56
**C1-22**	25.1	49.0	41.4		80.8, 86	252.70	4	1	1.29
**C1-23**	56.6	63.0	89.8	4.79	36.5	304.78	4	1	1.77
**C1-24**	68.4	62.1	77	0.148	89.5, 91.8	283.68	4	1	1.9
**C1-25**	70.3		9.8		11.3, 21.7	344.86	5	1	2.63

**C1-26**	51.4	48.9	31.1		13.2	304.80	5	1	1.33
**C1-27**	69.8		88.3	0.031	93.8	311.19	5	1	1.62
**C1-28**	71.5	57.2	87.6	0.064	94.4	338.81	5	1	2.31
**C1-29**	70.6	61.0	102.3	0.030	42.5	470.16	6	1	3.61
**C1-30**	75.9	80.8	75	0.079	45, 20.7	360.86	6	1	1.93
**C1-31**	60.0	77.0		0.221	88.7, 83.5	360.86	6	1	2.33
**C1-32**	4.4	48.2	84.2			389.90	7	1	1.61

Supplementary Table 3. Screening data for Cluster-2 - *N*-Heterocycle-2^0-chloroacetamide

	GSTO binding assay (% Inhibition at 10 μM)		GSTO substrate assay		% Inhibition of HCT116p53+/+ cell proliferation at 10 μM	MW	HBA	HBD	LogP
	Recombinant	Endogenous	% Inhibition at 10 μM	IC_{50} (μM)					
**C2-1**	-0.8	45.0	54.8		65.6, 62.7	204.70	3	1	0.85
**C2-2**	6.5	66.7	90.5		1.1	266.77	3	1	1.98
**C2-3**	37.8	47.0	61.5		49	305.74	3	1	2.95
**C2-4**	35.8	51.3	0.5		81.4, 84.4	277.76	3	1	2.29
**C2-5**	64.1	69.4	4.4		-2.7, -8.6	275.74	3	1	2.12
**C2-6**	36.8	25.2	84.5		-17, -9.7	260.68	4	1	1.43
**C2-7**	56.0	55.3	79.7	0.598	85.7, 91	284.15	3	1	2.39
**C2-8**	39.2	51.7	3.8		4.5, 0.8	289.78	4	1	3.37
**C2-9**	50.0	59.2	13.9		16.3	358.25	4	1	4.24
**C2-10**	48.4	51.6	88.4	0.062	9.1	344.22	4	1	3.89
**C2-11**	58.2	19.9	-1.5			323.80	4	1	3.88
**C2-12**	74.9	59.8	3.4			383.85	6	1	3.38
**C2-13**	48.1	41.3	25.9		38.1	395.86	6	1	3.78
**C2-14**	57.9	58.2	-7.0		6.3	408.91	6	2	4.01
**C2-15**	63.2	74.2	87.8	0.360		378.88	5	1	2.62
**C2-16**	54.7	53.0	73.0	0.066		315.82	4	1	3.86
**C2-17**	51.5	24.8	77.2		85.2, 94.5	330.84	5	1	2.7
**C2-18**	72.8	78.7		0.119	82.1	286.78	4	2	2.39
**C2-19**	51.8	58.6	1.2		52.7, 40.5	458.97	5	2	3.14
**C2-20**	68.3	39.6	91.1			333.84	3	1	4.1
**C2-21**	78.6	52.3	99.5	0.350		426.97	4	1	5.73
**C2-22**	80.3		84.9	0.136	75.7	297.72	5	1	2.32
**C2-23**	76.0		26.4			294.80	3	1	3.3
**C2-24**	82.4	62.1	34.0		22.9, 19.2	298.75	5	2	2.4
**C2-25**	75.7		3.7		0.5, 4.3	291.76	3	2	2.58

**C2-26**	72.9		80.0	0.125	93.8	301.20	3	1	3.18
**C2-27**	69.0	59.6	85.1	8.669	42.6, 38.2	284.72	5	1	1.88
**C2-28**	59.5		-2.8		88.5	364.83	4	1	4.45
**C2-29**	13.5	65.0	42.7		8.5, 29.1	295.73	6	2	0.88
**C2-30**	50.5	41.1	47.4		12.0	271.73	4	1	1.58

Supplementary Table 4. Screening data for Cluster-3 - *N*-Linker-2^0-chloroacetamide

	GSTO binding assay (% Inhibition at 10 μM)		GSTO substrate assay		% Inhibition of HCT116p53+/+ cell proliferation at 10 μM	MW	HBA	HBD	LogP
	Recombinant	Endogenous	% Inhibition at 10 μM	IC_{50} (μM)					
**C3-1**	6.0	7.3	11.1			282.77	4	1	1.32
**C3-2**	63.3	63.6	86.4	1.48	55.3, 49.6	318.82	5	1	1.36
**C3-3**	47.9	52.0	93.2			244.64	5	2	1.61
**C3-4**	-2.6	31.6			16, 17.5	273.72	5	1	1.19
**C3-5**	14.7	31.8	85.1		64.1, 63.6	283.76	4	1	2.13
**C3-6**	53.7	51.3	56.2	7.88	13.6, 34.7	267.80	2	1	3.64
**C3-7**	12.3	12.6	12.5		73.6	241.68	4	1	1.48
**C3-8**	-4.9	33.4	16.5		91.2, 77.9	283.76	4	1	2.06
**C3-9**	30.7	53.9	80.7		24.9	241.68	4	1	1.21
**C3-10**	29.4	48.4	78.4		96.5, 87.4	263.73	3	1	1.29
**C3-11**	10.1	45.6	9.8		8.1, 16.1	293.70	4	1	2.15
**C3-12**	22.0	9.7	83.2		5	240.73	3	1	1.55
**C3-13**	25.1	62.6			1, 16.8	257.72	4	1	1.93
**C3-14**	38.8	56.4	78.4		28.5	243.76	2	1	2.66
**C3-15**	35.7		86.9		5.1	257.78	2	1	2.82
**C3-16**	23.7	45.4	83.1		28.7	240.73	3	1	2.03
**C3-17**	57.6	39.3	61.9		7.4	287.96	3	2	2.89
**C3-18**	0.3	37.5	0.6			247.68	3	1	2.57
**C3-19**	59.2	65.2	99.5	0.508		300.74	6	2	2.01
**C3-20**	3.9	32.9	42.7		29.1	268.74	4	2	1.98
**C3-21**	58.4	27.6	81.3		9.9	291.54	4	2	1.55
**C3-22**	-2.4	-12.1	10.4			302.72	7	2	0.68
**C3-23**	-4.1	-11.7	-3.5		3.7	272.75	4	2	1.81
**C3-24**	29.2	58.8	72.3			268.74	4	2	1.61
**C3-25**	36.2	57.1	82.4		64.5, 67.8	262.64	4	2	1.29

**C3-26**	68.2	42.2	69.5			382.89	5	2	3.38
**C3-27**	63.4	63.8	54.2	1.65		389.13	4	3	3.53
**C3-28**	15.1	44.1	42.0		48.5, 48.7	231.75	2	1	2.21
**C3-29**	46.7	88.9	73.0		89.7	237.69	3	1	2.15
**C3-30**	10.6	14.5	83.1			291.78	3	1	2.01
**C3-31**	-13.7	23.0	0.3			272.82	3	1	2.83
**C3-32**	23.6	53.1	12.5			342.78	5	1	2.02
**C3-33**	16.6	55.3	36.3		24.7, 38.5	255.79	2	1	3.52
**C3-34**	50.9	41.7	78.6		-2.4	255.79	2	1	3.88

Supplementary Table 5. Screening data for Cluster-4 - Non-cyclized 3^0-chloroacetamide

	GSTO binding assay (% Inhibition at 10 μM)		GSTO substrate assay		% Inhibition of HCT116p53+/+ cell proliferation at 10 μM	MW	HBA	HBD	LogP
	Recombinant	Endogenous	% Inhibition at 10 μM	IC_{50} (μM)					
**C4-1**	40.4	58.5	73.0	3.80	76.2, 81.4	286.08	2	0	2.97
**C4-2**	53.2	52.8	78.6	1.07	84.1, 93.6	290.67	3	0	2.29
**C4-3**	-1.7	32.3			70.6, 71.9	240.69	4	1	0.72
**C4-4**	5.9	17.0	-0.8		-11.3, 5.3	239.72	4	0	-0.42
**C4-5**	-8.2	15.9	5.0		9.9, 16.9	361.68	3	0	4.52
**C4-6**	55.2	49.5	29.3		44.1, 68.5	277.75	3	0	3.27
**C4-7**	-36.4	53.0	1.8		81.8	309.58	4	1	2.21
**C4-8**	59.5	53.0	97.2	0.237	70.7	255.70	4	0	1.42
**C4-9**	66.7	43.5	20.7		-6.8, 5.4	262.14	3	0	2.39
**C4-10**	66.4	53.1	99.5	1.95	82.1	263.73	3	0	1.7
**C4-11**	60.0	51.8	103.4	2.36	2.8	255.70	4	0	1.72
**C4-12**	-4.7	-29.3	81.7		91.1, 92.6	234.68	5	0	-0.76
**C4-13**	5.1	7.3	27.2		62.3, 67.3	296.77	6	1	-1.03
**C4-14**	45.2	56.8	89.1		70.3, 75	254.72	4	1	0.69
**C4-15**	65.8	34.0	98.2		-6.8, -5.5	263.73	3	0	2.17
**C4-16**	66.3	30.0	32.6		86.3, 89.8	330.84	3	0	3.8
**C4-17**	69.3		70.2	0.902	29	373.84	5	0	3.42
**C4-18**	4.5		18.7		14.2	366.81	6	2	0.65
**C4-19**	2.1	-20.7	11.8		67.3	330.82	5	2	1.54
**C4-20**	9.3	27.6	-7.3		10.4, 6.3	509.73	9	1	0.67
**C4-21**	9.8	20.6	23.3		50.6, 54.9	428.96	5	2	3.03

Supplementary Table 6. Screening data for Cluster-5 - Cyclized 3^0-chloroacetamide

	GSTO binding assay (% Inhibition at 10 μM)		GSTO substrate assay		% Inhibition of HCT116p53+/+ cell proliferation at 10 μM	MW	HBA	HBD	LogP
	Recombinant	Endogenous	% Inhibition at 10 μM	IC_{50} (μM)					
**C5-1**	67.5		83.5	0.048	93.4	251.76	2	0	3.13
**C5-2**	52.5	66.2	58.2	0.559	40.2, 61.1	243.74	4	0	0.61
**C5-3**	9.4	61.5	59.8		28.2	256.71	3	0	1.76
**C5-4**	13.5	33.4	41.0		81.9, 84.4	254.72	4	1	0.92
**C5-5**	44.2	51.5	78.6		-16.3	268.74	4	0	1.67
**C5-6**	70.2		94.6	0.191	56.2	342.15	4	0	2.46
**C5-7**	65.1	53.1	72.8	0.338	-2.0, -4.5	270.74	3	0	1.9
**C5-8**	62.3	75.7	93.8	0.293	82.6, 87.3	277.76	4	0	1.07
**C5-9**	62.3	52.8	83.7	0.381	68.4, 79.3	302.81	3	0	2.95
**C5-10**	59.8	55.8	68.3	0.411	71.5, 90.4	337.67	3	0	2.39
**C5-11**	57.7	62.9	5.7		19.6	283.78	4	0	0.87
**C5-12**	66.0	47.1	84.9		7.3	330.84	5	0	1.06
**C5-13**	61.1	66.2	98.1	0.156	81.1, 55.9	327.79	6	0	0.26
**C5-14**	61.2	60.9	91.8	0.496	28.2, 11.4	338.76	5	0	1.21
**C5-15**	62.4	63.7		0.262	82.7, 87.3	360.82	7	0	0.34
**C5-16**	82.7	61.7	2.4		48.3, 46.5	328.82	5	0	0.71
**C5-17**	9.4		69.5			269.73	4	0	1.81
**C5-18**	69.7	72.7		0.164	91.6, 91.4	316.81	5	0	1.37
**C5-19**	72.2	40.0			84.6, 75.2	240.65	4	0	1.8
**C5-20**	68.1	61.0		2.29	80.5, 89.8	288.75	5	1	0.8
**C5-21**	65.0	28.9	40.0		78.2, 83.6	388.85	6	0	3.87
**C5-22**	68.6	22.7	1.0		41.2	294.76	4	0	2.49
**C5-23**	67.2		16.6		42.2	433.77	3	0	6.01
**C5-24**	54.9	69.2	83.7	4.11	79.8, 87.3	254.67	5	1	0.15

Supplementary Table 7. Inhibitory activities of selected GSTO1 inhibitors

Cpd	Structure	GSTO1 Substrate assay	Competitive Binding assay	PDI Assay[c]	Fold selectivity
			Endogenous GSTO1		
		IC$_{50}$ (μM)[a]	IC$_{50}$ (nM)	IC$_{50}$ (μM)	
**4a**	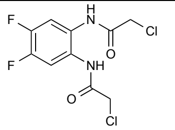	3.14 ± 0.27	3900	6.04 ± 0.62	1.9
**C1-24**	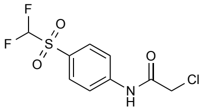	0.148 ± 0.34	N.T.[b]	3.57 ± 0.69	24.1
**C1-27**	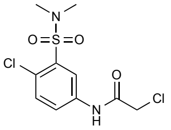	0.031 ± 0.006	21	3.13 ± 0.52	101
**C1-28**	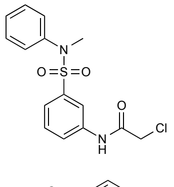	0.064 ± 0.016	8	1.25 ± 0.23	19.5
**C1-29**	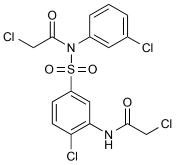	0.030 ± 0.005	44	1.46 ± 0.33	48.7
**C1-31**	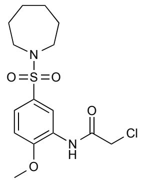	0.221 ± 0.077	7	3.60 ± 0.63	16.3
**C2-10**	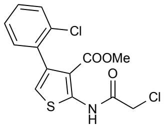	0.062 ± 0.010	190	> 10	161.3
**C2-16**	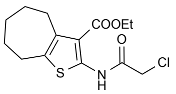	0.066 ± 0.013	520	4.03 ± 0.72	61.1
**C2-18**	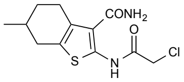	0.119 ± 0.027	75	> 10	84
**C2-22**	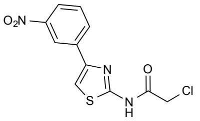	0.136 ± 0.018	N.T.	0.39 ± 0.05	2.9

**C2-26**	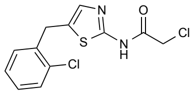	0.125 ± 0.039	N.T.	0.09 ± 0.01	0.7

[a] IC_{50} values are reported as mean ± SEM calculated from at least 3 independent experiments.
[b] N.T. – Not tested
[c] Inhibition of PDI-catalyzed reduction of Insulin.

Supplementary Table 8. GSTO1 expression in Oncomine gene expression studies.

Cancer	Cancer subtype	Data set	Year	Number of samples	Fold change in expression	p-value
Head and neck	Tongue squamous cell carcinoma	Talbot Lung	2005	93	1.969	1.23E-7
		Estilo Head-Neck	2009	58	2.015	1.86E-6
		Ye Head-Neck	2008	38	1.458	0.008
	Head and neck squamous cell carcinoma	Cromer Head-Neck	2004	38	1.722	0.008
		Ginos Head-Neck	2004	54	1.724	1.59E-7
Colorectal	Colon adenocarcinoma	Kaiser Colon	2007	105	1.457	0.001
		TCGA Colorectal	2011	237	1.456	3.03E-7
	Colon mucinous adenocarcinoma	Kaiser Colon	2007	105	1.521	5.97E-4
		TCGA Colorectal	2011	237	1.635	3.18E-6
	Cecum adenocarcinoma	Kaiser Colon	2007	105	1.547	9.10E-4
		TCGA Colorectal	2011	237	1.365	2.61E-4
	Colorectal carcinoma	Skrzypczak Colorectal	2010	105	1.575	7.76E-6
		Hong Colorectal	2010	82	1.408	9.05E-5
	Rectal adenocarcinoma	Kaiser Colon	2007	105	1.496	0.002
		TCGA Colorectal	2011	237	1.331	1.14E-4
Esophageal	Esophageal adenocarcinoma	Wang Esophagus	2006	52	2.146	7.18E-8
		Kimchi Esophagus	2005	24	1.608	0.011
		Hao Esophagus	2006	48	2.148	0.033
	Esophageal squamous cell carcinoma	Su Esophagus 2	2011	106	1.764	6.13E-15
		Hu Esophagus	2010	34	1.521	2.47E-4
Lymphoma	Diffuse large B-cell lymphoma	Rosenwald Multi-cancer	2001	102	2.125	4.13E-4
		Rosenwald Lymphoma	2002	293	1.482	4.57E-4
		Brune Lymphoma	2008	67	3.815	1.83E-7
		Compagno Lymphoma	2009	136	1.881	3.21E-14
		Basso Lymphoma	2005	336	1.251	0.011
	Activated B-cell like Diffuse B-cell lymphoma	Alizadeh Lymphoma	2000	120	1.813	8.44E-6
		Compagno Lymphoma	2009	136	1.390	0.003
	Germinal center B-cell like Diffuse B-cell lymphoma	Alizadeh Lymphoma	2000	120	2.148	1.85E-7
		Compagno Lymphoma	2009	136	1.918	1.07E-7
Melanoma	Cutaneous melanoma	Riker Melanoma	2008	87	3.831	5.24E-5
		Talantov Melanoma	2005	70	4.084	2.81E-7

Criteria: Number of studies > 1, p-value<0.05. Green = Significant overexpression of GSTO1 and IL-1β

Supplementary Table 9. Cytotoxicity of selected GSTO1 inhibitors in a panel of cancer cell lines

**Cpd**	**GI_{50}[a] (μM)**			
	HT29	HCT116	H460	$NCI^{ADR\ Res}$
**4a**	5.6 ± 0.5	2.4 ± 0.2	3.5	2.2
**C1-24**	5.1 ± 1.3	3.3 ± 1.1	N.T.[b]	N.T.
**C1-27**	4.3 ± 0.6	1.2 ± 0.6	3.3	3.7
**C1-28**	2.7 ± 0.5	2.2 ± 0.7	4.5	3.8
**C1-29**	15.5 ± 0.2	13.7 ± 1.3	N.T.	N.T.
**C1-31**	8.7 ± 1.2	9.1 ± 1.1	>20	>20
**C2-10**	18.1 ± 1.6	18.8 ± 1.6	N.T.	N.T.
**C2-16**	19.8	>20	N.T.	N.T.
**C2-18**	9.5 ± 2.8	6.9 ± 1.9	N.T.	N.T.
**C2-22**	10.9 ± 1.6	7.4 ± 1.9	>20	N.T.
**C2-26**	14.2	6.2 ± 1.8	>20	14.6

[a] GI_{50} is the drug concentration that causes a 50% reduction in cell proliferation.
GI_{50} values are reported as mean ± SEM calculated from at least 3 independent experiments.
[b] N.T. – Not tested

Supplementary Table 10. List of top 20 genes up-regulated following GSTO1 siRNA treatment

Gene Symbol	Gene Name (Description)	Fold change
CHAC1	ChaC, cation transport regulator homolog 1 (E. coli)	22.785
VGF	VGF nerve growth factor inducible	12.389
DDIT4	DNA-damage-inducible transcript 4	10.486
GDF15	Growth differentiation factor 15	7.823
GALNT4	UDP-N-acetyl-alpha-D-galactosamine:polypeptide N-acetylgalactosaminyltransferase 4 (GalNAc-T4)	6.835
TUBA1A	Tubulin, alpha 1a	6.195
SLC7A11	Solute carrier family 7, (cationic amino acid transporter, y+ system) member 11	6.175
BEX2	Brain expressed X-linked 2	5.670
CDKN1A	Cyclin-dependent kinase inhibitor 1A (p21, Cip1)	5.507
DPM3	Dolichyl-phosphate mannosyltransferase polypeptide 3	5.127
HIST1H3E	Histone cluster 1, H3e	5.127
ASNS	Asparagine synthetase	5.021
ARID5B	AT rich interactive domain 5B (MRF1-like)	4.262
LINC00263	Oligodendrocyte maturation-associated long intergenic non-coding RNA	4.134
INSIG1	Insulin induced gene 1	4.127
S100A14	S100 calcium binding protein A14	3.878
SCD	Stearoyl-CoA desaturase (delta-9-desaturase)	3.864
ELF3	E74-like factor 3 (ets domain transcription factor, epithelial-specific)	3.811
BBC3	BCL2 binding component 3	3.775
FADS1	Fatty acid desaturase 1	3.766

Supplementary Table 11. List of top 20 genes down-regulated following GSTO1 siRNA treatment

Gene Symbol	Gene Name (Description)	Fold change
F3	Coagulation factor III (thromboplastin, tissue factor)	0.125
CPA4	Carboxypeptidase A4	0.148
HIST1H4J	Histone cluster 1, H4IJ	0.155
LOC100144603	Hypothetical transcript	0.160
LOC730755	Keratin associated protein 2-1; keratin associated protein 2-4; keratin associated protein 2-3; similar to keratin associated protein 2-4; keratin associated protein 2-2	0.160
MTVR2	Mouse mammary tumor virus receptor homolog 2	0.160
THBS1	Thrombospondin 1	0.167
LY6G5B	Lymphocyte antigen 6 complex, locus G5B; casein kinase 2, beta polypeptide	0.223
CYR61	Cysteine-rich, angiogenic inducer, 61	0.237
GALNT5	UDP-N-acetyl-alpha-D-galactosamine:polypeptide N-acetylgalactosaminyltransferase 5 (GalNAc-T5)	0.260
AMOTL2	Angiomotin like 2	0.283
HIST1H3A	Histone cluster 1, H3a	0.296
GJB3	Gap junction protein, beta 3, 31kDa	0.303
PDLIM2	PDZ and LIM domain 2 (mystique)	0.328
S100A10	S100 calcium binding protein A10	0.335
BCS1L	BCS1-like (yeast)	0.347
ALDH1A3	Aldehyde dehydrogenase 1 family, member A3	0.348
NT5E	5'-Nucleotidase, ecto (CD73)	0.352
WNT10B	Wingless-type MMTV integration site family, member 10B	0.353
BCAR3	Breast cancer anti-estrogen resistance 3	0.358

Supplementary Table 12. Top 20 gene sets for up-regulated genes of GSTO1 siRNA Bru-Seq data obtained by GSEA

Gene set	Size	ES	NES
KRIGE_RESPONSE_TO_TOSEDOSTAT_24HR_UP	413	0.581	3.006
CHANG_CORE_SERUM_RESPONSE_DN	84	0.695	2.904
KRIGE_AMINO_ACID_DEPRIVATION	20	0.914	2.824
RUIZ_TNC_TARGETS_UP	48	0.734	2.798
PODAR_RESPONSE_TO_ADAPHOSTIN_UP	101	0.634	2.761
WANG_RESPONSE_TO_GSK3_INHIBITOR_SB216763_UP	140	0.553	2.548
PACHER_TARGETS_OF_IGF1_AND_IGF2_UP	19	0.856	2.544
CUI_GLUCOSE_DEPRIVATION	31	0.746	2.541
CSR_LATE_UP.V1_DN	39	0.708	2.538
RODRIGUES_DCC_TARGETS_DN	55	0.645	2.505
ALK_DN.V1_UP	21	0.775	2.470
WARTERS_RESPONSE_TO_IR_SKIN	37	0.697	2.464
PENG_LEUCINE_DEPRIVATION_UP	64	0.603	2.429
SMIRNOV_RESPONSE_TO_IR_6HR_UP	87	0.585	2.428
GARY_CD5_TARGETS_UP	255	0.491	2.421
GAJATE_RESPONSE_TO_TRABECTEDIN_UP	19	0.801	2.388
HELLER_SILENCED_BY_METHYLATION_DN	52	0.626	2.366
KARLSSON_TGFB1_TARGETS_DN	106	0.531	2.362
ZHANG_TLX_TARGETS_DN	34	0.677	2.350
MTOR_UP.N4.V1_UP	109	0.530	2.348

Supplementary Table 13. Top 20 gene sets for down-regulated genes of GSTO1 siRNA Bru-Seq data obtained by GSEA

Gene set	Size	ES	NES
BURTON_ADIPOGENESIS_PEAK_AT_16HR	33	-0.747	-2.424
RUIZ_TNC_TARGETS_DN	114	-0.615	-2.363
REACTOME_ACTIVATION_OF_THE_PRE_REPLICATIVE_COMPLEX	27	-0.700	-2.206
MANALO_HYPOXIA_DN	242	-0.587	-2.204
VERNELL_RETINOBLASTOMA_PATHWAY_DN	16	-0.780	-2.151
FRASOR_RESPONSE_TO_SERM_OR_FULVESTRANT_DN	39	-0.630	-2.147
REACTOME_DNA_STRAND_ELONGATION	27	-0.689	-2.132
GRAHAM_NORMAL_QUIESCENT_VS_NORMAL_DIVIDING_DN	69	-0.588	-2.128
GSE36476_CTRL_VS_TSST_ACT_40H_MEMORY_CD4_TCELL_YOUNG_DN	149	-0.557	-2.119
KEGG_DNA_REPLICATION	32	-0.665	-2.106
CROONQUIST_IL6_DEPRIVATION_DN	77	-0.578	-2.101
STEIN_ESRRA_TARGETS_RESPONSIVE_TO_ESTROGEN_DN	31	-0.672	-2.089
SCHLOSSER_MYC_TARGETS_AND_SERUM_RESPONSE_DN	40	-0.622	-2.086
FUJII_YBX1_TARGETS_DN	148	-0.565	-2.086
MYC_UP.V1_UP	70	-0.584	-2.076
GNF2_MCM5	60	-0.586	-2.061
SCHUHMACHER_MYC_TARGETS_UP	62	-0.577	-2.049
REACTOME_ACTIVATION_OF_ATR_IN_RESPONSE_TO_REPLICATION_STRESS	33	-0.630	-2.027
SUNG_METASTASIS_STROMA_DN	39	-0.611	-2.023
SONG_TARGETS_OF_IE86_CMV_PROTEIN	53	-0.586	-2.014

Supplementary Table 14. List of top 20 genes up-regulated following **C1-27** 4 h treatment

**Gene Symbol**	**Gene Name (Description)**	**Fold change**
OSGIN1	Oxidative stress induced growth inhibitor 1	3.57
HMOX1	Heme oxygenase (decycling) 1	2.33
SLC7A11	Solute carrier family 7, (cationic amino acid transporter, y+ system) member 11	2.19
CYP4F11	Cytochrome P450, family 4, subfamily F, polypeptide 11	2.10
GCLM	Glutamate-cysteine ligase, modifier subunit	1.97
ABCB6	ATP-binding cassette, sub-family B (MDR/TAP), member 6	1.81
TXNRD1	Thioredoxin reductase 1; hypothetical LOC100130902	1.78
SRXN1	Sulfiredoxin 1 homolog (S. cerevisiae)	1.76
GCLC	Glutamate-cysteine ligase, catalytic subunit	1.76
HIST1H3E	Histone cluster 1, H3j	1.74
GLA	Galactosidase, alpha	1.69
GPANK1	G-patch domain and ankyrin repeats 1	1.69
GSR	Glutathione reductase	1.68
CDC14C	Cell division cycle 14C	1.67
PIR	Pirin	1.66
EID3	EP300 interacting inhibitor of differentiation 3	1.66
G6PD	Glucose-6-phosphate dehydrogenase	1.65
SDHAF1	Succinate dehydrogenase complex assembly factor 1	1.60
DNAJB9	DnaJ heat shock protein family (HSp40) member B9	1.56
LY6G5B	Lymphocyte antigen 6 complex, locus G5B	1.50

Supplementary Table 15. List of top 20 genes down-regulated following **C1-27** 4 h treatment

Gene Symbol	Gene Name (Description)	Fold change
MRPL41	Mitochondrial ribosomal protein L41	0.42
NDNL2	Necdin-like 2	0.50
GTF2H2D	General transcription factor IIH, polypeptide 2, 44kDa	0.56
MT1X	Metallothionein 1X	0.59
RAD51-AS1	RAD51 antisense RNA 1	0.64
HMGB3P1	High mobility group box 3 pseudogene 1	0.66
MRPL42P5	Mitochondrial ribosomal protein L42 pseudogene 5	0.66
LOC100287042	Hypothetical protein LOC100287042	0.66
RGS2	Regulator of G-protein signaling 2, 24kDa	0.67
DDIT4	DNA damage inducible transcript 4	0.67
METTL18	Methyltransferase like 18	0.67
ID3	Inhibitor of DNA binding 3, dominant negative helix-loop-helix protein	0.67
ALG10	Asparagine-linked glycosylation 10, alpha-1,2-glucosyltransferase homolog (S. pombe)	0.68
CST6	Cystatin E/M	0.69
TAF10	TATA-box binding protein associated factor 10	0.69
TAS2R14	Taste 2 receptor member 14	0.69
JUNB	Jun B proto-oncogene, AP-1 transcription factor subunit	0.70
NKX3-1	NK3 homebox 1	0.70
C14orf142	Chromosome 14 open reading frame 142	0.70
PIGC	Phosphatidylinositol glycan anchor biosynthesis class C	0.71

Supplementary Table 16. List of top 20 genes up-regulated following **C1-27** 24 h treatment

Gene Symbol	Gene Name (Description)	Fold change
DHRS3	Dehydrogenase/reductase (SDR family) member 3	4.70
OSGIN1	Oxidative stress induced growth inhibitor 1	3.96
GDF15	Growth differentiation factor 15	3.64
LINC00263	OLMALINC, Oligodendrocyte maturation-associated long intragenic non-coding RNA	3.15
INSIG1	Insulin induced gene 1	3.07
SLC7A11	Solute carrier family 7, (cationic amino acid transporter, y+ system) member 11	3.05
IDH1	Isocitrate dehydrogenase 1 (NADP+), soluble	2.99
FADS1	Fatty acid desaturase 1	2.86
FDFT1	Farnesyl-diphosphate farnesyltransferase 1	2.85
STARD4	StAR-related lipid transfer (START) domain containing 4	2.64
CYP4F11	Cytochrome P450, family 4, subfamily F, polypeptide 11	2.61
EFNA4	Ephrin-A4	2.61
LPIN1	Lipin 1	2.55
FGF18	Fibroblast growth factor 18	2.48
EGR1	Early growth response 1	2.35
G6PD	Glucose-6-phosphate dehydrogenase	2.31
DDIT3	DNA damage inducible transcript 3	2.27
RPL23AP32	Ribosomal protein L32a pseudogene 2	2.18
HSD17B13	Hydroxysteroid 17-beta dehydrogenase 13	2.17
PHF21A	PHD finger protein 21A	2.11

Supplementary Table 17. List of top 20 genes down-regulated following **C1-27** 24 h treatment

Gene Symbol	Gene Name (Description)	Fold change
GALNT5	UDP-N-acetyl-alpha-D-galactosamine:polypeptide N-acetylgalactosaminyltransferase 5 (GalNAc-T5)	0.17
F3	Coagulation factor III (thromboplastin, tissue factor)	0.22
CTGF	Connective tissue growth factor	0.28
CYR61	Cysteine-rich, angiogenic inducer, 61	0.29
THBS1	Thrombospondin 1	0.29
HIST3H2A	histone cluster 3, H2a	0.31
MRPL41	Mitochondrial ribosomal protein L41	0.33
LOC100506939	Keratin associated protein 2-1	0.34
GPR110	G protein-coupled receptor 110	0.35
HIST2H2AB	histone cluster 2, H2ab	0.37
TERC	telomerase RNA component	0.38
NT5E	5'-nucleotidase, ecto (CD73)	0.39
PLK2	Polo-like kinase 2 (Drosophila)	0.40
LAMB3	Laminin, beta 3	0.40
SEMA3C	Sema domain, immunoglobulin domain (Ig), short basic domain, secreted, (semaphorin) 3C	0.41
ALDH1A3	Aldehyde dehydrogenase 1 family, member A3	0.42
CPA4	Carboxypeptidase A4	0.42
GDA	Guanine deaminase	0.42
ITGB8	Integrin, beta 8	0.43
SLCO1B3	Solute carrier organic anion transporter family member 1B3	0.43

Supplementary Table 18. List of top 20 genes up-regulated following **C1-14** 4 h treatment

Gene Symbol	Gene Name (Description)	Fold change
HIST1H4K	Histone cluster 1, H4l	18.57
RPPH1	Ribonuclease P RNA component H1	16.74
HIST1H2BL	Histone cluster 1, H2bl	15.96
HIST1H2AJ	Histone cluster 1, H2aj	11.27
HIST1H2AG	Histone cluster 1, H2ag	10.36
HFM1	HFM1, ATP-dependent DNA helicase homolog (S. cerevisiae)	8.78
HIST1H1E	Histone cluster 1, H1e	8.67
HIST1H2AI	Histone cluster 1, H2ag	8.25
HIST4H4	Histone cluster 1, H4l	7.84
HIST1H4D	Histone cluster 1, H4l	7.81
HIST2H3D	Histone cluster 1, H3j	7.39
HIST3H2BB	Histone cluster 3, H2bb	6.97
HIST1H3H	Histone cluster 1, H3j	6.41
HIST1H2AH	Histone cluster 1, H2ag	6.00
HIST1H2BN	Histone cluster 1, H2bn	5.80
HIST1H4C	Histone cluster 1, H4l	5.72
HIST1H2AC	Histone cluster 1, H2ac	5.52
HIST1H2BJ	Histone cluster 1, H2bj	5.45
SCARNA10	Small Cajal body-specific RNA 10	5.37
HIST2H2AB	Histone cluster 2, H2ab	5.21

Supplementary Table 19. List of top 20 genes down-regulated following **C1-14** 4 h treatment

Gene Symbol	Gene Name (Description)	Fold change
SLC7A5P1	Solute carrier family 7 (amino acid transporter light chain, L system), member 5 pseudogene 1	0.38
HUS1B	HUS1 checkpoint clamp component B	0.46
ZNF670-ZNF695	ZNF670-ZNF695 readthrough (NMD candidate)	0.58
BACE1-AS	BACE1 antisense RNA	0.60
LINC00311	Long intergenic non-protein coding RNA 311	0.62
LINC00115	Long intergenic non-protein coding RNA 115	0.64
TYRO3P	TYRO3P protein tyrosine kinase pseudogene	0.65
P2RX5-TAX1BP3	P2RX5-TAX1BP3 readthrough (NMD candidate)	0.65
LINC00471	Long intergenic non-protein coding RNA 471	0.67
AURKAPS1	Aurora kinase A pseudogene 1	0.68
RPA4	Replication protein A4	0.69
RIBC2	RIB43A domain with coiled-coils 2	0.70
REP15	RAB15 effector protein	0.70
ZNF572	Zinc finger protein 572	0.70
ATP5L2	ATP synthase, H+ transporting, mitochondrial Fo complex subunit G2	0.70
TNFAIP3	TNF alpha induced protein 3	0.71
ATP6AP1L	ATPase, H+ transporting, lysosomal accessory protein 1-like	0.71
TIGD6	Tigger transposable element derived 6	0.71
IER3	Immediate early response 3	0.72
HSP90AB4P	Heat Shock Protein 90kDa alpha family class B member 4, pseudogene	0.72

Supplementary Table 20. Top 12 gene sets for up-regulated genes of **C1-27**-4h treatment Bru-Seq data obtained by GSEA

Gene set	Size	NES	FDR q-val
NFE2L2.V2	115	2.67	< E-05
HOUSTIS_ROS	20	2.38	< E-05
TIEN_INTESTINE_PROBIOTICS_24HR_DN	165	2.25	6.26E-04
MORF_UBE2I	222	2.23	5.22E-04
REACTOME_ER_PHAGOSOME_PATHWAY	48	2.23	5.63E-04
REACTOME_REGULATION_OF_ORNITHINE_DECARBOXYLASE_ODC	43	2.22	5.12E-04
CHR19Q13	144	2.22	4.69E-04
REACTOME_CDK_MEDIATED_PHOSPHORYLATION_AND_REMOVAL_OF_CDC6	42	2.19	4.70E-04
REACTOME_TRANSLATION	137	2.19	4.38E-04
PECE_MAMMARY_STEM_CELL_DN	98	2.18	3.87E-04
HSIAO_HOUSEKEEPING_GENES	329	2.17	6.78E-04
REACTOME_VIF_MEDIATED_DEGRADATION_OF_APOBEC3G	43	2.16	7.41E-04

Supplementary Table 21. Top 20 gene sets for down-regulated genes of **C1-27**-4h treatment Bru-Seq data obtained by GSEA

Gene set	Size	NES	FDR q-val
MIYAGAWA_TARGETS_OF_EWSR1_ETS_FUSIONS_DN	75	-2.46	1.10E-03
HOELZEL_NF1_TARGETS_UP	37	-2.37	1.11E-03
RAF_UP.V1_DN	69	-2.32	1.11E-03
VERHAAK_AML_WITH_NPM1_MUTATED_DN	45	-2.29	2.51E-03
ZHENG_FOXP3_TARGETS_IN_THYMUS_UP	120	-2.26	2.68E-03
SWEET_LUNG_CANCER_KRAS_DN	100	-2.25	2.60E-03
MEISSNER_BRAIN_HCP_WITH_H3K4ME3_AND_H3K27ME3	158	-2.24	3.17E-03
CUI_TCF21_TARGETS_2_DN	379	-2.21	4.16E-03
GSE17974_IL4_AND_ANTI_IL12_VS_UNTREATED_6H_ACT_CD4_TCELL_DN	77	-2.19	5.06E-03
VECCHI_GASTRIC_CANCER_ADVANCED_VS_EARLY_UP	34	-2.19	4.55E-03
SMID_BREAST_CANCER_LUMINAL_B_DN	97	-2.19	4.14E-03
HUANG_DASATINIB_RESISTANCE_UP	42	-2.18	4.71E-03
HUTTMANN_B_CLL_POOR_SURVIVAL_DN	23	-2.17	4.95E-03
CHARAFE_BREAST_CANCER_LUMINAL_VS_BASAL_DN	228	-2.16	5.00E-03
LEF1_UP.V1_DN	32	-2.15	5.11E-03
GARGALOVIC_RESPONSE_TO_OXIDIZED_PHOSPHOLIPIDS_GREY_DN	19	-2.15	4.86E-03
MODULE_12	70	-2.15	5.29E-03
MARSON_FOXP3_TARGETS_DN	24	-2.14	5.12E-03
DUTERTRE_ESTRADIOL_RESPONSE_24HR_DN	201	-2.13	6.36E-03
GABRIELY_MIR21_TARGETS	185	-2.11	7.60E-03

Supplementary Table 22. Top 20 gene sets for up-regulated genes of **C1-27**-24h treatment Bru-Seq data obtained by GSEA

Gene set	Size	NES	FDR q-val
CHANG_CORE_SERUM_RESPONSE_DN	80	2.46	< E-05
ONGUSAHA_TP53_TARGETS	16	2.43	< E-05
NFE2L2.V2	118	2.39	< E-05
CSR_LATE_UP.V1_DN	39	2.37	< E-05
TIEN_INTESTINE_PROBIOTICS_24HR_DN	167	2.37	< E-05
SCHMIDT_POR_TARGETS_IN_LIMB_BUD_UP	15	2.34	1.42E-04
KRIGE_AMINO_ACID_DEPRIVATION	19	2.26	3.63E-04
ADDYA_ERYTHROID_DIFFERENTIATION_BY_HEMIN	38	2.24	7.38E-04
PACHER_TARGETS_OF_IGF1_AND_IGF2_UP	19	2.20	1.22E-03
ZHANG_TLX_TARGETS_60HR_UP	100	2.15	2.44E-03
PODAR_RESPONSE_TO_ADAPHOSTIN_UP	97	2.13	3.44E-03
MODULE_93	81	2.13	3.36E-03
WENG_POR_TARGETS_LIVER_UP	20	2.12	3.55E-03
ZHANG_TLX_TARGETS_DN	35	2.11	3.77E-03
ZHANG_TLX_TARGETS_36HR_UP	82	2.11	3.63E-03
WILCOX_PRESPONSE_TO_ROGESTERONE_UP	83	2.11	3.51E-03
HELLER_SILENCED_BY_METHYLATION_DN	50	2.10	3.95E-03
PELLICCIOTTA_HDAC_IN_ANTIGEN_PRESENTATION_UP	55	2.08	5.32E-03
OXIDOREDUCTASE_ACTIVITY	109	2.07	5.70E-03
JI_RESPONSE_TO_FSH_UP	26	2.03	1.15E-02

Supplementary Table 23. Top 20 gene sets for down-regulated genes of **C1-27**-24h treatment Bru-Seq data obtained by GSEA

**Gene set**	**Size**	**NES**	**FDR q-val**
ZWANG_CLASS_3_TRANSIENTLY_INDUCED_BY_EGF	104	-2.59	< E-05
RUIZ_TNC_TARGETS_DN	113	-2.52	< E-05
KIM_WT1_TARGETS_12HR_DN	144	-2.52	< E-05
MIYAGAWA_TARGETS_OF_EWSR1_ETS_FUSIONS_DN	75	-2.51	< E-05
KIM_WT1_TARGETS_UP	110	-2.49	< E-05
MEISSNER_BRAIN_HCP_WITH_H3K4ME3_AND_H3K27ME3	161	-2.49	< E-05
PEDERSEN_TARGETS_OF_611CTF_ISOFORM_OF_ERBB2	35	-2.48	< E-05
ONDER_CDH1_TARGETS_2_DN	59	-2.42	< E-05
CHARAFE_BREAST_CANCER_LUMINAL_VS_BASAL_DN	232	-2.39	< E-05
PLASARI_TGFB1_TARGETS_10HR_UP	56	-2.38	< E-05
MODULE_64	43	-2.37	< E-05
AMIT_SERUM_RESPONSE_120_MCF10A	38	-2.37	< E-05
ESC_J1_UP_LATE.V1_UP	37	-2.36	< E-05
P53_DN.V1_UP	57	-2.34	< E-05
BROWNE_HCMV_INFECTION_2HR_DN	27	-2.31	< E-05
WINZEN_DEGRADED_VIA_KHSRP	34	-2.30	< E-05
ONDER_CDH1_TARGETS_1_DN	96	-2.30	< E-05
WU_CELL_MIGRATION	49	-2.28	1.27E-04
LIN_SILENCED_BY_TUMOR_MICROENVIRONMENT	16	-2.28	1.20E-04
PASINI_SUZ12_TARGETS_DN	157	-2.26	1.73E-04

Supplementary Table 24. Top 20 gene sets for up-regulated genes of **C1-14**-4h treatment Bru-Seq data obtained by GSEA

Gene set	Size	NES	FDR q-val
MORF_TPT1	98	2.06	< E -05
STRUCTURAL_CONSTITUENT_OF_RIBOSOME	72	2.05	< E -05
MORF_ACTG1	132	2.05	< E -05
REACTOME_PEPTIDE_CHAIN_ELONGATION	80	2.03	< E -05
KEGG_RIBOSOME	82	2.02	< E -05
MORF_JUND	62	2.01	1.47E-04
BILANGES_SERUM_AND_RAPAMYCIN_SENSITIVE_GENES	59	2.01	1.26E-04
MORF_NPM1	157	2.01	1.11E-04
MORF_NME2	142	2.00	9.83E-05
REACTOME_SRP_DEPENDENT_COTRANSLATIONAL_PROTEIN_ TARGETING_TO_MEMBRANE	102	2.00	8.85E-05
REACTOME_NONSENSE_MEDIATED_DECAY_ENHANCED_BY_THE_ EXON_JUNCTION_COMPLEX	100	1.99	2.41E-04
REACTOME_INFLUENZA_VIRAL_RNA_TRANSCRIPTION_AND_ REPLICATION	93	1.98	2.95E-04
GCM_TPT1	67	1.98	2.72E-04
REACTOME_3_UTR_MEDIATED_TRANSLATIONAL_REGULATION	99	1.98	2.53E-04
LI_AMPLIFIED_IN_LUNG_CANCER	104	1.98	2.36E-04
GNATENKO_PLATELET_SIGNATURE	29	1.96	2.76E-04
GCM_PFN1	45	1.96	2.60E-04
GCM_NPM1	111	1.95	4.91E-04
REACTOME_INFLUENZA_LIFE_CYCLE	122	1.94	5.58E-04
MUNSHI_MULTIPLE_MYELOMA_UP	42	1.94	5.74E-04

Supplementary Table 25. Effect of **C1-27** treatment in a panel of cancer cell lines.

Cancer type	Cell line	GI_{50} (μM)[a]			RAS status
		**C1-27**	Erastin	Piperlongumine	
Colon cancer	HT29	4.3 ± 0.6	42.8 ± 3.8	5.1 ± 0.5	Wild type
	HCT116	1.2 ± 0.6	15.9 ± 0.7	2.6 ± 0.2	KRAS Mutant
Pancreatic cancer	BxPC3	5.5 ± 1.0	> 50	6.8 ± 1.4	Wild type
	Panc-1	1.8 ± 0.1	0.5 ± 0.2	4.9 ± 0.8	KRAS Mutant
Lung cancer	A549	11.7	>50	16.9	KRAS Mutant
	H1299	1.9	1.2	2.9	NRAS mutant

[a] GI_{50} is the drug concentration that causes a 50% reduction in cell proliferation. GI_{50} values are reported as mean ± SEM calculated from at least 3 independent experiments.

Supplementary Table 26. Summary of antibody source and dilutions

Antibody	Commercial source	Catalogue No.	Dilution
GSTO1	GeneTex	GTX105655	1:1000
HMOX1	Cell Signaling	# 5061	1:1000
SLC7A11	Cell Signaling	# 12691	1:1000
Actin	Santa Cruz Biotechnology	Sc-1616	1:1000
PDI	Cell Signaling	# 3501	1:1000
Keap1	Protein Tech.	10503-2	1:1000
p-ERK	Cell Signaling	# 4370	1:2000
ERK	Cell Signaling	# 4695	1:1000
p-JNK	Cell Signaling	# 4668	1:500
JNK	Santa Cruz Biotechnology	Sc-572	1:1000
β-Tubulin	Santa Cruz Biotechnology	Sc-55529	1:1000
Cleaved IL-1β	Cell Signaling	# 83186	1:1000
IL-1β	Cell Signaling	# 2022	1:500

Supplementary Table 27. Data Collection, Phasing and Refinement Statistics

**Data Collection Statistics**			
**Data Set**	**GSTO1:C1-27**	**GSTO1:C1-31**	**GSTO1:C4-10**
Synchrotron Beamline	21-ID-D	21-ID-G	21-ID-G
Wavelength (Å)	1.0781	0.9786	0.9786
Space Group	C2	$P2_1$	C2
Unit Cell			
a (Å)	182.838	62.645	186.320
b (Å)	71.247	72.588	71.373
c (Å)	61.852	65.310	61.968
β (°)	104.08	112.69	105.16
Resolution (Å)[1]	40.70 – 2.4 (2.44 – 2.40)	45.22 – 1.94 (1.97 - 1.94)	44.96 - 2.10 (2.14 – 2.10)
Rmerge (%)[2]	0.083 (0.245)	0.074 (0.343)	0.078 (0.316)
<I/σI>[3]	10 (5)	5 (5)	10 (3)
Completeness (%)[4]	99.5 (100)	97.5 (98.8)	98.0 (99.3)
Redundancy	5.1 (5.1)	3.1 (2.9)	5 (4.9)
**Refinement Statistics**			
Resolution (Å)[5]	2.38	1.94	2.10
R-work/Rfree[6]	0.2053/0.2366	0.2028/0.2403	0.1950/0.220
Protein atoms[7]	5584	3782	5604
Inhibitor Molecules	3	2	3
Water Molecules	121	285	405
Unique Reflections	30710	36729	46430
R.m.s.d.[8]			
Bonds	0.009	0.010	0.009
Angles	0.95	0.94	0.92
MolProbity Score[9]	0.92	0.99	0.66
Clash Score[9]	1.34	1.7	0.45
**Ligand Statistics**			
Real-space R-value[10]	0.20/0.16/0.12	0.145/0.198	0.19/0.17/0.13
Real-space correlation coefficient[10]	0.88/0.92/0.95	0.927/0.900	0.84/0.87/0.89

[1]Statistics for highest resolution bin of reflections in parentheses.

[2]$R_{merge} = \Sigma_h \Sigma_j \mid I_{hj} - \langle I_h \rangle \mid / \Sigma_h \Sigma_j I_{hj}$, where I_{hj} is the intensity of observation j of reflection h and $\langle I_h \rangle$ is the mean intensity for multiply recorded reflections.

[3]Intensity signal-to-noise ratio.

[4]Completeness of the unique diffraction data.

[5]Resolution cut-off used during heavy-atom refinement and phase calculations.

[6]R-factor = $\Sigma_h \mid |F_o| - |F_c| \mid / \Sigma_h |F_o|$, where F_o and F_c are the observed and calculated structure factor amplitudes for reflection h. R_{free} is calculated against a 5 % random sampling of the reflections that were removed before structure refinement.

[7]Total number of protein atoms refined in the asymmetric unit.

[8]Root mean square deviation of bond lengths and bond angles.

[9] Chen, V. B. *et al*. MolProbity: all-atom structure validation for macromolecular crystallography. *Acta Crystallogr D Biol Crystallogr* **66**, 12-21 (2010).

[10]Kleywegt, G. J. *et al.* The Uppsala Electron-Density Server. *Acta Crystallogr. D Biol. Crystallogr.* **60**, 2240-2249 (2004).

Supplementary Methods

1. Synthesis of compounds for pilot screening

Synthetic procedures for compounds (2a-6). To a stirred solution of amine (4 mmol) in *N,N*-dimethylformamide (3 mL), chloroacetyl chloride (4.8 mmol; 9.6 mmol in case of diamine) was added dropwise, slowly cooling with ice so that the temperature did not rise above 5-7 °C. The mixture was stirred for 1.5 h and then diluted with water. The resulting precipitate was filtered off, washed with water, and dried. Further purification was performed by flash column chromatography using 1:1 EtOAc:hexane as the eluent.

Characterization. Chemical shifts (δ) of ^1H NMR and ^{13}C NMR spectra are reported in parts per million (ppm) units relative to residual undeuterated solvent. The following abbreviations were used to describe peak splitting patterns when approaraite: s (singlet), d (doublet), t (triplet), m (multiplet), bs (broad singlet). Coupling constants (J) are expressed in hertz unit (Hz). Mass spectra using the electron spray ionization (ESI) method was also recorded. Sample purity was further confirmed by elemental analysis.

***N-(2,4-Difluorophenyl)-2-chloroacetamide* (2a)**
Yield 91%; mp: 80-82 °C; ^1H NMR (300 MHz, DMSO-d_6): δ 4.18 (s, 2H, CH_2), 7.20-7.65 (m, 3H, ArH), 9.95 (s, 1H, NH); MS (m/z): 205 $[M]^+$; analysis (calcd., found for $C_8H_6ClF_2NO$): C (46.74, 46.81), H (2.94, 6.98), N (6.81, 6.60).

***N-(2,4-difluoro-6-nitrophenyl)-2-chloroacetamide* (2b)**
Yield 87%; mp: 122-123 °C; ^1H NMR (300 MHz, DMSO-d_6): δ 4.45 (s, 2H, CH_2), 8.18 (m, 1H, ArH), 7.81 (m, 1H, ArH), 10.8 (s, 1H, NH); MS (m/z): 250 $[M]^+$; analysis (calcd., found for $C_8H_5ClF_2N_2O_3$): C (38.34, 38.20), H (2.01, 1.92), N (11.18, 10.98).

***N-(3,4-difluoro-6-nitrophenyl)-2-chloroacetamide* (2c)**
Yield 97%; mp: 130-132 °C; ^1H NMR (300 MHz, DMSO-d_6): δ 4.41 (s, 2H, CH_2), 8.0 (m, 1H, ArH), 8.35 (m, 1H, ArH), 10.8 (s, 1H, NH); MS (m/z): 250 $[M]^+$; analysis (calcd., found for $C_8H_5ClF_2N_2O_3$): C (38.34, 38.27), H (2.01, 2.12), N (11.18, 11.09).

4,5-difluoro -1,2-bis-(2-chloro-acetylamino)-benzene* (4a*)
Yield 96%; mp: 142-143 °C; ^1H NMR (300 MHz, DMSO-d_6): δ 4.33 (s, 4H, CH_2), 7.65 (m, 2H, Ar-H.), 9.78 (s, 2H, NH); MS (m/z): 297 $[M]^+$; analysis (calcd., found for $C_{10}H_8Cl_2F_2N_2O_2$): C (40.43, 40.32), H (2.71, 2.63), N (9.43, 9.57).

***3,5-difluoro -1,2-bis-(2-chloro-acetylamino)-benzene* (4b)**
Yield 96%; mp: 136-138 °C; ^1H NMR (300 MHz, DMSO-d_6): δ 4.24 (s, 4H, CH_2), 7.01 (m, 1H, Ar-H.), 7.92 (m, 1H, Ar-H.), 9.72 (s, 2H, NH); MS (m/z): 297 $[M]^+$; analysis (calcd., found for $C_{10}H_8Cl_2F_2N_2O_2$): C (40.43, 40.28), H (2.71, 2.61), N (9.43, 9.59).

***1,2-Bis-(2-chloro-acetylamino)-benzene* (4c)**
Yield 79%; mp: 196-197 °C; ^1H NMR (300 MHz, $CDCl_3$): δ 8.66 (s, 2H, N-H), 7.54 (m, 2H, Ar-H), 7.31 (m, 2H, Ar-H), 4.22 (s, 4H, CH_2Cl); MS (m/z): 261 $[M]^+$; ^{13}C NMR (75 MHz, DMSO-*d6*): δ 165.7, 130.6, 126.2, 125.6, 43.6; analysis (calcd., found for $C_{10}H_{10}Cl_2N_2O_2$): C (46.00, 46.19), H (3.86, 4.01), N (10.73, 10.57).

***1,4-Bis-(2-chloro-acetylamino)-benzene* (6)**
Yield 83%; mp: 211-213 °C; ^1H NMR (300 MHz, DMSO-d_6): δ 9.66 (s, 2H, N-H), 7.52-7.50 (m, 4H, Ar-H), 4.23 (s, 4H, CH_2Cl); MS (m/z): 261 $[M]^+$; ^{13}C NMR (75 MHz, DMSO-*d6*): δ 164.8, 134.8, 120.3, 43.9; analysis (calcd., found for $C_{10}H_{10}Cl_2N_2O_2$): C (46.00; 46.14), H (3.86, 4.03), N (10.73, 10.61).

2. Synthesis of C1-27A (7a) and BODIPY-conjugated C1-27A (7b)

General information. All commercial chemicals and solvents were reagent grade and used without further purification unless otherwise spcified. Analytical thin layer chromatography was performed on Merck precoated plates (silica gel 60 F_{254}) to follow the course of reactions. ^{1}H NMR and ^{13}C NMR spectra were recorded on a Bruker Ultrashield 300 MHz NMR spectrometer or Bruker Ascend 400 MHz spectrometer. Chemical shifts (δ) of NMR are reported in parts per million (ppm) units relative to residual undeuterated solvent. The following abbreviations were used to describe peak splitting patterns when approariate: s (singlet), d (doublet), t (triplet), m (multiplet), bs (broad singlet). Coupling constants (J) are expressed in hertz unit (Hz). Mass spectra were obtained on a Shimadzu LCMS-2020 liquid chromatography mass spectrometer or a Thermo-Scientific LCQ Fleet mass spectrometer using the electron spray ionization (ESI) method. The purity of compounds was determined either by Waters Acquity H class ultra-performance liquid chromatography (UPLC) with the Acquity UPLC BEH C18 column (1.7 µm, 2.1 mm × 50 mm) or by Shimadzu HPLC Test Kit C18 column (3 µm, 4.6 × 50 mm) under the following gradient elution condition: mobile phase A of acetonitrile/water (10-95%), mobile phase B of acetonitrile/water containing 0.1% formic acid (10-100%) or mobile phase C of methanol/water (10-95%). The purity was established by integration of areas of major peaks detected at 254 nm or at 365 nm (**6b**).

5,5-Difluoro-1,3,7,9-tetramethyl-10-(4-nitrophenyl)-*5H*-4λ^4,5λ^4-dipyrrolo[1,2-*c*:2',1'-*f*][1,3,2]diazaborinine (1)

Compound **1** was synthesized following a procedure described in the literature[2]. 4-Nitrobenzaldehyde (306 mg, 2.02 mmol) and 2,4-dimethyl-*1H*-pyrrole (384 mg, 4.04 mmol) were dissolved in dry DCM (50 mL) under N_2. Two drops of TFA were added, and the reaction mixture was stirred at room temperature for 2 h in the dark until TLC monitoring indicated complete consumption of the aldehyde. DDQ (458 mg, 2.02 mmol) in dry DCM (20 mL) was added, and the reaction mixture was stirred at room temperature for an additional 20 min. The reaction mixture was then treated with Et_3N (3 mL) followed by $BF_3.Et_2O$ (3 mL). After stirring for 3 h, the reaction solution was washed with water (6 x 20 mL), dried over Na_2SO_4, and concentrated under reduced pressure. The residue was purified by flash column chromatography (*n*-hexane:DCM = 2:1) to give the crude nitro-BODIPY dye (**1**) as a red semi-solid. The crude product was used in the next step without further purification. For qualitative analysis, the crude product was purified by recrystallization from ethyl acetate/hexane to give pure sample. ^{1}H NMR (400 MHz, $CDCl_3$): δ 8.37 (d, J = 8.4 Hz, 2H), 7.53 (d, J = 8.8 Hz, 2H), 6.01 (s, 2H), 2.55 (s, 6H), 1.35 (s, 6H); ESI-MS (m/z): 368 $[M-H]^-$.

4-(5,5-Difluoro-1,3,7,9-tetramethyl-*5H*-4λ^4,5λ^4-dipyrrolo[1,2-*c*:2',1'-*f*][1,3,2]diazaborinin-10-yl)aniline (2)

Compound **2** was synthesized following a procedure described in the literature[3]. To a stirred solution of compound **1** (300 mg) in EtOH (40 mL), $NH_2NH_2.H_2O$ (0.9 mL) and 10% Pd/C (14 mg) were added. The reaction mixture was heated at reflux for 2 h under N_2, cooled to room temperature and filtered. The solids were washed with DCM (20 mL), and the filtrate was concentrated under reduced pressure. The resulting residue was purified by flash column chromatography (*n*-hexane:DCM = 1:2) to obtain the desired amino-BODIPY dye (**2**) as a red crystal (92 mg, 13% over two steps). ^{1}H NMR (400 MHz, $CDCl_3$): δ 7.01 (d, J = 8.0 Hz, 2H), 6.77 (d, J = 8.0 Hz, 2H), 5.96 (s, 2H), 3.82 (bs, 2H), 2.54 (s, 6H), 1.49 (s, 6H); ESI-MS (m/z): 340 $[M+H]^+$, 320 $[M-F]^+$.

5-((((*9H*-Fluoren-9-yl)methoxy)carbonyl)amino)-2,4-dimethylbenzenesulfonic acid (3)

To 9-fluoreneylmethyl chloroformate (4.17 g, 16.1 mmol), 2,4-dimethylaniline-5-sulfonic acid sodium salt (3 g, 13.4 mmol) and water (25 mL) were added. The reaction mixture was stirred at 60℃ for 4 h until TLC indicated complete consumption of the amine. The mixture was washed with 1N HCl and extracted with EtOAc. The organic fractions were dried over Na_2SO_4, and concentrated under reduced pressure. The resulting residue was purified by flash column chromatography (MeOH:DCM = 1:5) to afford the compound (**3**) as a yellow solid (3 g, 53%). ^{1}H NMR (400 MHz, DMSO-*d6*): δ 8.94 (bs, 1H), 7.90 (d, J = 7.6 Hz, 2H), 7.74 (bs, 2H), 7.61 (s, 1H), 7.43 (t, J = 7.2 Hz, 2H), 7.35 (t, J = 7.6 Hz, 2H), 6.96 (s, 1H), 4.37 (bs, 2H), 4.28 (bs, 1H), 2.46 (s, 3H), 2.13 (s, 3H); ESI-MS (m/z): 422 $[M-H]^-$.

(*9H*-Fluoren-9-yl)methyl (5-(chlorosulfonyl)-2,4-dimethylphenyl)carbamate (4)

Compound **4** was synthesized following a procedure described in the literature[4]. To a solution of compound **3** (3 g, 7.09 mmol) in DMF (4 mL), thionyl chloride (4.38 g, 36.8 mmol) was added. The reaction mixture was stirred at room temperature for overnight, and then quenched by the addition of water. The resulting precipitate was collected by filtration to give the compound (**4**) as a white solid (2.49 g, 80%). ^{1}H NMR (400 MHz, DMSO-*d6*): δ 8.94 (bs, 1H), 7.90 (d, J = 7.2 Hz, 2H), 7.74 (bs, 2H), 7.61 (s, 1H), 7.43 (t, J = 7.2 Hz, 2H), 7.35 (t, J = 7.6 Hz, 2H), 6.97 (s, 1H), 4.37 (bs, 2H), 4.28 (bs, 1H), 2.46 (s, 3H), 2.13 (s, 3H); ESI-MS (m/z): 464 $[M+Na]^+$.

(9*H*-Fluoren-9-yl)methyl (5-(*N*,*N*-dimethylsulfamoyl)-2,4-dimethylphenyl)carbamate (5a)

To a solution of the carbamate **4** (200 mg, 0.45 mmol) in dry DCM (6 mL), *N*,*N*-diemthylamide (2M in THF) (0.34 mL, 0.68 mmol) and pyridine (72 mg, 0.91 mmol) were added. The reaction mixture was stirred at room temperature overnight under N_2 until TLC indicated complete consumption of the carbamate, and then concentrated under reduced pressure. The resulting residue was purified by flash column chromatography (EtOAc: *n*-hexane = 1:3) to afford the compound (**5a**) as a white solid (177 mg, 87%). HPLC (mobile phase A): purity 98.4%; ^{1}H NMR (400 MHz, $CDCl_3$): δ 8.14 (bs, 1H), 7.77 (d, J = 7.6 Hz, 2H), 7.58 (bs, 2H), 7.41 (t, J = 7.6 Hz, 2H), 7.32 (t, J = 7.2 Hz, 2H), 7.12 (s, 1H), 6.38 (bs, 1H), 4.54 (d, J = 6.8 Hz, 2H), 4.27 (t, J = 6.8 Hz, 1H), 2.84 (s, 6H), 2.57 (s, 3H), 2.25 (s, 3H); ^{13}C NMR (75 MHz, $CDCl_3$): δ 153.7, 143.6, 141.3, 134.8, 134.3, 133.5, 127.8, 127.1, 124.9, 120.0, 67.1, 47.1, 37.4, 20.0, 17.5; ESI-MS (m/z): 451 $[M+H]^+$, 473 $[M+Na]^+$.

(*9H*-Fluoren-9-yl)methyl (5-(*N*-(4-(5,5-difluoro-1,3,7,9-tetramethyl-*5H*-4λ^4,5λ^4-dipyrrolo[1,2-*c*:2',1'-*f*][1,3,2]diazaborinin-10-yl)phenyl)sulfamoyl)-2,4-dimethylphenyl)carbamate (5b)

Compound **5b** was synthesized using the general procedure for the synthesis of **5a** with the carbamate **4** (43 mg, 0.098 mmol) in dry DCM (3 mL), the amino-BODIPY dye **2** (50 mg, 0.15 mmol) and pyridine (16 mg, 0.196 mmol) to obtain the compound (**5b**) as a solid (75 mg, quantitative) after purification by flash column chromatography (*n*-hexane:DCM = 1:2 to EtOAc: *n*-hexane = 1:3). HPLC (mobile phase C): purity 98.8%; ^{1}H NMR (400 MHz, $CDCl_3$): δ 8.32 (bs, 1H), 7.77 (d, J = 7.6 Hz, 2H), 7.57 (d, J = 7.2 Hz, 2H), 7.41 (t, J = 7.6 Hz, 2H), 7.31 (t, J = 7.6 Hz, 2H), 7.19 (d, J = 7.2 Hz, 2H), 7.10 (d, J = 8.0 Hz, 2H), 7.05 (s, 1H), 6.80 (bs, 1H), 6.34 (s, 1H), 5.88 (s, 2H), 4.52 (d, J = 6.4 Hz, 2H), 4.25 (t, J = 6.4 Hz, 1H), 2.57 (s, 3H), 2.51 (s, 6H), 2.22 (s, 3H), 1.20 (s, 6H); ^{13}C NMR (75 MHz, $CDCl_3$) δ: 155.5, 143.5, 142.8, 141.3, 140.6, 137.5, 135.0, 134.5, 133.8, 131.4, 131.3, 131.3, 129.0, 127.8, 127.1, 124.9, 121.2, 121.1, 120.1, 67.2, 47.0, 19.8, 17.6, 14.5, 14.2; ESI-MS (m/z): 745 $[M+H]^+$, 725 $[M-F]^+$.

5-Amino-*N*,*N*,2,4-tetramethylbenzenesulfonamide (6a)

To a solution of the compound **5a** (150 mg, 0.33 mmol) in DMA (4 mL), piperidine (85 mg, 1.0 mmol) was added. The reaction mixture was stirred at room temperature for 1 h under N_2, and then diluted with water. The organic phase was extracted with EtOAc, and the combined organic fractions were dried over Na_2SO_4 and concentrated under reduced pressure. The resulting residue was purified by flash column chromatography (EtOAc:*n*-hexane = 1:3) to afford the compound (**6a**) as a white crystal (68 mg, 89%). HPLC (mobile phase A): purity 99.6%; ^{1}H NMR (400 MHz, $CDCl_3$): δ 7.21 (s, 1H), 6.97 (s, 1H), 3.65 (bs, 2H), 2.76 (s, 6H), 2.41 (s, 3H), 2.17 (s, 3H); ^{13}C NMR (75 MHz, $CDCl_3$): δ 142.5, 134.7, 133.2, 127.4, 127.1, 116.4, 37.0, 19.5, 17.1; ESI-MS (m/z): 229 $[M+H]^+$.

5-Amino-*N*-(4-(5,5-difluoro-1,3,7,9-tetramethyl-*5H*-4λ^4,5λ^4-dipyrrolo[1,2-*c*:2',1'-*f*][1,3,2]diazaborinin-10-yl)phenyl)-2,4-dimethylbenzenesulfonamide (6b)

The procedure for the synthesis of **6a** was used with compound **5b** (75 mg, 0.10 mmol) in DMA (2 mL) and piperidine (26 mg, 0.30 mmol) to obtain the compound (**6b**) as a red solid (50 mg, 96%) after extraction and purification by flash column chromatography (EtOAc:*n*-hexane = 1:3). HPLC (mobile phase A): purity 97.9%; ^{1}H NMR (400 MHz, DMSO-*d6*): δ 10.28 (bs, 1H), 7.20 – 7.13 (m, 5H), 6.82 (s, 1H), 6.12 (s, 2H), 5.02 (s, 2H), 2.41 (s, 6H), 2.37 (s, 3H), 2.08 (s, 3H), 1.17 (s, 6H); ^{13}C NMR (75 MHz, $CDCl_3$) δ: 155.1, 145.0, 143.2, 142.0, 139.3, 134.5, 134.1, 131.2, 129.1, 128.8, 126.7, 122.7, 121.7, 120.1, 115.6, 19.0, 17.5, 14.6, 14.2; ESI-MS (m/z): 523 $[M+H]^+$, 503 $[M-F]^+$.

2-Chloro-*N*-(5-(*N*,*N*-dimethylsulfamoyl)-2,4-dimethylphenyl)acetamide (C1-27A) (7a)

To a solution of the compound **6a** (50 mg, 0.22 mmol) in dry DCM (2 mL), Et_3N (23 mg, 0.22 mmol) was added, and the mixture was stirred at room temperature for 10 min. Chloroacetyl chloride (25 mg, 0.22

mmol) in dry DCM (2 mL) was added dropwise and the reaction mixture was stirred for 8 h. The mixture was diluted with water and extracted with DCM. The combined organic fractions were washed with brine, dried over Na_2SO_4, and concentrated under reduced pressure. The resulting residue was purified by flash column chromatography (EtOAc:*n*-hexane = 1:3) to afford the desired **C1-27A** (**7a**) as an ivory solid (55 mg, 82%). HPLC (mobile phase C): purity 99.8%; ^{1}H NMR (400 MHz, $CDCl_3$): δ 8.28 (bs, 1H), 8.20 (s, 1H), 7.15 (s, 1H), 4.22 (s, 2H), 2.83 (s, 6H), 2.55 (s, 3H), 2.30 (s. 3H); ^{13}C NMR (75 MHz, $CDCl_3$): δ 164.1, 135.1, 134.9, 134.5, 134.3, 132.5, 123.8, 43.1, 37.4, 20.1, 17.4. ESI-MS (m/z): 305 $[M+H]^+$, 327 $[M+Na]^+$.

2-Chloro-*N*-(5-(*N*-(4-(5,5-difluoro-1,3,7,9-tetramethyl-*5H*-4λ^4,5λ^4-dipyrrolo[1,2-*c*:2',1'-*f*][1,3,2]diazaborinin-10-yl)phenyl)sulfamoyl)-2,4-dimethylphenyl)acetamide (BODIPY-conjugated C1-27A) (7b)

The procedure applied to the synthesis of **7a** was used with the compound **6b** (50 mg, 0.096 mmol) in dry DCM (2 mL), Et_3N (20 mg, 0.19 mmol), and chloroacetyl chloride (22 mg, 0.19 mmol) in dry DCM (2 mL) to obtain the desired BODIPY-conjugated product (**7b**) as a red solid (31 mg, 54%) after extraction and purification by flash column chromatography (DCM to EtOAc:*n*-hexane = 1:3). UPLC (mobile phase B): purity 97.4%; ^{1}H NMR (400 MHz, DMSO-*d6*): δ 10.58 (bs, 1H), 9.71 (s, 1H), 8.00 (s, 1H), 7.21 (d, J = 8.8 Hz, 2H), 7.19 (s, 1H), 7.14 (d, J = 8.4 Hz, 2H), 6.12 (s, 2H), 4.29 (s, 2H), 2.53 (s, 3H), 2.42 (s, 6H), 2.18 (s, 3H), 1.14 (s. 6H); ^{13}C NMR (75 MHz, DMSO-*d6*): δ 165.5, 155.2, 143.1, 141.8, 138.9, 137.4, 134.7, 134.6, 134.5, 133.8, 133.7, 133.6, 131.2, 129.4, 129.0, 126.6, 121.7, 120.3, 43.5, 19.5, 17.9, 14.6, 14.1; ESI-MS (m/z): 579 $[M-F]^+$.

Abbreviations used

DMF, *N*,*N*-dimethylforamide; EtOAc, Ethyl acetate; $BF_3.Et_2O$, boron trifluoride diethyl etherate; DDQ, 2,3-dichloro-5,6-dicyano-1,4-benzoquinone; DCM, dichloromethane; DMA, *N*,*N*-dimethylacetamide; Et_3N, triethylamine; TFA, trifluoroacetic acid; THF, tetrahydrofuran.

Supplementary References

1 Roversi P., Sharff A., Smart O.S., Vonrhein C. & T.O., W. BUSTER Version 2.11.2. *Cambridge, United Kingdom: Global Phasing Ltd.* (2011).

2 Pan, Z.-H. *et al.* A simple BODIPY-aniline-based fluorescent chemosensor as multiple logic operations for the detection of pH and CO_2 gas. *Dalton Transactions* **43**, 8499-8507 (2014).

3 Yu, M. *et al.* Efficient deprotection of *F*-BODIPY derivatives: removal of BF_2 using Brønsted acids. *Beilstein J. Org. Chem.* **11**, 37-41 (2015).

4 Siu, T. *et al.* Inhibitors of janus kinases. US 8349865 B2 (2013).
